# PLZF promotes compensatory lung growth by increasing HPMEC proliferation and angiogenesis

**DOI:** 10.1371/journal.pone.0325936

**Published:** 2025-07-02

**Authors:** Jing Peng, Liang Ma, Zhonghui Wang, Yaxi Du, Qunfen Tao, Qiongchuan Wang, Li Zhao

**Affiliations:** 1 Department of Anesthesiology, Yunnan Cancer Hospital, The Third Affiliated Hospital of Kunming Medical University, Peking University Cancer Hospital Yunnan, Kunming, Yunnan, China; 2 Department of Molecular Diagnosis Center, Yunnan Cancer Hospital, The Third Affiliated Hospital of Kunming Medical University, Peking University Cancer Hospital Yunnan, Kunming, Yunnan, China; 3 Department of Operation Room, Yunnan Cancer Hospital, The Third Affiliated Hospital of Kunming Medical University, Peking University Cancer Hospital Yunnan, Kunming, Yunnan, China; Thomas Jefferson University, UNITED STATES OF AMERICA

## Abstract

Angiogenic signaling pathway activation has been shown to accelerate compensatory lung growth (CLG) after unilateral pneumonectomy (PNX). Therefore, studying specific genes regulating angiogenic signaling pathways is a novel strategy to promote CLG. EdU, flow cytometry and tube formation experiments were performed to test the metabolism of human pulmonary microvascular endothelial cells (HPMECs). Western blotting was used to analyze the levels of promyelocytic leukemia zinc finger protein (PLZF), kelch-like ECH-associated protein 1 (Keap1), hypoxia-inducible factor-1α (HIF-1α), hemeoxygenase-1 (HO-1), quinone oxidoreductase (NQO1), nuclear factor E2-related factor 2 (Nrf2) and other proteins. The proliferation of pulmonary endothelial cells was assessed by Ki67 double staining. A unilateral PNX mouse model was constructed, and changes in lung volume and weight were assessed. Our bioinformatics results suggested that PLZF showed a clear downward trend after unilateral PNX. PLZF overexpression significantly promoted HPMECs proliferation and angiogenesis and inhibited their apoptosis. Further studies revealed that both Keap1 overexpression and Nrf2 silencing altered the effects of PLZF overexpression on HPMECs and inhibited their apoptosis. Notably, HIF-1α silencing reversed the effect of PLZF overexpression on HPMECs angiogenesis but not on proliferation or apoptosis. Knockdown of Nrf2 not only affected HPMECs proliferation and apoptosis but also affected angiogenesis. An in vivo study confirmed that PLZF overexpression promoted an increase in residual lung volume and lung weight in mice after unilateral PNX and significantly promoted the proliferation of lung endothelial cells. In conclusion, our study revealed that PLZF promotes HPMECs proliferation and angiogenesis and accelerates CLG by inhibiting Keap1 activation of the Nrf2 and HIF-1α/VEGF signaling pathways.

## 1. Introduction

Due to an unhealthy modern lifestyle, the prevalence rate of lung disease, especially among smokers, remains high, with chronic obstructive pulmonary disease and lung cancer accounting for a relatively high proportion of lung disease [1]. Pneumonectomy (PNX) is often the last resort treatment for patients with lung cancer [2]. Studies have shown that unilateral PNX initiates rapid growth of the remaining lung mass, a phenomenon known as compensatory lung growth (CLG) [3]. CLG after unilateral PNX has been confirmed in mice [4], rats [5], pigs [6], dogs [7], rabbits [8], ferrets [9] and humans [10]. Triggering CLG after PNX results not only in an increase in lung volume but also in an increase in the number of alveoli, and a key mechanism for new alveoli is neovascularization [11]. It has recently been shown that the number of pulmonary endothelial cells (ECs) is significantly increased after unilateral PNX in mice, and that blood-borne CD34 + endothelial progenitor cells (EPCs) become resident ECs during CLG and contribute to pulmonary vascularization [12]. Therefore, EC has a unique function in promoting angiogenesis and accelerating CLG after unilateral PNX.

Promyelocytic leukemia zinc finger protein (PLZF), also known as zinc finger and BTB domain-containing 16 (ZBTB16), belongs to the Krüppel-like zinc finger protein family [13]. As a type of transcription factor, PLZF has a unique regulatory function in the growth and development of the body, with a particular focus on the development of organs and cells, such as the physiological metabolism of immune cells and stem cells [14]. In recent years, studies have shown that PLZF may be associated with the Kelch-like ECH-associated protein 1 (Keap1)- nuclear factor E2-related factor 2 (Nrf2) pathway. Although there is no direct evidence that PLZF interacts directly with Keap1, PLZF may inhibit Keap1 activity by regulating the Nrf2 transcription level or its binding affinity with Keap1. The inhibitory effect of Keap1 on Nrf2 was relieved, and the activity of Nrf2 was subsequently affected [15]. However, the pathway by which PLZF regulates endothelial cell function and plays a role in promoting CLG is unknown.

Nrf2 is a transcription factor that has critical functions in maintaining a healthy endothelial phenotype and maintaining the functional integrity of the vasculature [16]. Keap1 is a specific inhibitor of Nrf2 [17]. Nrf2 is ubiquitinated and degraded under physiological conditions [18]. Under normal physiological conditions, Keap1 maintains the intracellular redox balance by promoting the ubiquitination and degradation of Nrf2. However, when cells are exposed to oxidative stress or electrophilic stimulation, the inhibitory effect of Keap1 on Nrf2 is lifted, and Nrf2 can be stabilized and transferred to the nucleus, where it can activate the expression of a series of antioxidants (such as HO-1 and NQO1) and cytoprotection-related genes [19]. The Keap1‒Nrf2 pathway is a key defense system involved in maintaining homeostasis in mammalian cells [18]. Nrf2 has been reported to contribute to the angiogenic potential of ECs and proangiogenic cells [20] and to regulate EC proliferation [21,22]. In addition, Keap1 is also involved in regulating the stability of hypoxia-inducible factor-1α (HIF-1α). Under normoxic conditions, HIF-1α is modified by proline hydroxylase (PHD2) through the hydroxylation of proline residues and then binds to pVHL and is degraded by the proteasome [23]. Under hypoxia, Keap1 affects the redox state of cells by regulating the activity of Nrf2 and then affects the stability of HIF-1α [24]. Vascular growth factor (VEGF) is a growth factor that can promote angiogenesis, activate the physiological activity of endothelial cells and maintain the steady state of the vascular system [25]. In addition, HIF-1α can also be involved in VEGF-mediated angiogenesis under hypoxic conditions [26]. Many studies have shown that the HIF-1α/VEGF signaling pathway has unique functions in angiogenesis and tumor growth [27,28].

The aim of this study was to describe the effects of PLZF on proliferation, apoptosis, and angiogenesis in HPMECs and the underlying mechanisms and to validate the ability of PLZF overexpression to promote CLG at the animal level. Taken together, our results provide a rationale for the importance of specific genes in promoting CLG and function.

## 2. Materials and methods

### 2.1. Surgical model and experimental groups

In this study, 8-week-old male C57BL/6 mice (obtained from the Animal Experimental Center of Kunming Medical University) were used for the experiment. The surgical model was prepared as previously described by Duy T et al. [29]. Avertin (120–400 mg/kg; Sigma, St. Louis, MO) was used for anesthesia. A 4−0 silk thread (Ethicon, Somerville, NJ) was used to ligate the hilum. PDS® (Ethicon, Somerville, NJ) was used for suturing. The animals were administered buprenorphine twice daily for three days after surgery. The mice were randomly divided into the normal control group, PNX group, PNX + PcDNA3.1 vector group and PNX + PcDNA3.1-PLZF group, with n = 5/group. The PcDNA3.1-PLZF group was intraperitoneally injected with PcDNA3.1-PLZF overexpression lentiviral vector at a dose of 1 mg/mL/kg every day and normal saline was administered to the normal control group and PNX group (Vector construction information: Based on the third-generation lentivirus system, by Dalian Takara Bio, Inc. The PLZF cDNA sequence was referred to NCBI accession number NM_001312662.1. The vector backbone contained CMV promoter and puromycin resistance marker). Intraperitoneal injection was chosen as the delivery route because of its good systemic distribution characteristics and easy handling, and previous experiments showed that this route had no obvious toxicity to mice at this dose. After 7 days of treatment, the mice were anesthetized with ketamine (80–100 mg/kg) and xylazine (10–12.5 mg/kg), and the remaining lung tissue was removed for subsequent analysis. All experiments followed the ARRIVE guidelines and were approved by the Animal Ethics Committee of Kunming Medical University (kmmu2020272).

### 2.2. Lung weight and body weight measurements

The remaining lung tissue removed was weighed and photographed, and the body weights of the mice were also recorded. The ratio of the weight of the remaining lung tissue to the body weight of the mouse was calculated.

### 2.3. Immunofluorescence

Immunofluorescence staining was used to detect the expression and distribution of Ki67 and ERG. The sample pieces to be tested were collected, washed with PBS, and then fixed in 4% paraformaldehyde for 15 min. The diluted primary antibodies against Ki67 (1:250, ab16667, Abcam, UK) and ERG (1:100, ab214796, Abcam, UK) were added at 37°C for 30 min, followed by immersion in PBS and washing 3 times. An Alexa Fluor® 488-labeled goat anti-rabbit IgG (H + L) secondary antibody (1:1,000, ab150077, Abcam, UK) was added dropwise, and the mixture was incubated at 37°C for 30 min. DAPI was added dropwise to stain the nuclei, and the mixture was incubated in the dark for 5 min. The slides were then sealed. The images were observed and collected under a fluorescence microscope.

### 2.4. Cell culture and transfection

Human pulmonary microvascular endothelial cells (HPMECs) were obtained from the Cell Bank of the Chinese Academy of Science (Shanghai, China) and cultured in a mixture containing RPMI-1640, 10% fetal bovine serum (HyClone; GE Healthcare Life Sciences, Logan, UT, USA), 100 IU/ml penicillin and 100 μg/ml streptomycin (Sangon Biotech Co., Ltd., Shanghai, China). The medium was changed every 2 d, and the cells were passaged every 4 d. After resuscitation, the cells were transferred to 3–5 generations for the experiment.

The cells were seeded at the optimal density in 6-well plates 24 h prior to transfection and then incubated overnight. PcDNA3.1-PLZF, PcDNA3.1-Keap1 and PcDNA3.1-vector were designed and cloned by Takara Bio, Inc. (Dalian, China). The small interfering RNAs si-Nrf2 and si-HIF-1α, which target Nrf2 and HIF-1α, respectively, were designed and purchased from Gene Pharma, China. PLZF, Keap1, si-NC, si-Nrf2 and si-HIF-1α were transfected into HPMECs in groups using Lipofectamine™ 3000 reagent (L3000075, Invitrogen, USA), and the untransfected cells were used as the control group. The transfection efficiency was measured 48 h later.

### 2.5. Cell proliferation assay using Cell Counting Kit-8 (CCK-8)

The HPMECs were assessed via CCK-8 (BioVision, Mountain View, CA, USA). The cell suspension was seeded into a 96-well plate at a rate of 5 × 10³ cells/well and precultured in a cell incubator at 37°C with 5% CO₂ for 24 h. 10 μL of CCK-8 solution was added to each well and incubated for 2 h. The absorbance was measured at a wavelength of 450 nm using a microplate reader (Bio-Rad Laboratories, USA).

### 2.6. Ethynyl-2′-deoxyuridine (EdU) assay

To assess the proliferative capacity of HPMECs, EdU assays were performed using an EdU cell proliferation kit containing Alexa Fluor488 (C0071; Beyotime, Shanghai, China) according to the manufacturer’s instructions. 48 h after transfection, the cells were incubated with 10 μM EdU for 2 h; then, the samples were incubated with Alexa Fluor 488 azide for 30 min, and the nuclear DNA was stained with 4,6-diamino-2-phenylindole (DAPI; Beyotime). Finally, the number of EdU-positive cells was determined via fluorescence microscopy.

### 2.7. Tube formation assay

Cells were starved with medium containing 0.5% fetal bovine serum (FBS) for 4 h before experiments. Matrigel was placed in a refrigerator at 4°C overnight to allow it to thaw thoroughly. The next day, the 96-well plate and pipette tip were cooled for 1 h at 4°C. The plate and tip were kept on ice throughout the experiment. Primary Matrigel (60 μL) was loaded into each well and incubated at 37°C in an incubator with 5% CO_2_ for 30 min to allow matrix polymerization. Pretreated HPMECs were resuspended and recounted to achieve an appropriate cell density (2 × 10^4^ cells/well). HPMECs were starved by removing the serum for 4 h prior to tube formation assays in medium without antibiotics. After the Matrigel was set, a preprepared cell suspension was gently dripped onto each well of the Matrigel. The plates were incubated at room temperature for 15 min and then transferred to an incubator at 37°C. After 5 h of incubation, the number of knots/closed loops of tubular structures in five randomly selected light fields was determined via an Olympus BX51 + DP70 fluorescence microscope (Olympus Corporation, Tokyo, Japan).

### 2.8. Apoptosis detection by flow cytometry

HPMECs were cultured in DMEM medium containing 10% FBS and placed in an incubator at 37°C with 5% CO₂ until the cells reached the logarithmic growth phase. HPMECs were collected, washed by addition of precooled PBS, cells were resuspended in buffer, centrifuged at 1000 rpm for 5 min, and the supernatant was discarded. The cells were incubated with Annexin V/FITC and PI for 15 min at 4°C in the dark in accordance with the operating guidelines of the Annexin V-FITC/PI Apoptosis Detection Kit (V13242, Invitrogen, USA). The level of apoptosis in the cells was analyzed using a FACScalibur flow cytometer (BD Biosciences, Franklin Lakes, NJ, USA).

### 2.9. Western blotting

Proteins were extracted from cells and tissues using a whole-cell lysis kit (CWBio, Beijing, China), and protein concentrations were measured using a BCA protein quantification kit (Beyotime Biotechnology). After denaturation by boiling, protein samples (40 μg per lane) were separated by sodium dodecyl sulfate‒polyacrylamide gel electrophoresis (SDS‒PAGE) and transferred to polyvinylidene difluoride (PVDF) membranes (Millipore, Boston, MA). After blocking with 5% skim milk (YILI, Hohhot, Inner Mongolia, China) for 1 h at room temperature, the membranes were incubated overnight at 4°C with the following antibodies: PLZF (ab305064, 1:1,000, Abcam, U K), Keap1 (ab119403, 1:500, Abcam, UK), Nrf2 (PA5−27882, 1:1,000, ThermoFisher Scientific), NQO1 (ab80588, 1:10,000, Abcam, UK), HO-1 (ab189491, 1:2,000, Abcam, UK), HIF-1α (ab308433, 1:1,000, Abcam, UK), VEGF (ab32152, 1:1,000, Abcam, UK), VEGFR-2 (ab315238, 1:1,000, Abcam, UK), FGF (ab208687, 1:1,000, Abcam, UK), CD31 (ab9498, 1:1,000, Abcam, UK), CD34 (ab110643, 1:50,000, Abcam, UK) and GAPDH (ab8245, 1:10,000, Abcam, UK), and a secondary antibody HRP conjugated anti-goat anti-rabbit IgG H&L (ab6721, 1:2,000, Abcam, UK) and HRP conjugated anti-goat anti-mouse IgG H&L (ab6728, 1:10,000, Abcam, UK). After incubation, the membranes were assessed for expression semi quantitatively via enhanced chemiluminescence (ECL) (Millipore, Billerica, MA, USA) chromogenic and gel imaging (version 5.0; Bio-Rad, USA).

### 2.10. RT‒qPCR

Total RNA was extracted from HPMECs using TRIzol reagent (Invitrogen), and RNA was reverse transcribed to cDNA via a first-strand cDNA synthesis kit (Genenode, China). Real-time PCR was performed using a SYBR Green real-time PCR kit (Solarbio, China) with cDNA as the template and GAPDH as the internal reference, and the results were calculated using the 2 − ^ΔΔCt^ method. Details of the primer sequences are shown in Table 1.

**Table 1:** RT-qPCR primer sequences.

Target	Sequence (F:Forward primer;R:Reversed primer) (5´-3´)
PLZF (Mouse)	F: CTGGGACTTTGTGCGATGTG R: CGGTGGAAGAGGATCTCAAACA
Keap1 (Human)	F: CTGGAGGATCATACCAAGCAGG
	R: GGATACCCTCAATGGACACCAC
HIF-1α (Human)	F: GAACGTCGAAAAGAAAAGTCTCG
	R: CCTTATCAAGATGCGAACTCACA
Nrf2 (Human)	F: TCAGCGACGGAAAGAGTATGA
	R: CCACTGGTTTCTGACTGGATGT
GAPDH (Mouse)	F: AGGTCGGTGTGAACGGATTTG
	R: GGGGTCGTTGATGGCAACA
GAPDH (Human)	F: GGAGCGAGATCCCTCCAAAAT
	R: GGCTGTTGTCATACTTCTCATGG

### 2.11. Coimmunoprecipitation

HPMECs were collected and lysed using IP lysis buffer, and the supernatants were extracted by centrifugation. The protein A/G Sepharose, PLZF and Keap1 antibodies were preincubated for 60 min at 4°C with slow shaking and then washed twice. All immunoprecipitate was incubated overnight at 4°C with slow shaking. The beads were collected by centrifugation and then washed three times with lysis buffer. The immunoprecipitate was subjected to immunoblot analysis.

### 2.12. Chromatin coimmunoprecipitation (ChIP)

HPMECs in the logarithmic growth phase were cross-linked with 1% formaldehyde for 10 min. Crosslinking was quenched by addition of 125 mM glycine and maintained at room temperature for 5 min. Samples were sonicated to 200–800 bp and incubated overnight at 4°C for immunoprecipitation with PLZF primary antibody (ab119403, 1:1000, Abcam, UK) or IgG (1:30, ab172730, Abcam, UK). The precipitate was washed with low-salt buffer (1 mL), high-salt buffer (1 mL), LiCl solution (1 mL), and TE buffer (1 mL, 2×). ChIP wash buffer (250 μL, 2×) was added to elution. DNA was de-crosslinked and recovered with 5 M, 20 μL NaCl and subjected to RT-qPCR.

### 2.13. Bioinformatics analysis

The GEO database is a public gene expression database that contains many high-throughput sequencing results. In this study, datasets related to CLG studies were first retrieved from the GEO database, and bioinformatics analysis was performed using the GSE66943 dataset. The mice were divided into a Sham group and a PNX group in the GSE66943 dataset, and the sample grouping and processing met the requirements of this study; therefore, this dataset was selected for our molecular mechanism research.

A unified background check and normalization process were performed on the mRNA data through the Limma package in the R language, and further differential analysis was performed, in which the judgment threshold value of the differential expression was set as *P* < 0.05 and the log2FC was filtered through the statistically significant threshold value in the dataset.

### 2.14. Statistical analysis

The entire dataset was analyzed using GraphPad Prism 8.0 (GraphPad Software, USA), and the results are presented as the mean values along with their standard deviations. In our study, all the experiments were performed independently 3 times or more, and the experimental data are expressed as the mean ± SD. For statistical comparisons, Student’s *t* test was used when there were only two groups with differences between groups. Moreover, one-way ANOVA was used for multiple groups. Overall survival curves were generated using the Kaplan‒Meier method and estimated by the log-rank test. *P* < 0.05 was considered statistically significant. All data are available in the article.

## 3. Results

### 3.1. PLZF is significantly downregulated after PNX

**Fig 1:**
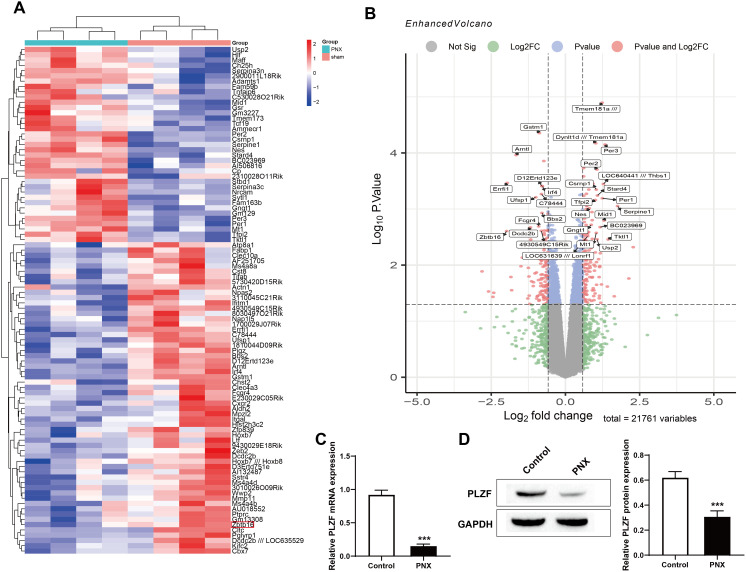
PLZF is significantly downregulated after PNX. A: Heatmap of significantly different genes. B: Volcano map of significantly different genes. C: The expression level of PLZF in lung tissue was determined by RT‒qPCR. D: Western blotting was used to assess the expression level of PLZF in lung tissue. The data are expressed as the mean ± SD (*n *= 5). ***P < 0.001 vs. control.

In this study, a total of 381 DEGs were identified in the GSE66943 dataset, including 180 downregulated genes and 201 upregulated genes. The top 100 most significantly differentially expressed genes (ranked by p value) were analyzed via a protein‒protein interaction (PPI) network (constructed on the basis of the STRING database, https://string-db.org/, confidence level>0.7), which is a global map of protein‒protein interactions constructed by bioinformatics methods. The core idea is to treat proteins as nodes and interactions as edges, thereby forming a complex biological network. PPI network analysis can reveal protein functions and identify key regulatory nodes [30]. We found that the transcription factor (PLZF) ZBTB16 exhibited an extremely high node degree in the network, which was significantly greater than that of other candidate genes, suggesting that it may play a central hub position in the regulatory network (Figs 1A and B). The left lungs of the mice were subsequently excised to establish a unilateral PNX model. The RT‒qPCR and Western blot results revealed that the expression of PLZF in the PNX group was significantly downregulated compared with that in the normal control group (Figs 1C and D). Previous studies have shown that PLZF plays an important role in regulating endothelial cell proliferation [31]. Therefore, PLZF was selected for further study to explore its role in promoting CLG and the underlying molecular mechanism.

### 3.2. PLZF overexpression promotes HPMECs proliferation and angiogenesis

**Fig 2:**
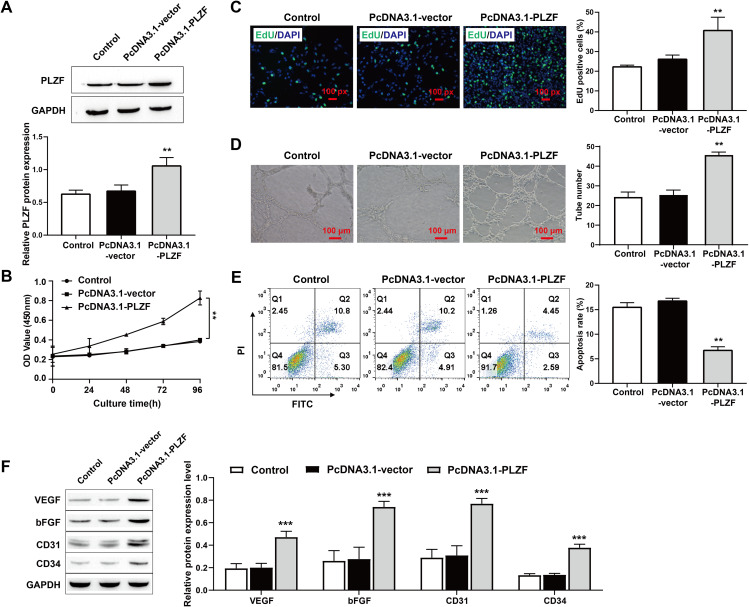
PLZF overexpression promotes HPMECs proliferation and angiogenesis. A: Transfection efficiency of PcDNA3.1-PLZF in HPMECs was assessed by Western blot; B: CCK-8 was used to assess the proliferation ability of HPMECs following overexpression of PLZF; C: EdU staining was used to assess the proliferation activity of HPMECs and quantification of the percentage of EdU-positive cells, scale bars: 100 px; D: Tube formation experiment for the vascularization ability of HPMECs, scale bars: 100 μm; E: Flow cytometry was used to assess apoptosis of HPMECs; F: Western blot was used to assess the level of related proteins in HPMECs. The data are expressed as the mean ± SD (n = 3). **P < 0.01, ***P < 0.001 vs. Control.

Because ECs play an important role in promoting CLG, we first explored the effects of PLZF overexpression on HPMECs proliferation and angiogenesis, as well as apoptosis, in vitro. We constructed an overexpression vector for enhancing the expression of PLZF. The Western blot results revealed that the level of PLZF showed a clear upward trend in the PcDNA3.1-PLZF group but that there was no change in the PcDNA3.1-vector group, confirming the success of the transfection (Fig 2A). The results of the CCK-8 and EdU assays revealed that the proliferation ability of HPMECs significantly improved in the PcDNA3.1-PLZF group, but did not change in the PcDNA3.1-vector (Figs 2B and C). The results of the vascularization experiment clearly revealed that PcDNA3.1-PLZF promoted HPMECs vascularization, but no change was detected in the PcDNA3.1-vector group (Fig 2D). In addition, after flow cytometry experiments, we concluded that the apoptosis rate of HPMECs clearly decreased in the PcDNA3.1-PLZF group but did not change in the PcDNA3.1-vector group (Fig 2E). In addition, the expression levels of angiogenesis markers were assessed, and the results revealed that the expression levels of VEGF, bFGF, CD31, and CD34 in the PcDNA3.1-PLZF group were significantly increased (Fig 2 F). Taken together, these findings suggest that PLZF overexpression can promote HPMECs proliferation and angiogenesis and inhibit their apoptosis.

### 3.3. PLZF promotes HPMECs proliferation and angiogenesis by inhibiting Keap1

**Fig 3:**
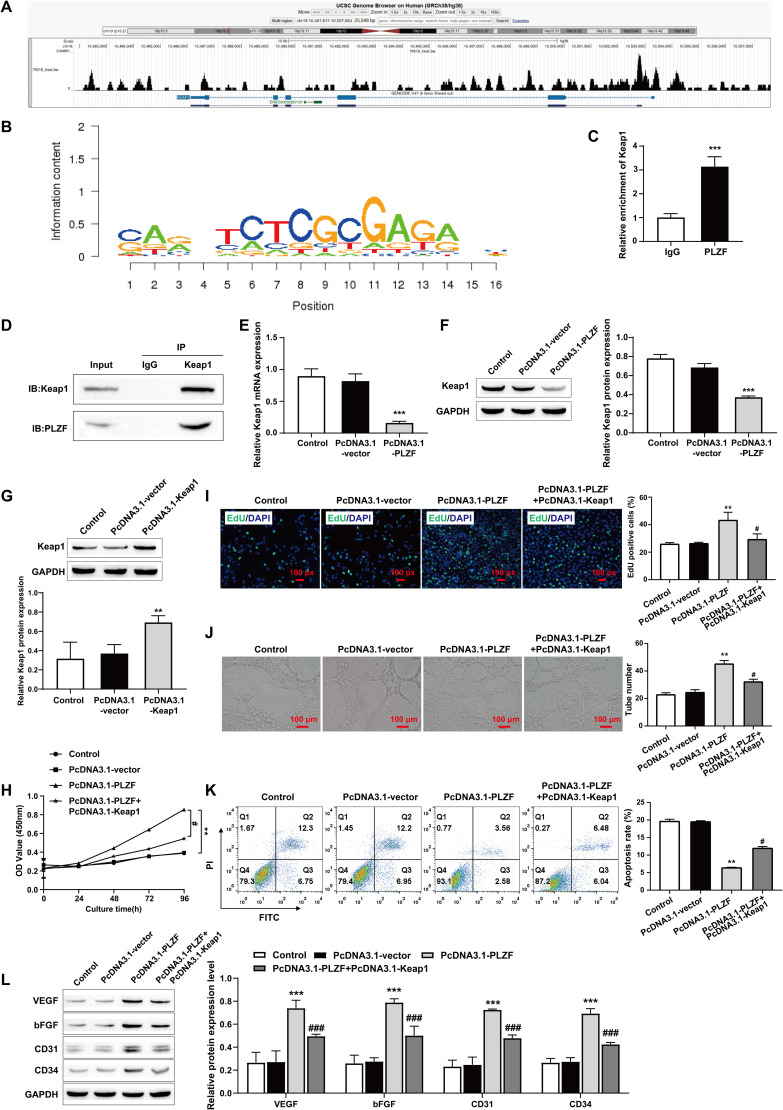
PLZF promotes HPMECs proliferation and angiogenesis by inhibiting Keap1. A, B: The Cistrome DB database predicted PLZF binding sites and binding sequences in Keap1 promoter; C: ChIP was used to detect the binding of PLZF to the promoter region of Keap1 gene; D: Coimmunoprecipitation was used to assess the interaction between PLZF and Keap1; E: RT‒qPCR was used to assess the expression level of Keap1 in HPMECs; F: Western blotting was used to assess the expression level of Keap1 in HPMECs; G: The transfection efficiency of PcDNA3.1‒Keap1 was assessed by Western blot; H: The proliferation ability of HPMECs was assessed by CCK-8; I: EdU staining was used to assess the proliferation activity of HPMECs and quantify the percentage of EdU-positive cells, scale bars: 100 px; J: A tube formation assay was used to assess the angiogenic ability of HPMECs, scale bars: 100 μm; K: The apoptosis of HPMECs was assessed by flow cytometry; L: Expression levels of the angiogenesis markers VEGF, bFGF, CD31, and CD34 were determined by Western blot. The data are expressed as the mean ± SD (*n* = 3). ^**^P < 0.01, ^***^P < 0.001 vs. Control; ^#^*P* < 0.05, ^###^*P* < 0.001 vs. PcDNA3.1-PLZF.

To further identify the pathway by which PLZF promotes HPMECs proliferation and angiogenesis and inhibits HPMECs apoptosis. Firstly, we found through the analysis of the Cistrome DB database (http://cistrome.org/db/) PLZF exist in Keap1 promoter region binding sites (Figs 3A and B). Therefore, we explored the effect of PLZF on Keap1 expression. ChIP results showed that PLZF was able to bind to the Keap1 promoter (Fig 3C). We then examined the interaction between PLZF and Keap1 by co-immunoprecipitation. The Western blot results revealed that PLZF and Keap1 were present in the samples after immunoprecipitation, suggesting an interaction between PLZF and Keap1 in HPMECs (Fig 3D). The RT-qPCR and Western blot results revealed that overexpression of PLZF inhibited Keap1 expression (Fig 3E, and F). HPMECs were then cotransfected with PLZF and Keap1 plasmids, and Western blot results revealed that Keap1 overexpression significantly increased the expression of Keap1 in HPMECs, indicating successful transfection (Fig 3G). Furthermore, the results of the CCK-8 and EdU experiments revealed that PLZF overexpression accelerated cell proliferation, whereas Keap1 overexpression reversed this process (Figs 3H and I). Tube formation experiments also revealed similar results, with Keap1 overexpression also reversing the PLZF-mediated promotion of angiogenesis (Fig 3J). Similarly, the flow cytometry results revealed that Keap1 overexpression significantly reversed the inhibitory effect of PLZF overexpression on HPMECs apoptosis (Fig 3K). In addition, the expression levels of angiogenesis markers measured by western blotting revealed that the expression levels of VEGF, bFGF, CD31, and CD34 were significantly increased in the PcDNA3.1-PLZF group, which was reversed by Keap1 overexpression (Fig 3L). Taken together, these results suggest that PLZF may promote the proliferation of HPMEC and angiogenesis by negatively regulating Keap1.

### 3.4. PLZF promotes angiogenesis without affecting HPMECs proliferation by activating the HIF-1α/VEGF signaling pathway

**Fig 4:**
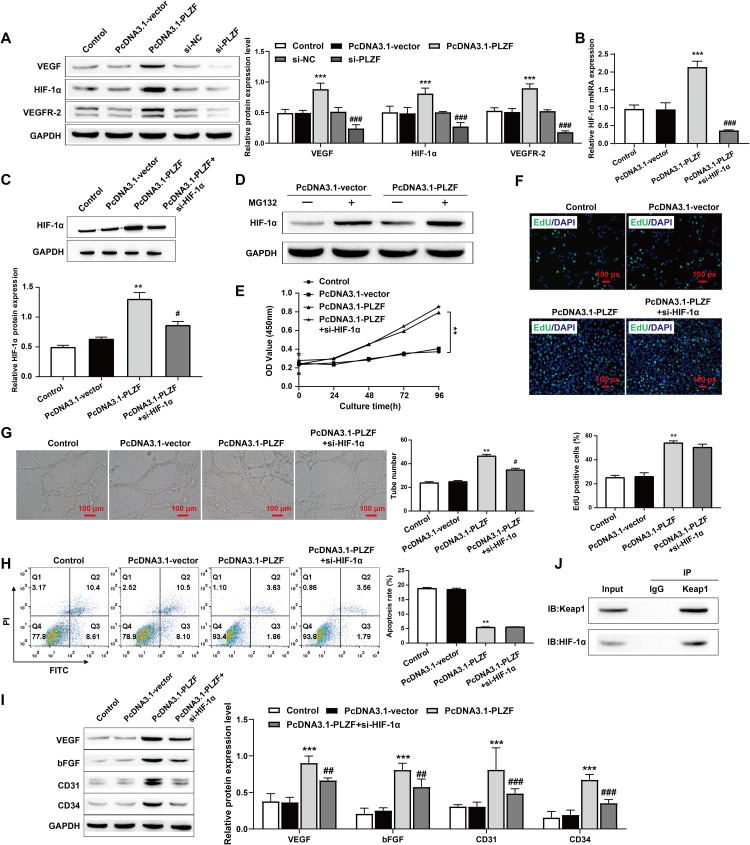
PLZF promotes angiogenesis without affecting HPMECs proliferation by activating the HIF-1 **α/VEGF signaling pathway**. A: Western blotting was used to assess the expression levels of VEGF, HIF-1α and VEGFR-2 in HPMECs; B: RT‒qPCR was used to assess the expression level of HIF-1α; C: The expression level of HIF-1α was assessed by Western blotting; D: The cells were treated with the proteasome inhibitor MG132, and the expression of HIF-1α was detected by Western Blot; E: CCK-8 was used to assess the proliferation ability of HPMECs; F: HPMECs proliferation was assessed by EdU staining and the percentage of EdU-positive cells was quantified, scale bars: 100 px; G: A tube formation assay was used to assess the angiogenic ability of HPMECs, scale bars: 100 μm; H: HPMECs apoptosis was assessed by flow cytometry; I: Western blotting was used to assess the expression levels of the angiogenesis markers VEGF, bFGF, CD31, and CD34; J: Co-immunoprecipitation was used to detect the interaction between Keap1 and HIF-1α. The data are expressed as the mean ± SD (*n *= 3). ^**^ *P* < 0.01, ^***^ *P* < 0.001 vs. Control; ^#^*P* < 0.05, ^##^*P* < 0.01, ^###^*P* < 0.001 vs. PcDNA3.1-PLZF.

Previous studies have shown that the HIF-1α/VEGF signaling pathway plays an important role in angiogenesis, so we investigated the regulation of the HIF-1α/VEGF signaling pathway by PLZF [27,28]. First, PLZF was overexpressed or knocked down, and the expression of downstream signals was assessed by western blotting. Compared with those in the control group, the protein expression levels of VEGF, HIF-1α, and VEGFR-2 were significantly increased when PLZF was overexpressed and significantly decreased when PLZF was knocked down (Fig 4A). Then, we simultaneously transfected HPMECs with the PLZF overexpression plasmid and siRNA targeting HIF-1α. The results revealed that the HIF-1α level significantly decreased after simultaneous transfection (Figs 4B and C). After treatment of cells with protease inhibitor MG132, we found that HIF-1α moderately accumulated in PcDNA3.1-vector+MG132 group cells, and overexpression of PLZF further promoted HIF-1α protein levels, indicating that PLZF overexpression may stabilize HIF-1α protein by inhibiting the proteasome pathway (Fig 4D). Furthermore, CCK-8 and EdU assays revealed that overexpression of PLZF promoted the proliferation of HPMECs, whereas knockdown of HIF-1α had no significant effect on the proliferation of HPMECs (Figs 4E and F). Notably, PLZF overexpression promoted HPMECs angiogenesis, and HIF-1α knockdown inhibited the effect of PLZF overexpression on HPMECs angiogenesis (Fig 4G). In addition, the flow cytometry results also revealed that PLZF overexpression inhibited HPMECs apoptosis, whereas HIF-1α knockdown had no significant effect on HPMECs apoptosis (Fig 4H). Moreover, the Western blot results revealed that the expression levels of the angiogenesis markers VEGF, bFGF, CD31, and CD34 in the PcDNA3.1-PLZF group were significantly increased, and HIF-1α knockout inhibited the expression of angiogenesis markers (Fig 4I). Finally, We detected whether Keap1 and HIF-1α had an interaction relationship through co-immunoprecipitation. The Western Blot detection results showed that Keap1 and HIF-1α could interact in HPMECs cells. (Fig 4J). In summary, we made the interesting observation that HIF-1α knockdown inhibited only the promoting effect of PLZF overexpression on angiogenesis in HPMECs and had no effect on their proliferation or apoptosis. PLZF may promote HPMECs angiogenesis by inhibiting Keap1 activation of the HIF-1α/VEGF signaling pathway but has no effect on cell proliferation or apoptosis.

### 3.5. PLZF promotes HPMECs proliferation and angiogenesis via activation of the Nrf2 signaling pathway via HIF-1α/VEGF

**Fig 5:**
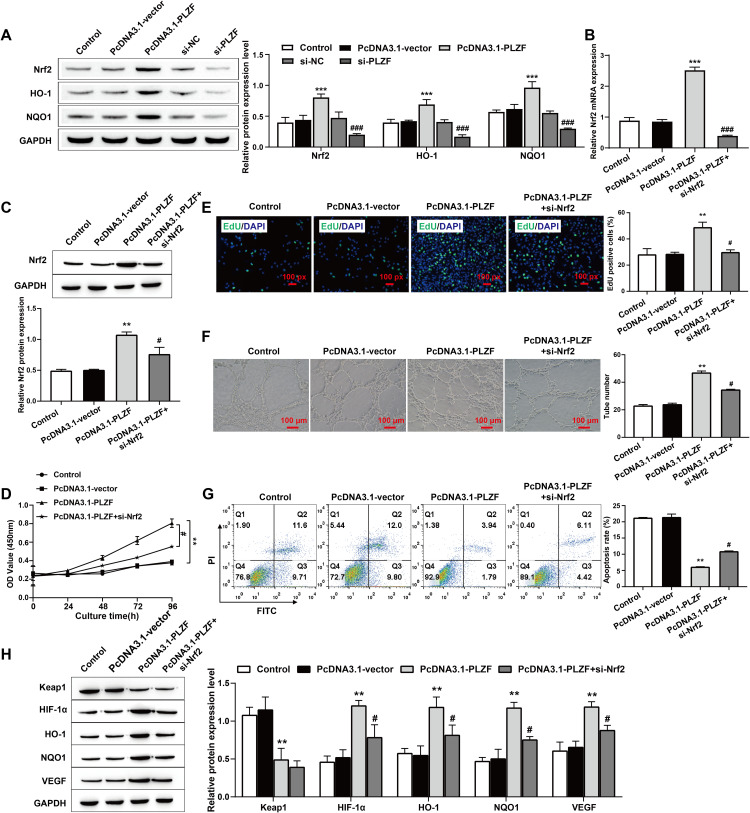
PLZF promotes HPMECs proliferation and angiogenesis via activation of the Nrf2 signaling pathway-mediated HIF-1 **α/VEGF**. A: Western blotting was used to assess the expression levels of Nrf2, HO-1 and NQO1 in HPMECs; B: The expression level of Nrf2 was assessed via RT‒qPCR; C: Western blotting was used to assess the expression level of Nrf2; D: CCK‒8 was used to assess the proliferation ability of HPMECs; E: EdU staining was used to assess the proliferation activity of HPMECs and quantify the percentage of EdU-positive cells, scale bars: 100 px; F: A tube formation assay was used to assess the angiogenic ability of HPMECs, scale bars: 100 μm; G: HPMECs apoptosis was assessed by flow cytometry; H: Western blotting was used to assess the expression levels of Keap1, HIF-1α, HO-1, NQO1, and VEGF in HPMECs. The data are expressed as the mean ± SD (*n *= 3). ^**^ *P* < 0.01, ^***^ *P* < 0.001 vs. Control; ^#^*P* < 0.05, ^##^*P* < 0.01, ^###^*P* < 0.001 vs. PcDNA3.1-PLZF.

To further explore the mechanism underlying the above results, we next overexpressed or knocked down PLZF, and western blotting was used to assess the expression of downstream signals. Compared with those in the control group, the expression levels of Nrf2, HO-1, and NQO1 in the cells were significantly increased when PLZF was overexpressed and significantly decreased when PLZF was knocked down (Fig 5A). Next, we simultaneously transfected HPMECs with the PLZF overexpression plasmid and Nrf2-targeted siRNA. The results of RT‒qPCR and Western blotting revealed that the levels of Nrf2 showed a significant downward trend after simultaneous transfection (Figs 5B and C). Furthermore, CCK-8 and EdU assays revealed that the proliferation ability of HPMECs was significantly increased after PLZF overexpression and decreased after Nrf2 knockdown (Figs 5D and E). The results of the tube formation assay revealed that the vessel formation ability of HPMECs in the PcDNA3.1-PLZF group was significantly greater than that in the PcDNA3.1-vector group, and the vessel formation ability of HPMECs in the PcDNA3.1-PLZF+si-Nrf2 group was significantly lower than that in the PcDNA3.1-PLZF group (Fig 5F). The flow cytometry results revealed that the percentage of apoptotic HPMECs was significantly greater in the PcDNA3.1-PLZF+si-Nrf2 group than in the PcDNA3.1-PLZF group (Fig 5G). Compared with the PcDNA3.1-PLZF group, the PcDNA3.1-PLZF +si-Nrf2 group exhibited significant reductions in the protein expression of HO-1, NQO1, HIF-1α, and VEGF but no significant difference in the protein expression of Keap1 (Fig 5H). Taken together, our results suggest that Nrf2 knockdown not only affects HPMECs proliferation and apoptosis but also affects their angiogenesis. The possible mechanism is that PLZF promotes HPMECs proliferation and inhibits apoptosis by inhibiting Keap1-activated Nrf2 to promote the transcription and expression of its target genes HO-1 and NQO1, and activates HIF-1α/VEGF signaling pathway to promote HPMECs angiogenesis.

### 3.6. PLZF overexpression promotes CLG after PNX in mice

**Fig 6:**
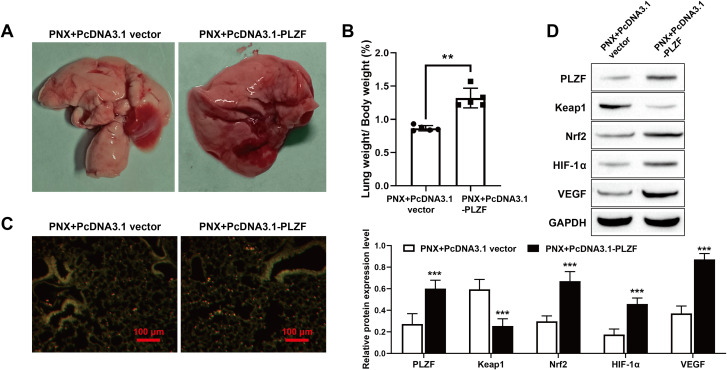
PLZF overexpression promotes CLG after PNX in mice. A: Representative image of the remaining lung; B: Ratio of lung weight to body weight; C: Immunofluorescence was used to assess the proliferation of pulmonary endothelial cells, scale bars: 100 μm; D: Western blot analysis of the levels of PLZF, Keap1, Nrf2, HIF-1α and VEGF in lung tissue. The data are expressed as the mean ± SD (*n* = 5). ** *P* < 0.01, *** *P* < 0.01 vs. PNX + PcDNA3.1 vector.

We constructed a unilateral PNX mouse model and injected the PLZF overexpression chronic disease vector into mice by intraperitoneal injection to observe the role of PLZF in promoting CLG. Compared with the PNX + PcDNA3.1 vector, PLZF overexpression significantly increased the residual lung volume (Fig 6A). Furthermore, PLZF overexpression significantly increased the ratio of the lung weight to the body weight of the mice (Fig 6B). Furthermore, to assess the effect of PLZF overexpression on pulmonary endothelial cells, pulmonary endothelial cells were subjected to staining for visualization. The results showed that PLZF overexpression significantly promoted endothelial cell proliferation (Fig 6C). Moreover, the Western blot results revealed that PLZF overexpression significantly increased the protein expression levels of PLZF, Keap1, Nrf2, HIF-1α, and VEGF, and decreased the expression level of Keap1 in the lung tissue (Fig 6D). In conclusion, our in vivo results indicate that PLZF overexpression significantly increases the volume and weight of the residual lung after unilateral PNX and the proliferation of lung endothelial cells.

## 4. Discussion

The metabolism of the human body, or the development of organs and wound healing, is accompanied by countless cells being generated and undergoing apoptosis because the body is in a stable state, ensuring healthy development. ECs are among these cells [32]. In addition to these functions, ECs have a specific function in angiogenesis [33]. There is also some evidence indicating that EC also actively promotes the development of CLG and alveolar morphology [34]. An analysis of the data from this study revealed that the PLZF gene showed a clear downward trend after unilateral PNX. Subsequently, we confirmed in vitro that overexpression of PLZF could promote the proliferation and angiogenesis of HPMEC and inhibit their apoptosis. It has also been confirmed in vivo that overexpression of PLZF promotes the remaining lung volume and weight after unilateral PNX in mice, and significantly promotes the proliferation of pulmonary endothelial cells. This effect may be mediated by the inhibition of Keap1 activation of Nrf2 and HIF-1α/VEGF signaling.

PLZF is a multifunctional transcription factor involved in a wide range of biological processes, including stem cell self-renewal, differentiation, immune cell function regulation and angiogenesis. In the tumor microenvironment, angiogenesis is a key factor for tumor growth and metastasis, and PLZF may indirectly affect tumor angiogenesis by regulating the HIF-1α/VEGF signaling pathway [35,36]. Activation of PLZF protects endothelial cells from advanced glycation end product (AGE)-induced endothelial cell damage in diabetes-related vascular complications [35]. The present study further confirmed that overexpression of PLZF promoted HPMECs proliferation and angiogenesis. In vivo, PLZF overexpression significantly increased the volume and weight of the remaining lung after unilateral PNX and promoted the proliferation of pulmonary endothelial cells. PLZF may promote HPMECs proliferation and inhibit apoptosis by inhibiting Keap1-activated Nrf2 in vitro. At the same time, the HIF-1α/VEGF signaling pathway is activated to promote HPMECs angiogenesis.

When the body recovers from serious injury, the repair of organs cannot be separated from the early regeneration of the vascular system, which is complex and extremely important [37]. Many literature reviews have revealed that the HIF-1α/VEGF signaling pathway has a unique and important function in tumor angiogenesis, but triggering CLG also requires angiogenesis to increase the alveolar number and alveolar density, thereby enhancing residual lung function. HIF-1α, a master regulator of hypoxia, binds to HREs in the promoter region of VEGF, a key driver of angiogenesis [38]. VEGF is a direct target of HIF-1α. Deficiency of VEGF results in reduced lung maturity, decreased surfactant production, and vascular and alveolar hypoplasia [39]. Exogenous VEGF has been shown to accelerate CLG [29]. In this study, overexpression of PLZF in HPMECs promoted cell proliferation and angiogenesis, inhibited apoptosis, and significantly increased HIF-1α and VEGF protein expression. Interestingly, knockdown of HIF-1α inhibited the effect of PLZF overexpression on HPMECs angiogenesis but had no effect on HPMECs proliferation or apoptosis.

The Keap1‒Nrf2 pathway is a major protective response to oxidative and electrophilic stress [40]. Keap1 is a natural inhibitor of Nrf2. The inhibition of Keap1 can activate Nrf2 and transfer it to the nucleus [41]. A literature review revealed that Nrf2 can promote angiogenesis. We also know from the literature that the level of Nrf2 tends to increase during the process of vascular development and that its decreased level inhibits angiogenesis germination and reduces vascular density [42]. Nrf2 knockdown significantly reduces HO-1 and VEGF expression [43]. Nrf2 can regulate angiogenesis through the HO-1-mediated HIF-1α/VEGF signaling pathway [44]. In the present study, overexpression of Keap1 significantly reversed the promoting effects of PLZF overexpression on HPMECs proliferation and angiogenesis and increased their degree of apoptosis. Knockdown of Nrf2 significantly inhibited the proliferation and angiogenesis of HPMECs induced by PLZF overexpression and increased the level of cell apoptosis. Moreover, the protein expression of Nrf2, HO-1, NQO1, HIF-1α and VEGF was significantly decreased, whereas the protein expression of Keap1 was not affected.

However, in addition to the above mechanisms, PLZF may affect angiogenesis through other pathways. For example, Nrf2 is able to regulate the expression of a variety of antioxidant enzymes, such as HO-1 and NQO1, which are not only involved in antioxidant reactions but may also affect angiogenesis by regulating the intracellular redox balance [45]. In addition, HIF-1α is able to upregulate a variety of angiogenic-related factors, such as PDGF, FGF, and ANG-1, which play important roles in the early stages of angiogenesis [46]. Thus, PLZF may act synergistically through multiple mechanisms to promote angiogenesis and compensatory lung growth. In future studies, we will further explore the specific mechanisms of PLZF in these pathways to fully understand its potential in angiogenesis and compensatory lung growth.

Taken together, our findings suggest that PLZF promotes HPMECs proliferation and angiogenesis and promotes CLG by inhibiting Keap1 activation of the Nrf2 and HIF-1α/VEGF signaling pathways. Our study provides a new research strategy for specific genes regulating EC function to promote CLG.

## Supporting information

S1 File Raw images. (PDF)

S2 Fig Graphical abstract. Potential mechanisms of PLZF regulation of HPMECs proliferation, apoptosis, and angiogenesis.
(JPG)

S3 File Article polishing report. (PDF)

S4 File Raw data 1. (ZIP)

S5 File Raw data 2. (ZIP)

S6 File Raw data 3. (ZIP)

S7 File Raw data 4. (ZIP)

S8 File Raw data 5. (ZIP)
