# Supplementary material for: PLZF promotes compensatory lung growth by increasing HPMEC proliferation and angiogenesis
Source: PLoS One. 2025 Jul 2;20(7):e0325936. doi: 10.1371/journal.pone.0325936 (PMC12221005; doi:10.1371/journal.pone.0325936)

Figure 1D

PLZF (74KD)

Repeat 1 Repeat 2 Repeat 3

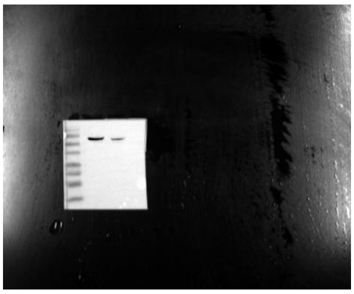
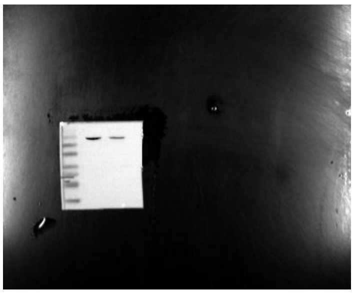
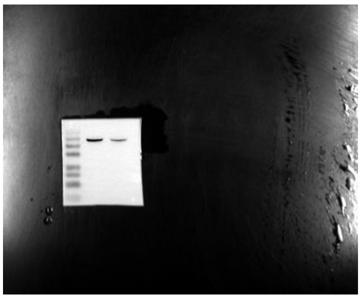

Repeat 4 Repeat 5

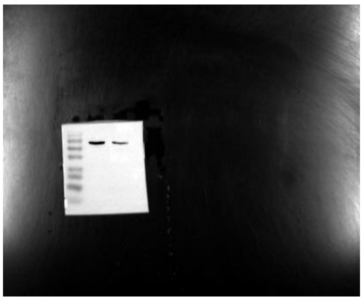
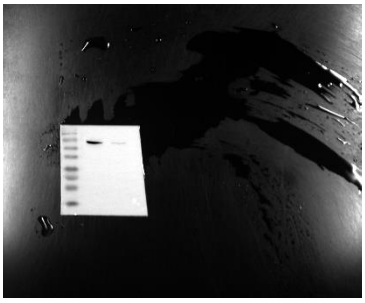

GAPDH (36KD)

Repeat 1 Repeat 2 Repeat 3

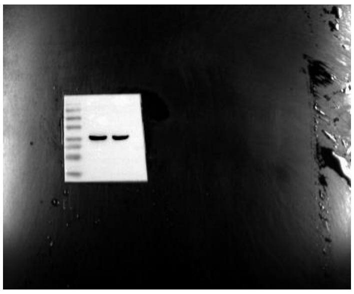
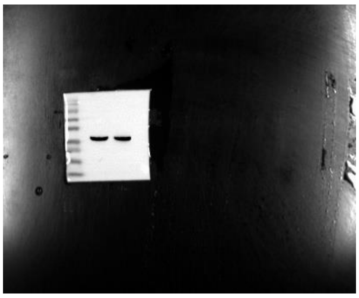
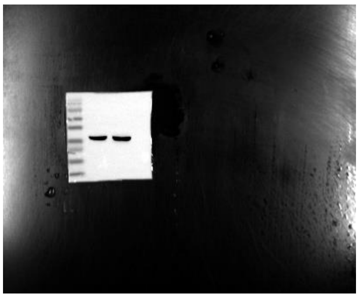

Repeat 4 Repeat 5

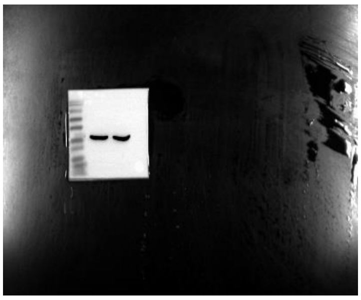
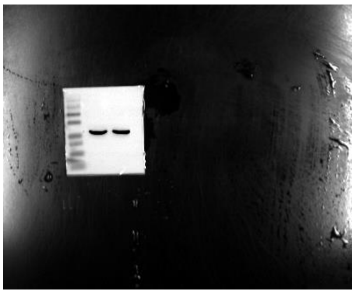

Figure 2A

PLZF (74KD)

Repeat 1 Repeat 2 Repeat 3

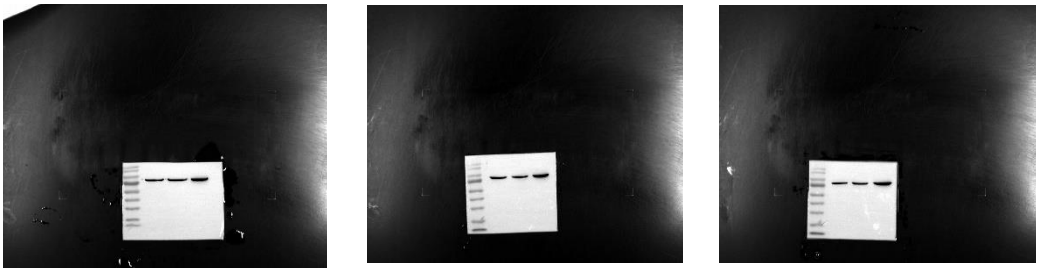

GAPDH (36KD)

Repeat 1 Repeat 2 Repeat 3

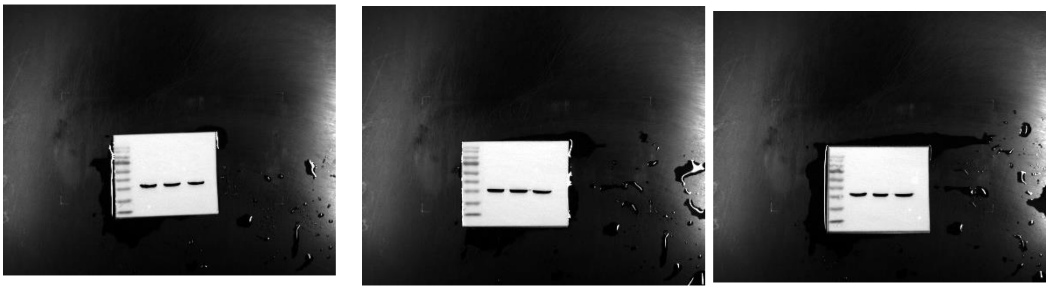

Figure 2F

VEGF (180KD)

Repeat 1 Repeat 2 Repeat 3

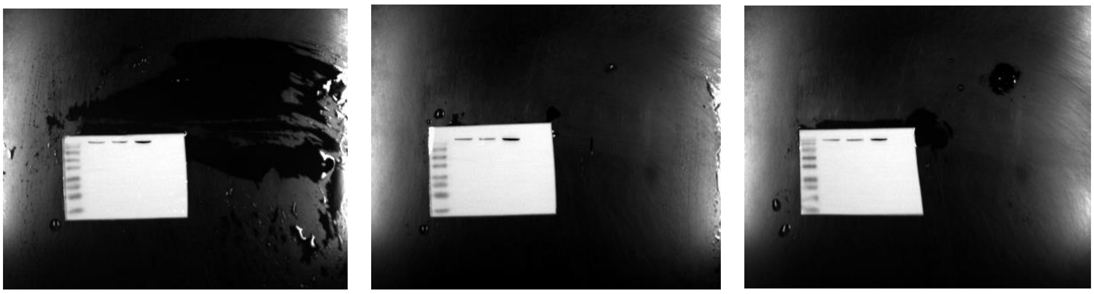

bFGF (19KD)

Repeat 1 Repeat 2 Repeat 3

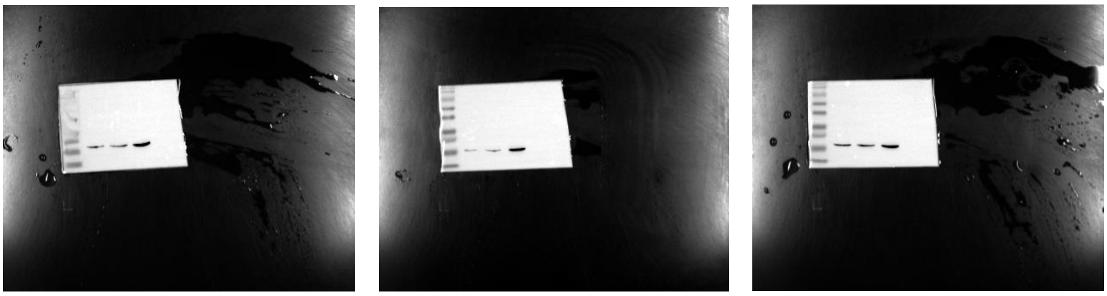

CD31(125KD)

Repeat 1 Repeat 2 Repeat 3

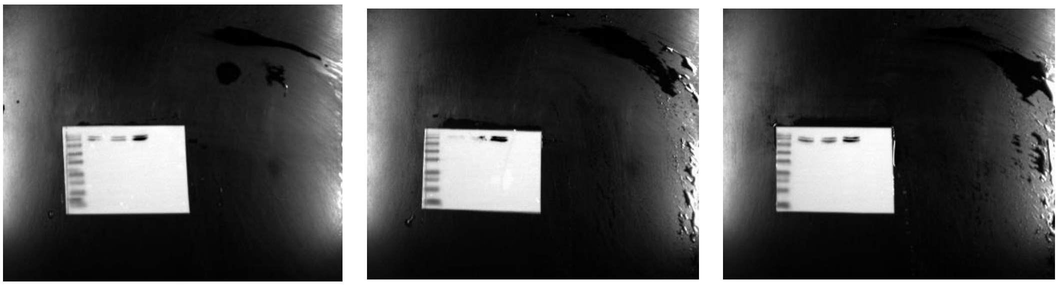

CD34 (100KD)

Repeat 1 Repeat 2 Repeat 3

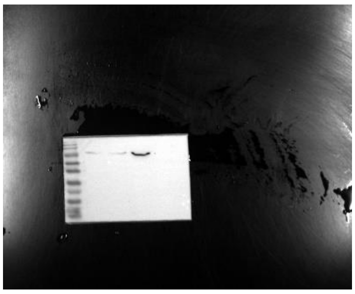
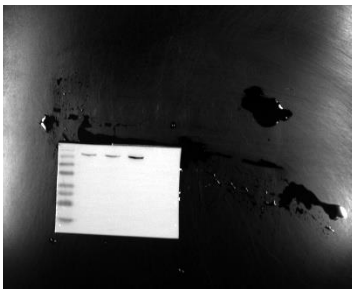
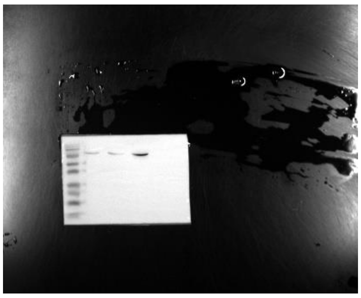

GAPDH (36KD)

Repeat 1 Repeat 2 Repeat 3

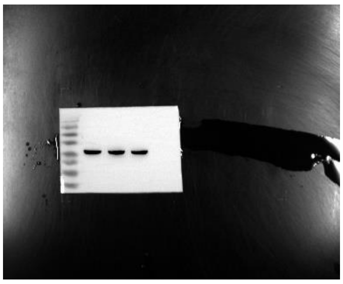
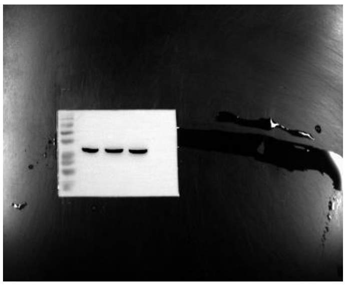
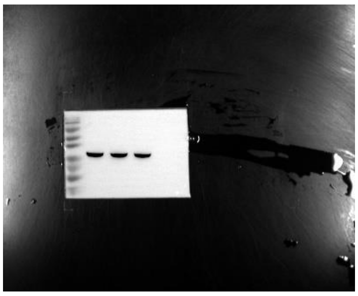

Figure 3A

Keap1 (70KD)

Repeat 1 Repeat 2 Repeat 3

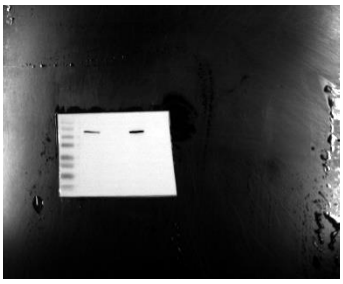
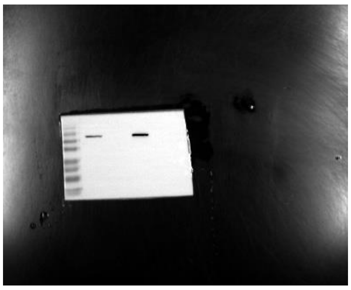
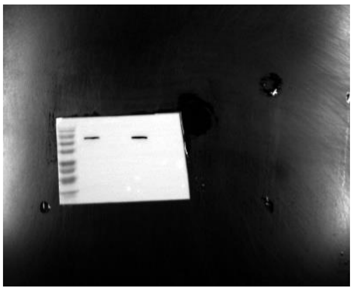

PLZF (74KD)

Repeat 1 Repeat 2 Repeat 3

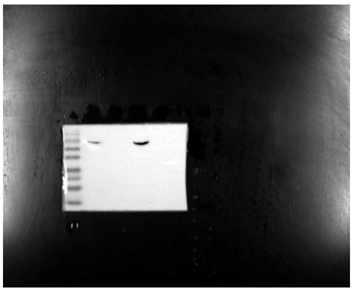
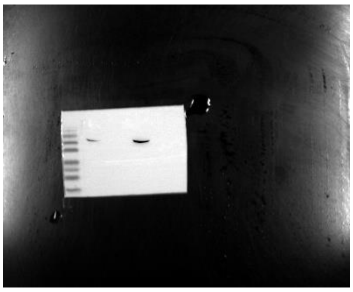
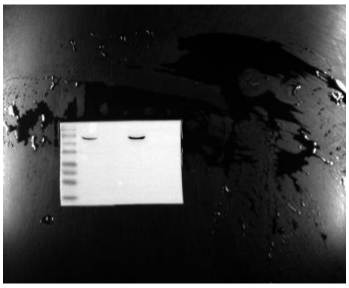

Figure 3B

Keap1 (70KD)

Repeat 1 Repeat 2 Repeat 3

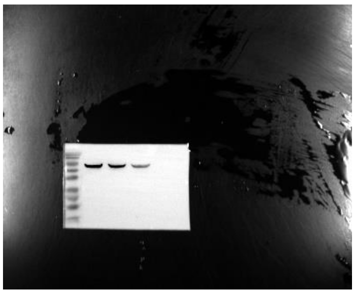
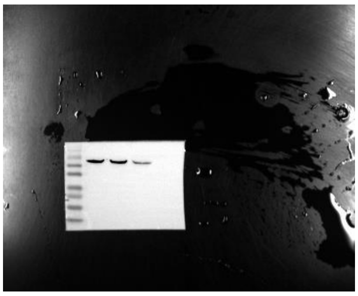
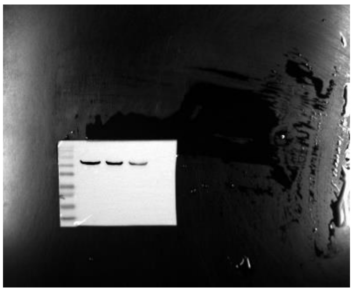

GAPDH (36KD)

Repeat 1 Repeat 2 Repeat 3

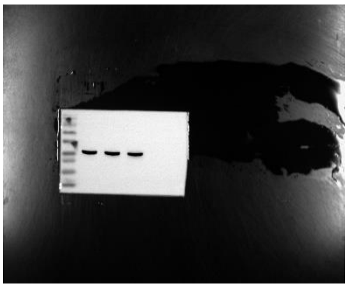
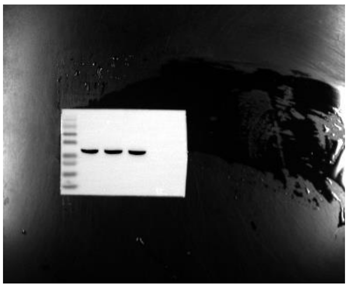
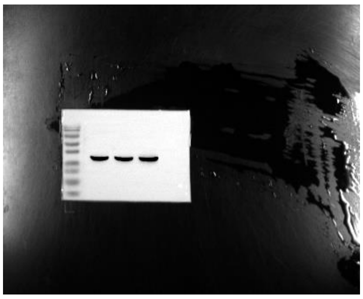

Figure 3D

Keap1 (70KD)

Repeat 1 Repeat 2 Repeat 3

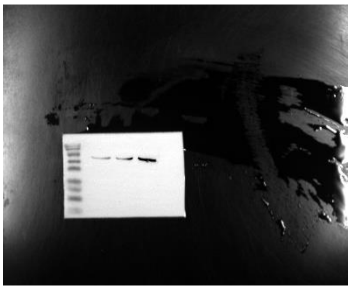
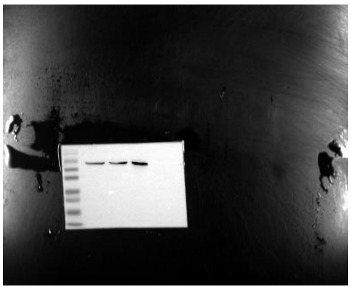
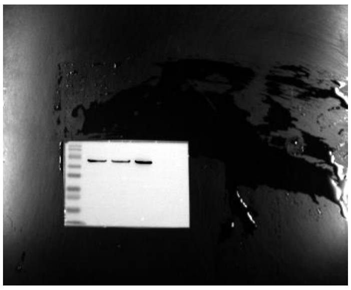

GAPDH (36KD)

Repeat 1 Repeat 2 Repeat 3

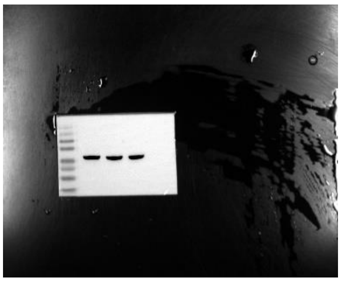
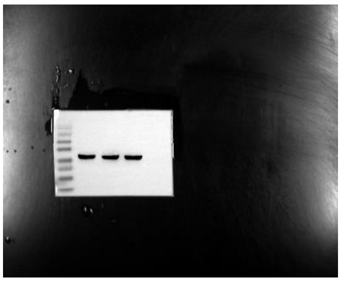
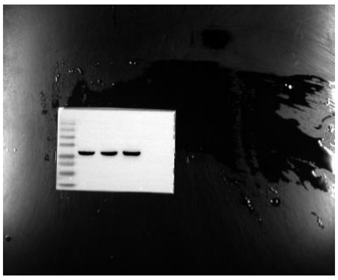

Figure3I

VEGF (180KD)

Repeat 1 Repeat 2 Repeat 3

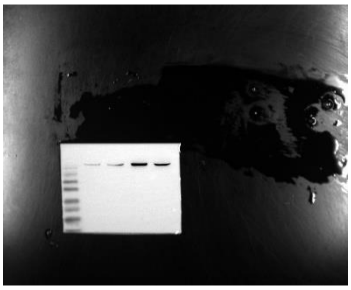
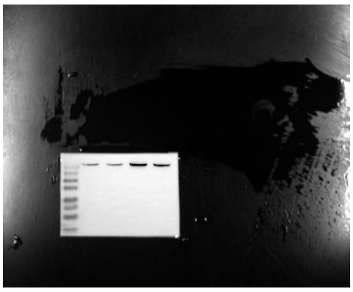
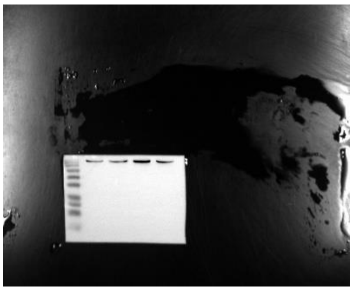

bFGF (19KD)

Repeat 1 Repeat 2 Repeat 3

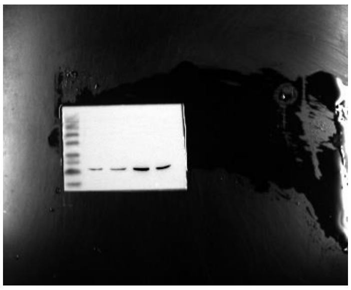
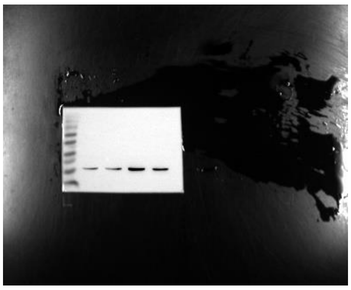
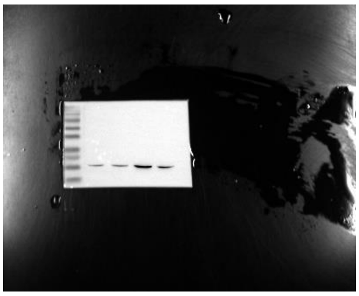

CD31(125KD)

Repeat 1 Repeat 2 Repeat 3

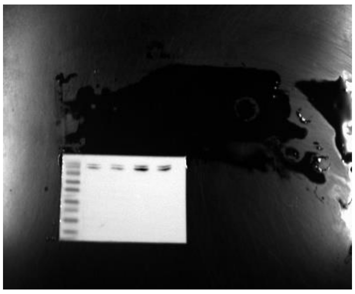
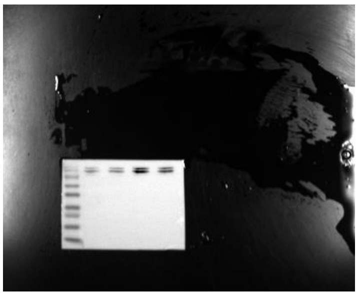
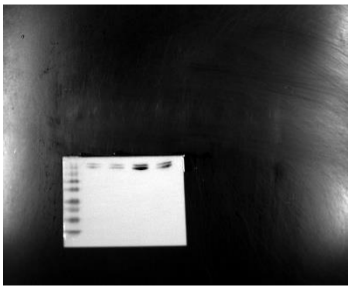

CD34 (100KD)

Repeat 1 Repeat 2 Repeat 3

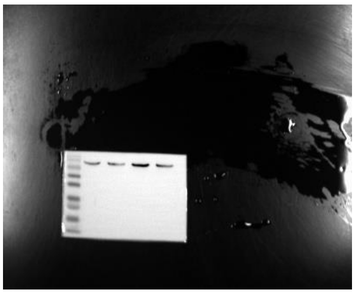
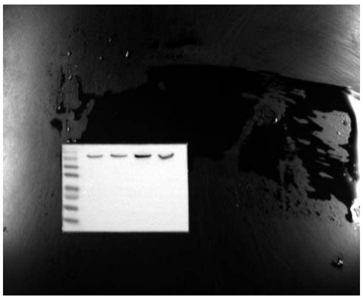
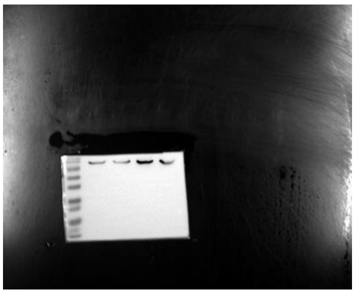

GAPDH (36KD)

Repeat 1 Repeat 2 Repeat 3

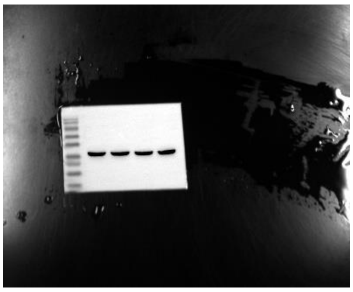
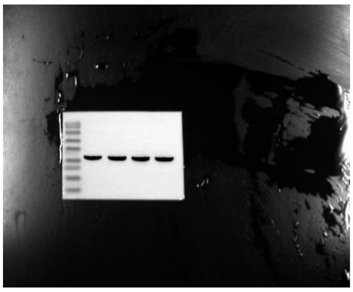
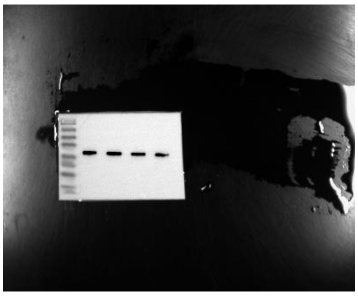

Figure4A

VEGF (180KD)

Repeat 1 Repeat 2 Repeat 3

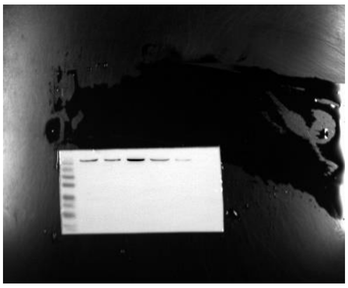
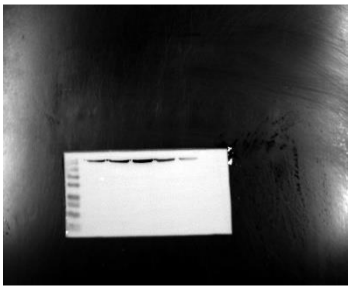
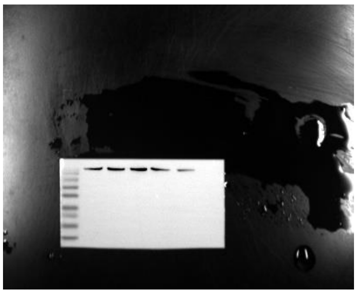

HIF-1α (92KD)

Repeat 1 Repeat 2 Repeat 3

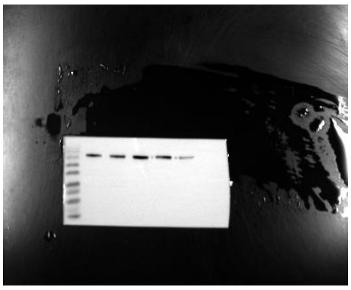
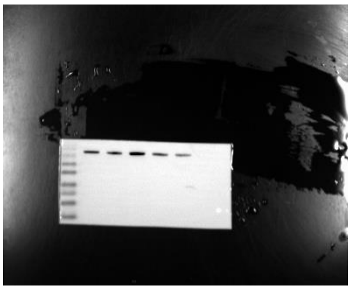
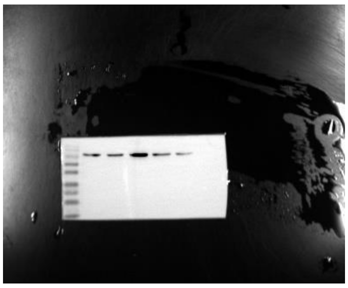

VEGFR-2 (230 kDa/ 200 kDa)

Repeat 1 Repeat 2 Repeat 3

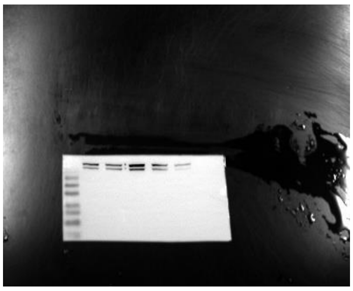
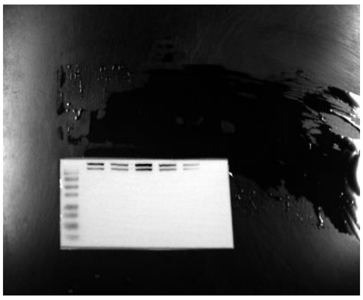
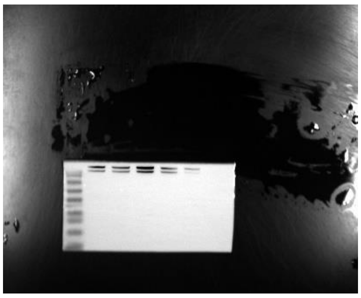

GAPDH (36KD)

Repeat 1 Repeat 2 Repeat 3

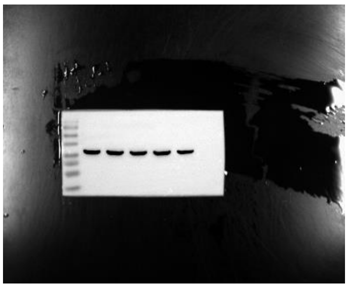
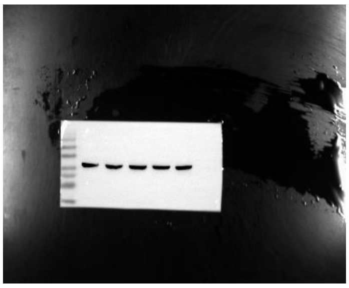
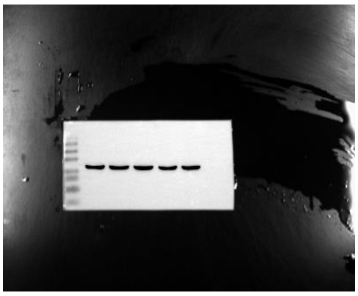

Figure 4C

Keap1 (70KD)

Repeat 1 Repeat 2 Repeat 3

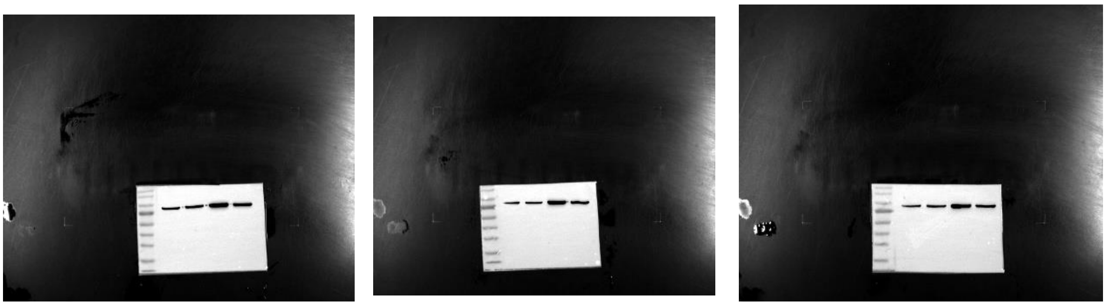

GAPDH (36KD)

Repeat 1 Repeat 2 Repeat 3

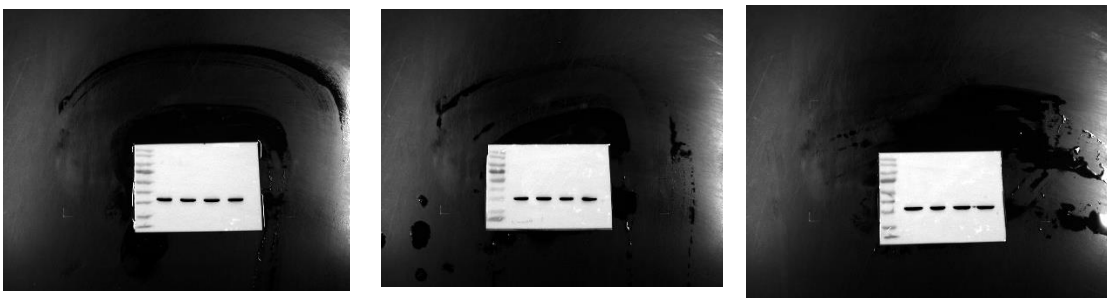

Figure4H

VEGF (180KD)

Repeat 1 Repeat 2 Repeat 3

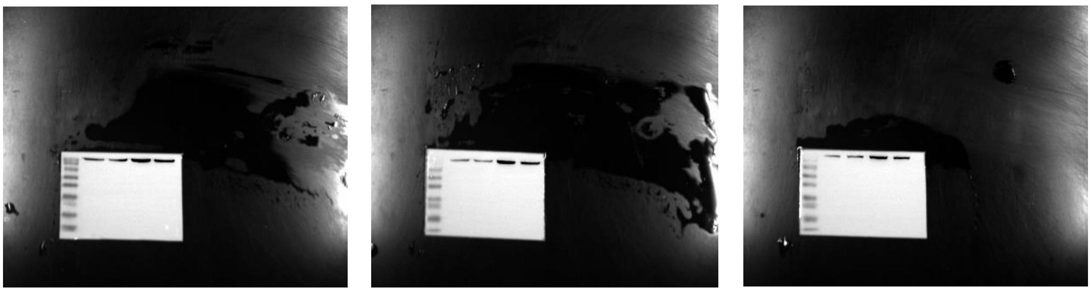

bFGF (19KD)

Repeat 1 Repeat 2 Repeat 3

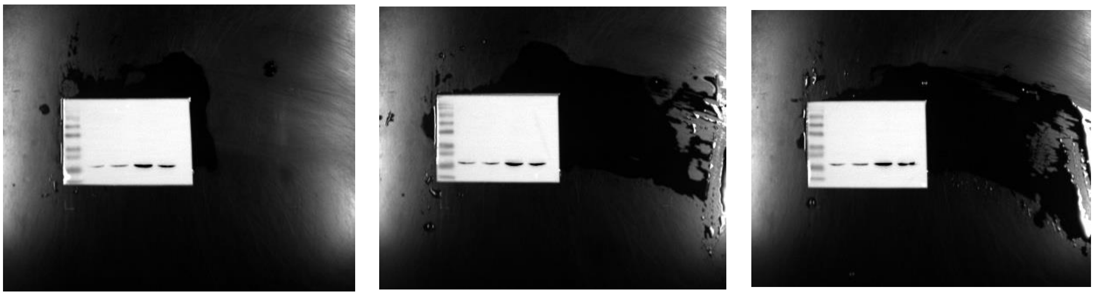

CD31(125KD)

Repeat 1 Repeat 2 Repeat 3

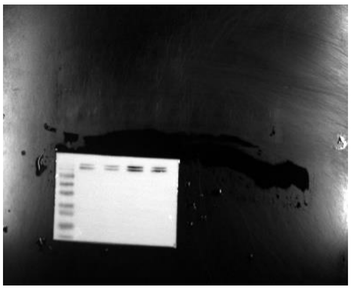
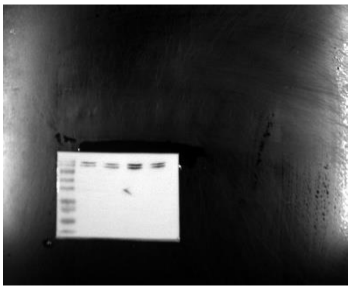
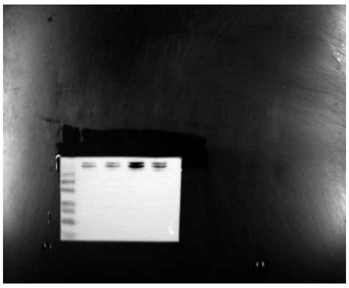

CD34 (100KD)

Repeat 1 Repeat 2 Repeat 3

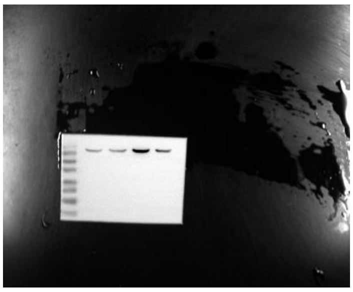
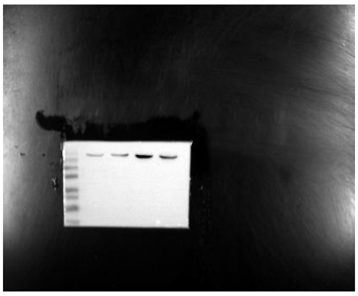
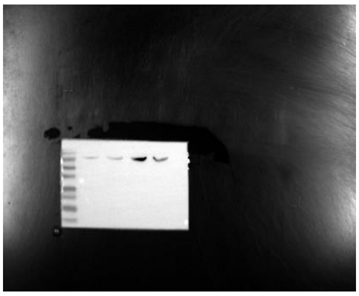

GAPDH (36KD)

Repeat 1 Repeat 2 Repeat 3

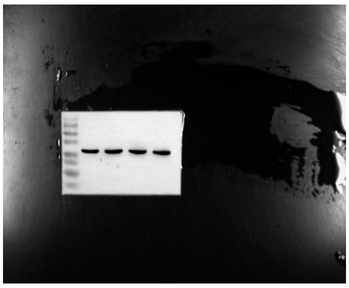
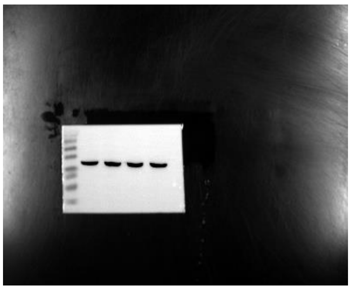
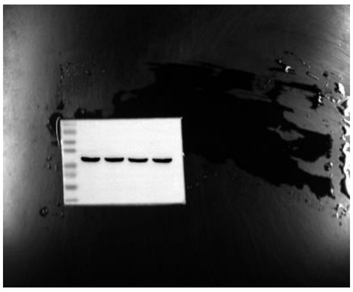

Figure5A

Nrf2 (110KD)

Repeat 1 Repeat 2 Repeat 3

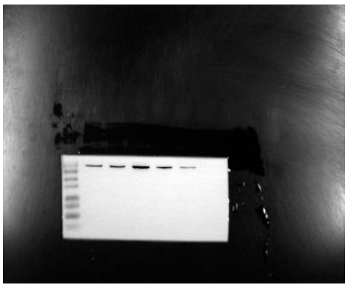
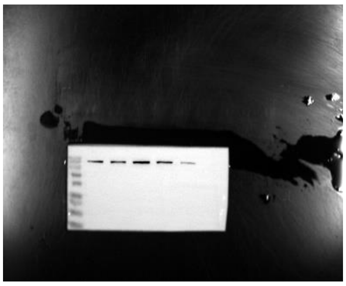
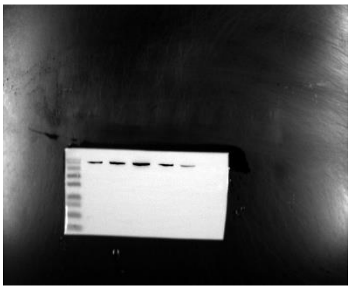

HO-1 (34KD)

Repeat 1 Repeat 2 Repeat 3

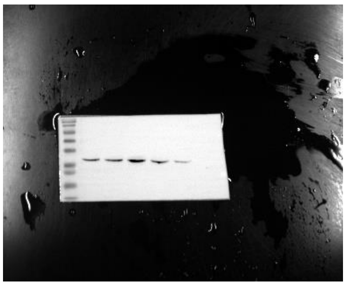
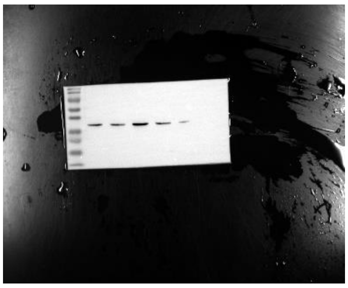
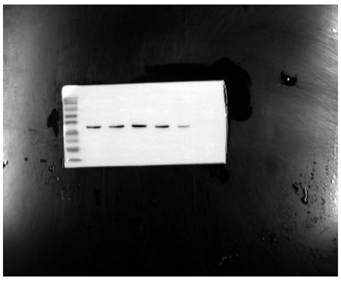

NQO1 (31 kDa)

Repeat 1 Repeat 2 Repeat 3

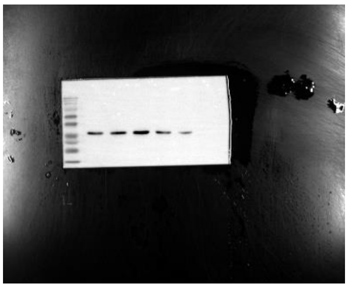
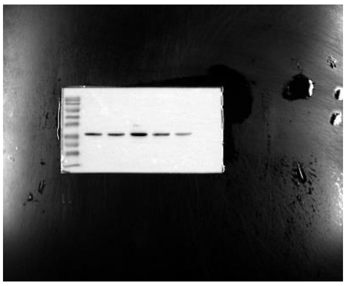
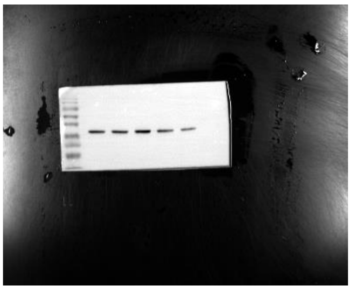

GAPDH (36KD)

Repeat 1 Repeat 2 Repeat 3

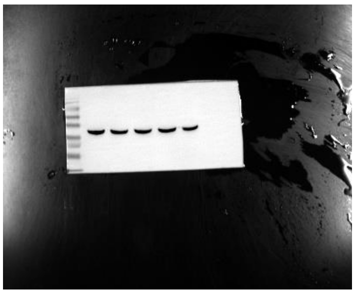
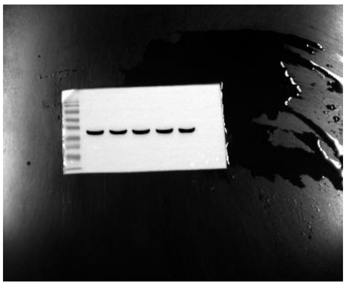
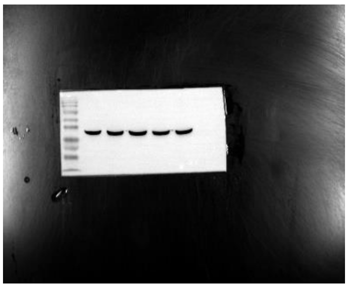

Figure5C

Nrf2 (110KD)

Repeat 1 Repeat 2 Repeat 3

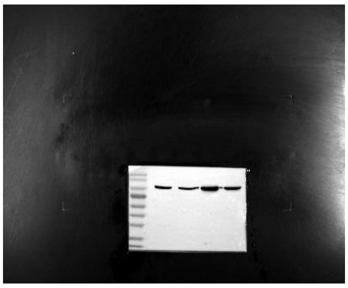
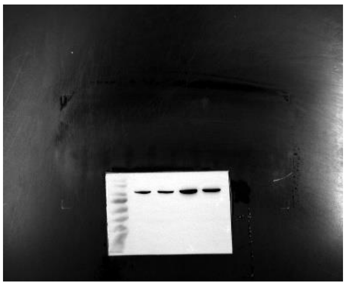
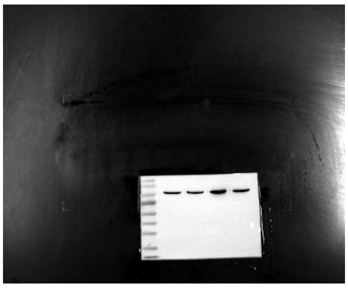

GAPDH (36KD)

Repeat 1 Repeat 2 Repeat 3

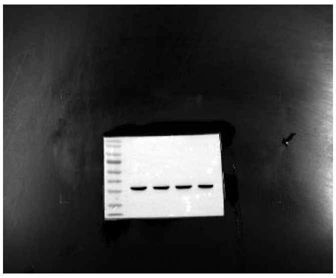
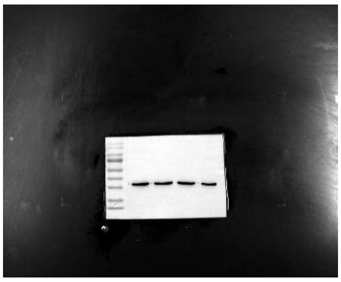
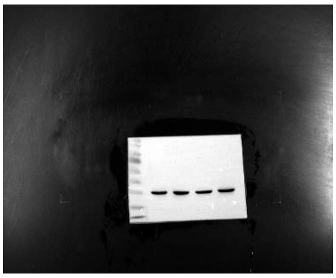

Figure5H

Keap1 (70 kDa)

Repeat 1 Repeat 2 Repeat 3

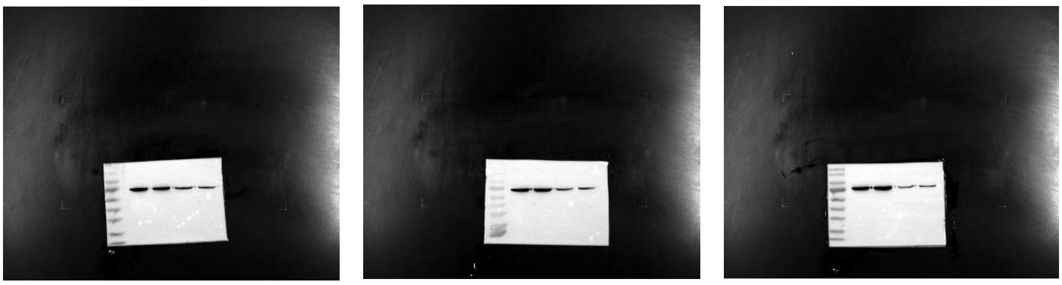

HIF-1α (92 kDa)

Repeat 1 Repeat 2 Repeat 3

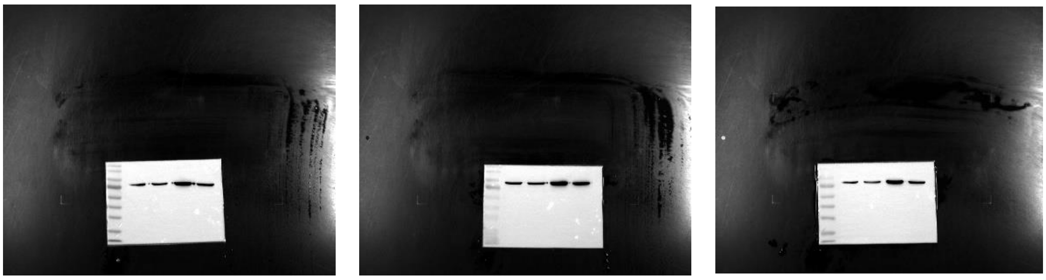

HO-1 (34KD)

Repeat 1 Repeat 2 Repeat 3

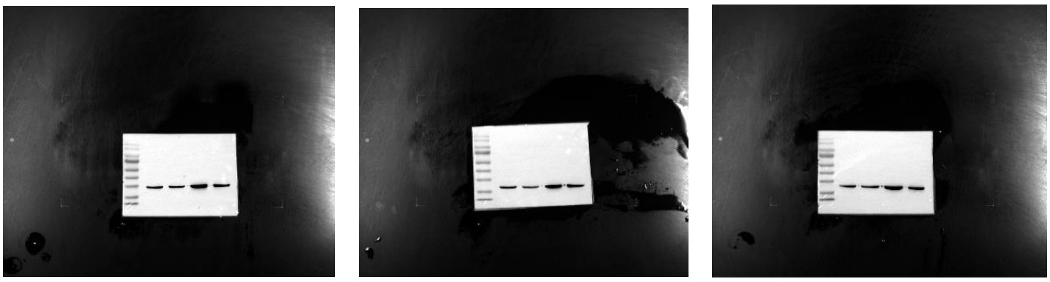

NQO1 (31 kDa)

Repeat 1 Repeat 2 Repeat 3

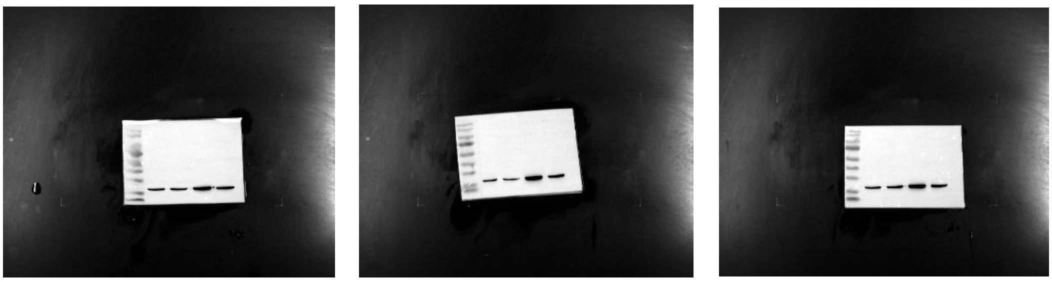

VEGF (180 kDa)

Repeat 1 Repeat 2 Repeat 3

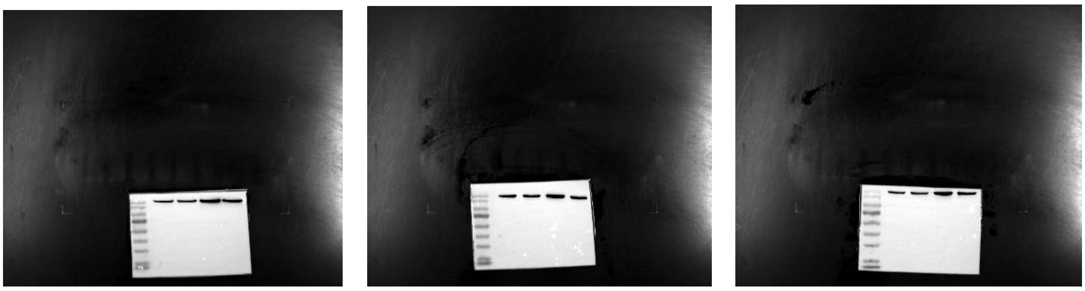

GAPDH (36KD)

Repeat 1 Repeat 2 Repeat 3

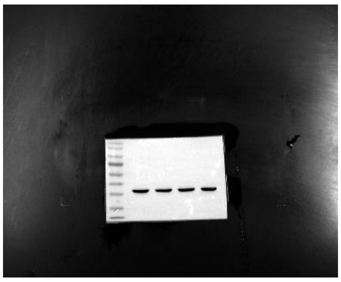
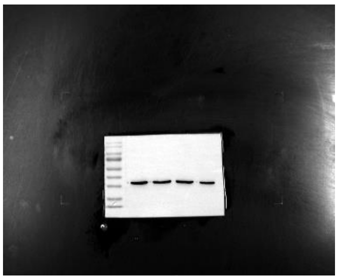
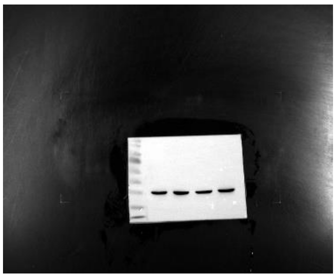

Figure6D

PLZF (74 kDa)

Repeat 1 Repeat 2 Repeat 3

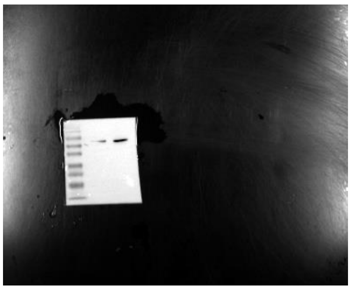
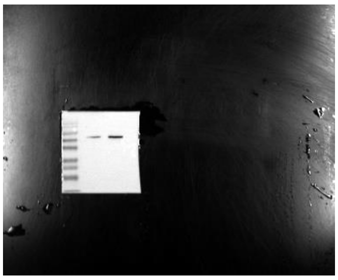
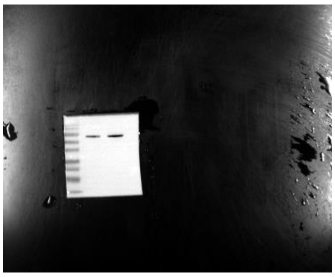

Repeat 4 Repeat 5

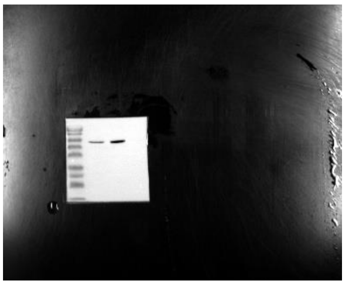
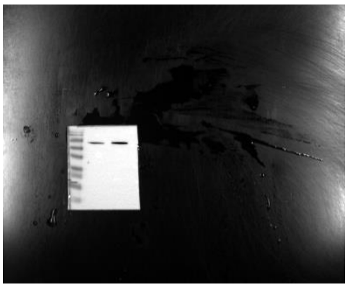

Keap1 (70 kDa)

Repeat 1 Repeat 2 Repeat 3

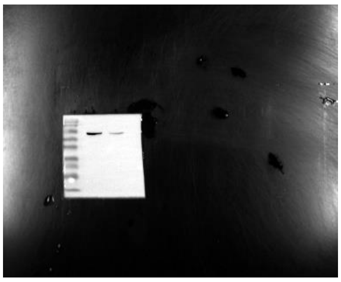
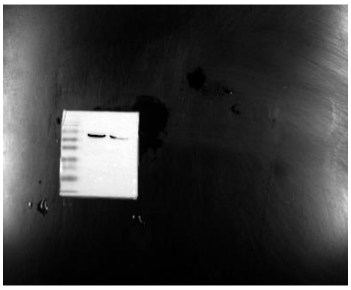
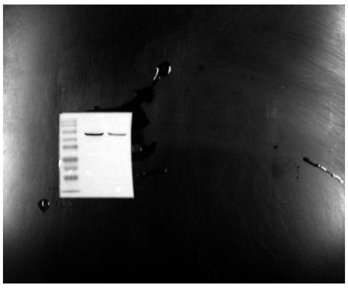

Repeat 4 Repeat 5

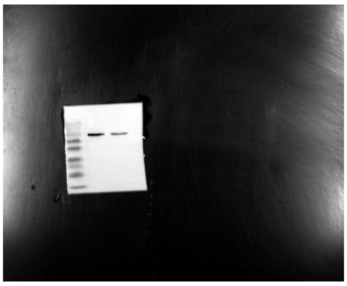
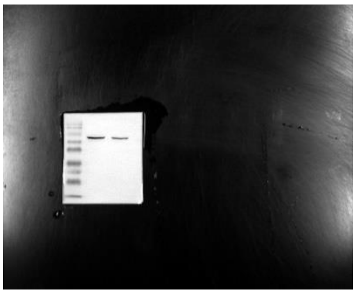

Nrf2 (110 kDa)

Repeat 1 Repeat 2 Repeat 3

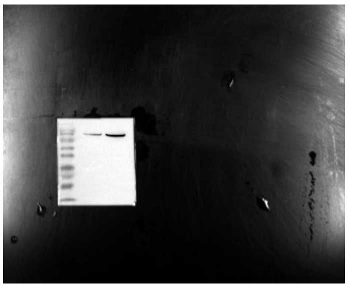
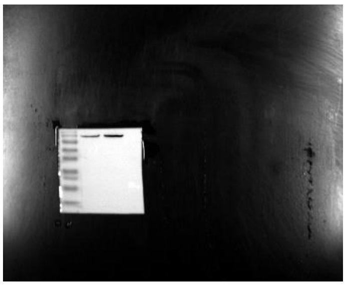
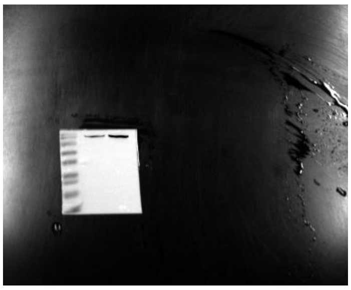

Repeat 4 Repeat 5

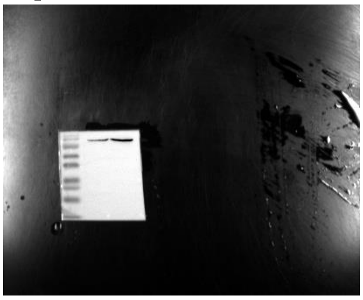
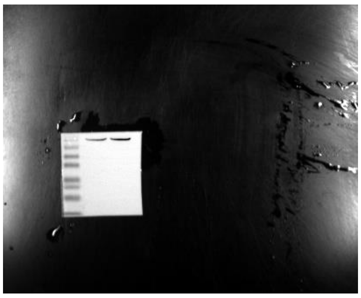

HIF-1α (92 kDa)

Repeat 1 Repeat 2 Repeat 3

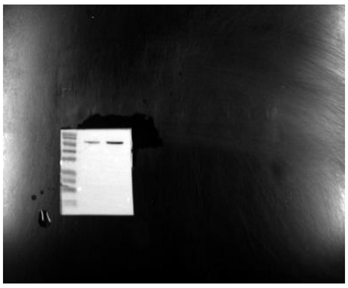
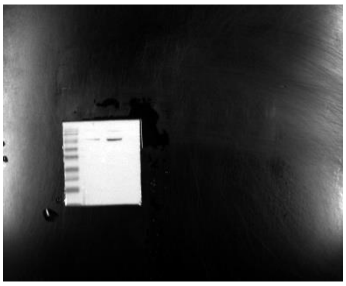
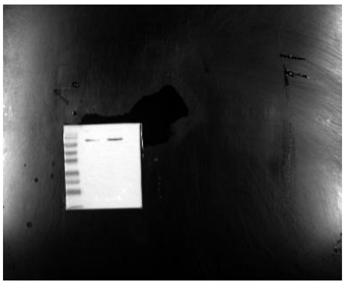

Repeat 4 Repeat 5

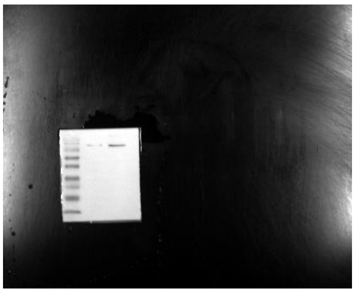
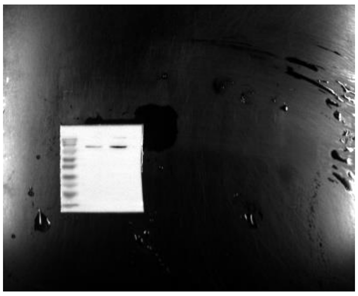

VEGF (180 kDa)

Repeat 1 Repeat 2 Repeat 3

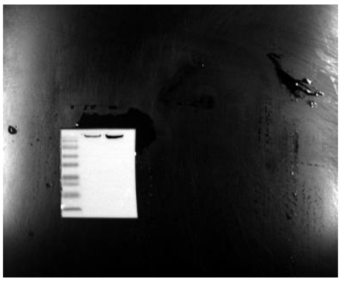
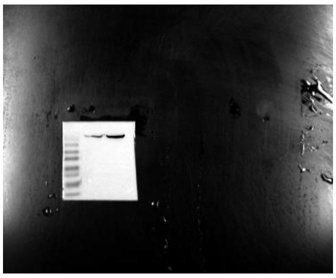
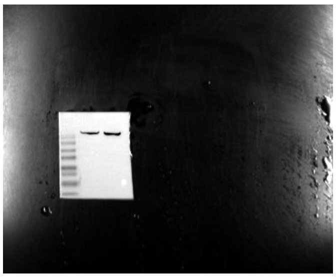

Repeat 4 Repeat 5

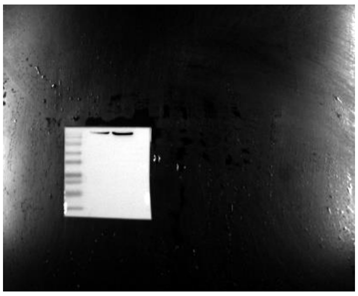 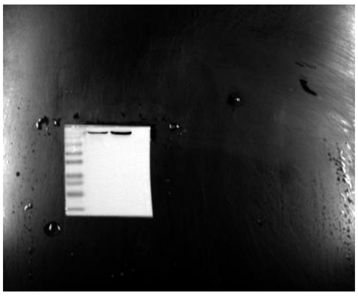

GAPDH (36KD)

Repeat 1 Repeat 2 Repeat 3

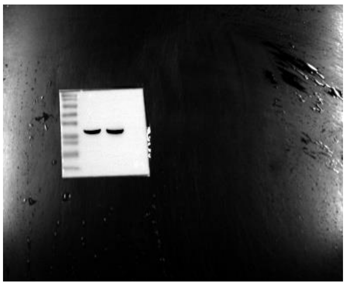 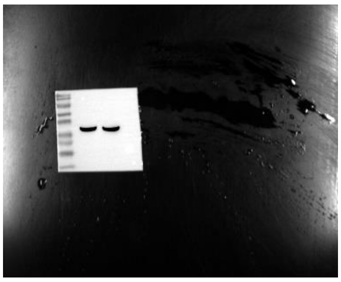 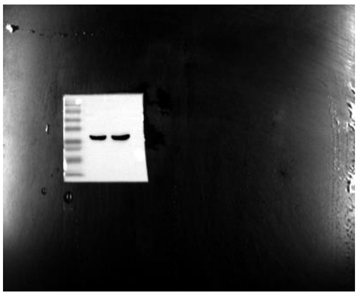

Repeat 4 Repeat 5

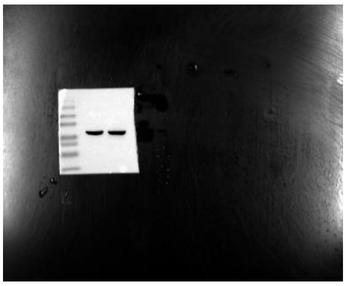 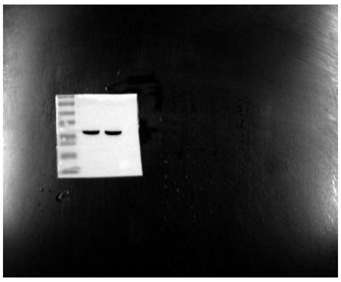

Supplement: S1 File — (PDF) [file pone.0325936.s001.pdf]
