# Supplementary figures and images for: PLZF promotes compensatory lung growth by increasing HPMEC proliferation and angiogenesis
Source: PLoS One. 2025 Jul 2;20(7):e0325936. doi: 10.1371/journal.pone.0325936 (PMC12221005; doi:10.1371/journal.pone.0325936)

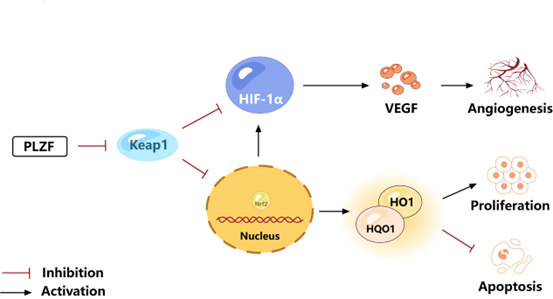

Supplement: S2 Fig — Potential mechanisms of PLZF regulation of HPMECs proliferation, apoptosis, and angiogenesis. (JPG) [file pone.0325936.s002.jpg]

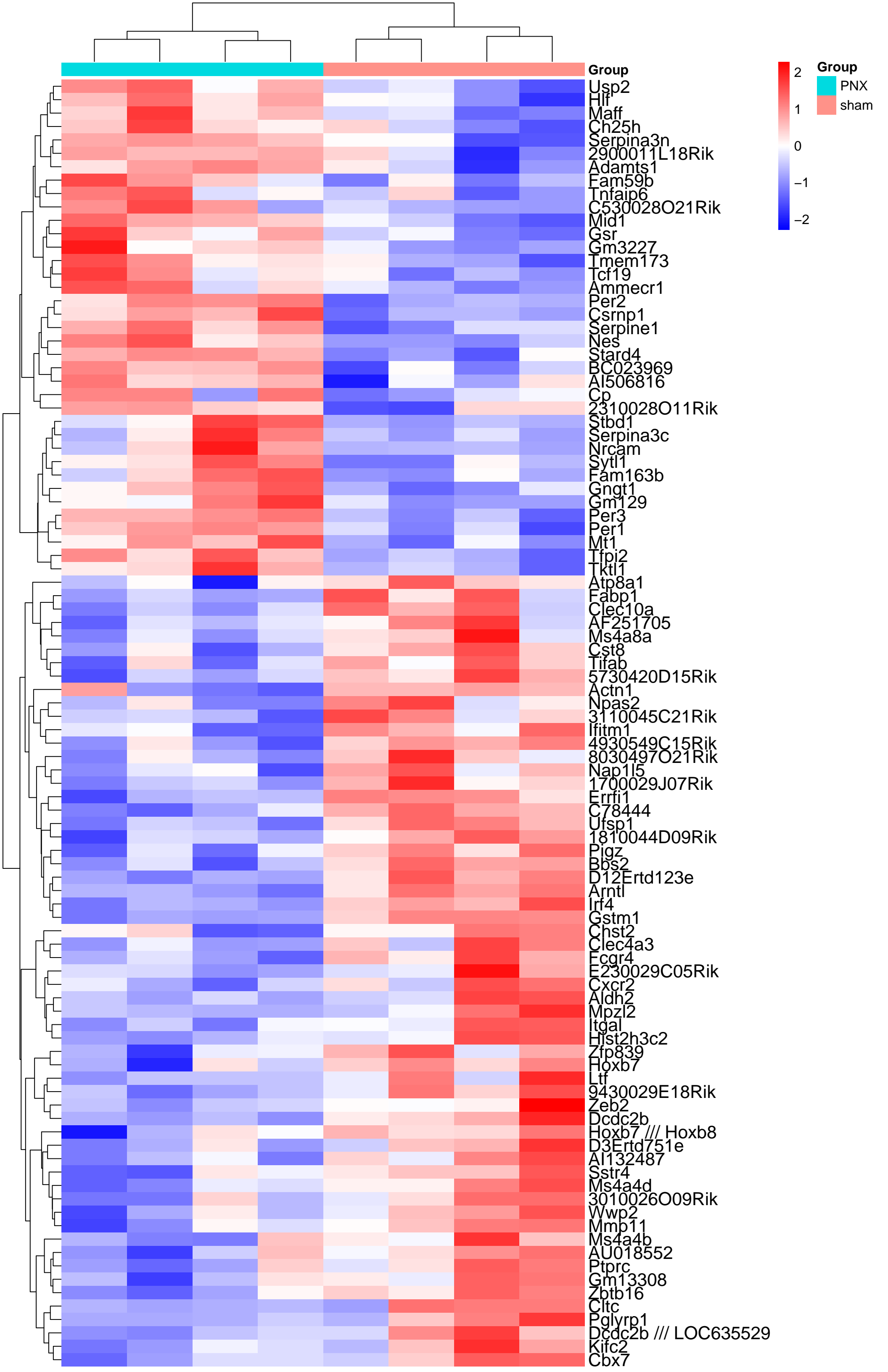

Supplement: S4 File — (ZIP) [file pone.0325936.s004.zip › Raw data 1/Figure 1/images/1A.pdf]

EnhancedVolcano

Not Sig   Log2FC   Pvalue   Pvalue and Log2FC

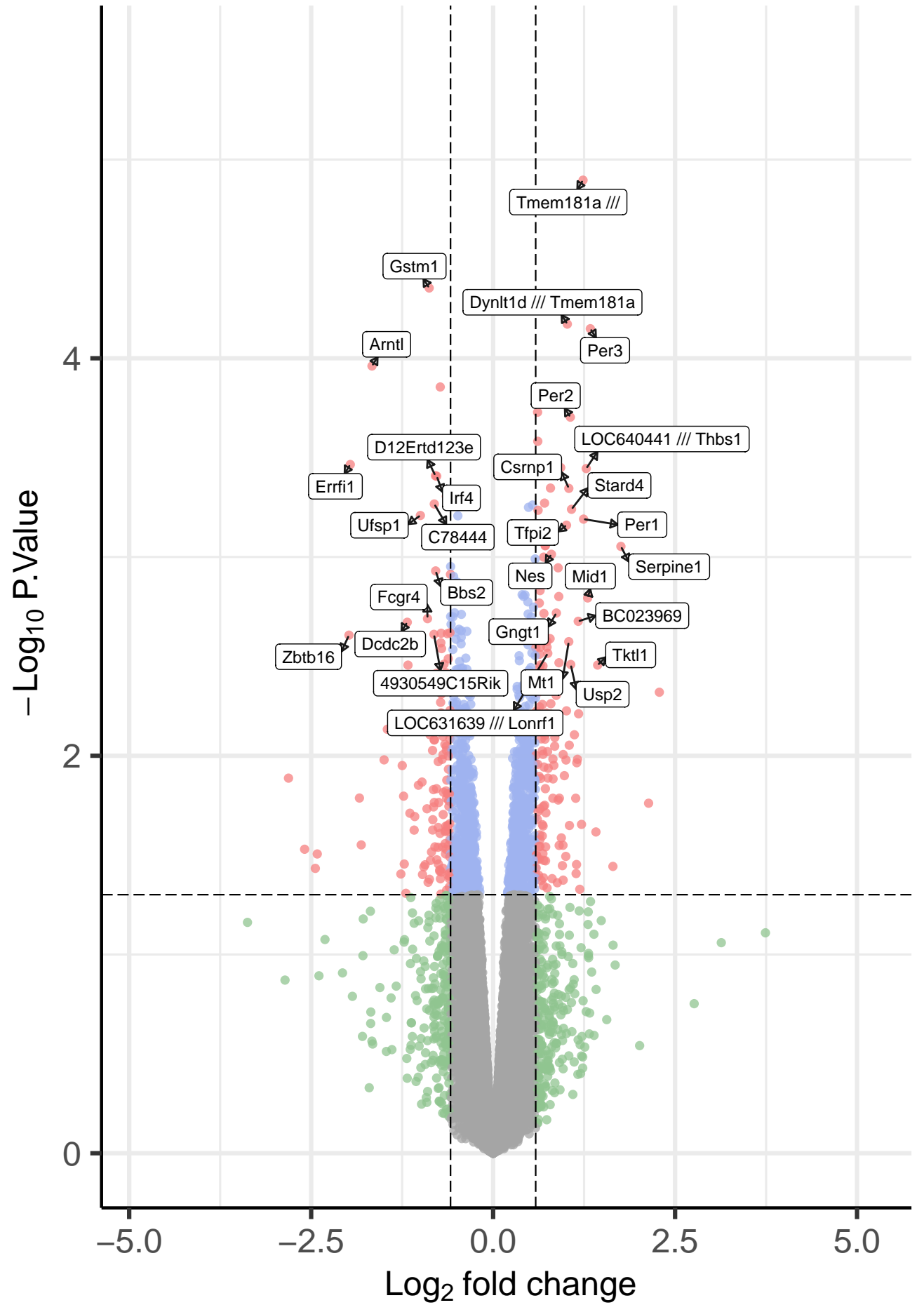

total = 21761 variables

Supplement: S4 File — (ZIP) [file pone.0325936.s004.zip › Raw data 1/Figure 1/images/1B.pdf]

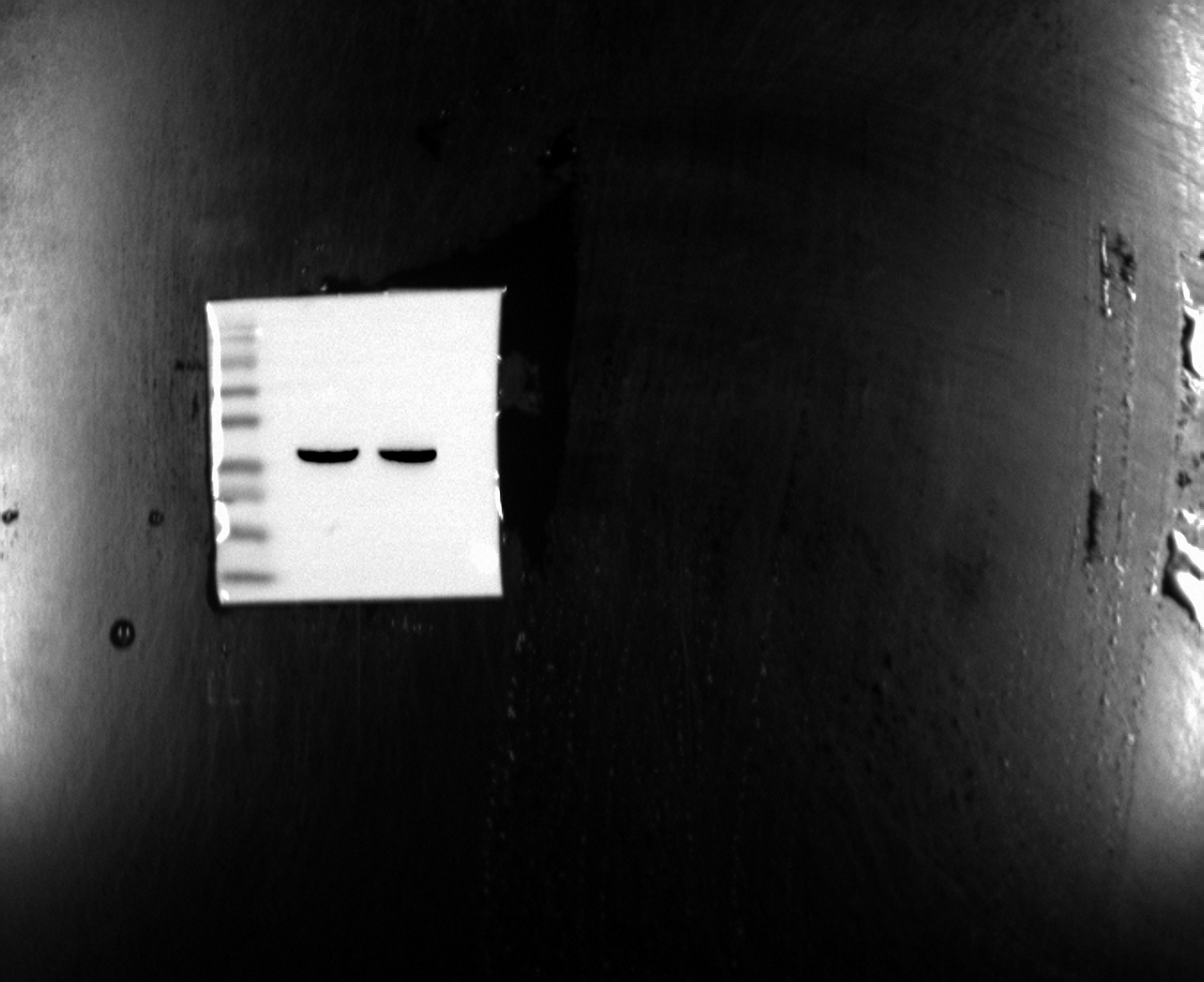

Supplement: S4 File — (ZIP) [file pone.0325936.s004.zip › Raw data 1/Figure 1/images/1C/DAPDH.tif]

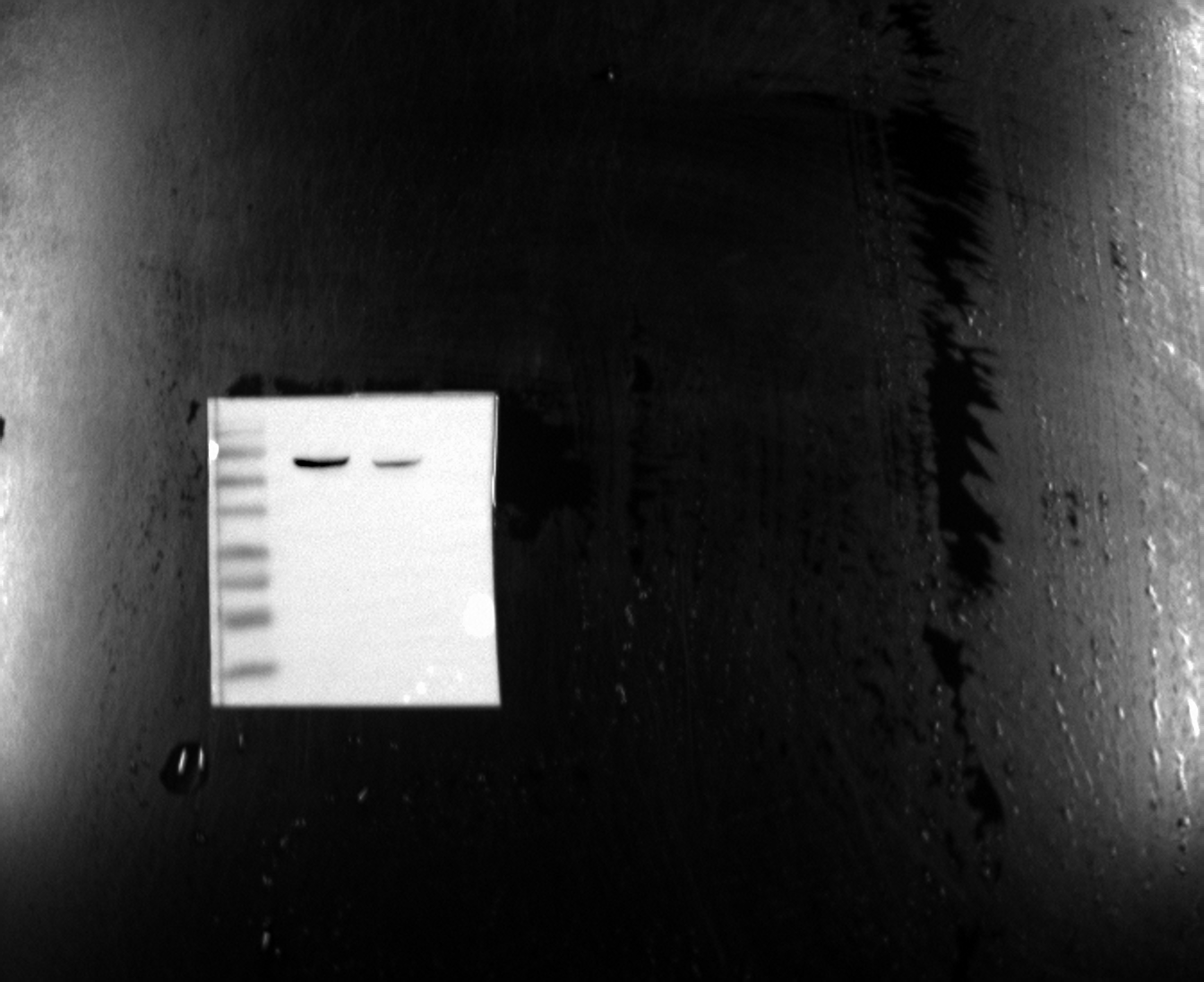

Supplement: S4 File — (ZIP) [file pone.0325936.s004.zip › Raw data 1/Figure 1/images/1C/PLZF.tif]

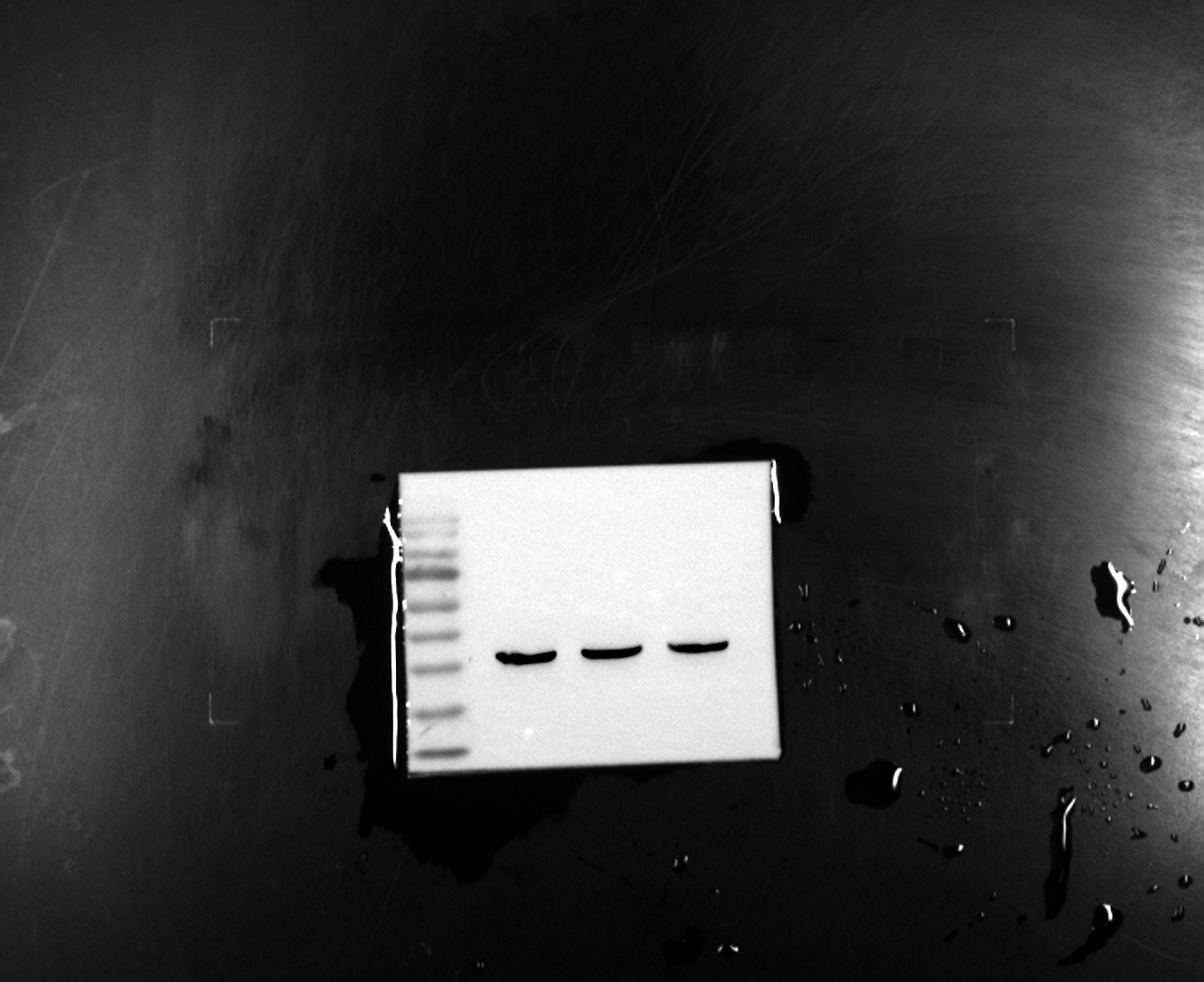

Supplement: S4 File — (ZIP) [file pone.0325936.s004.zip › Raw data 1/Figure 2/images/2A/GAPDH.tif]

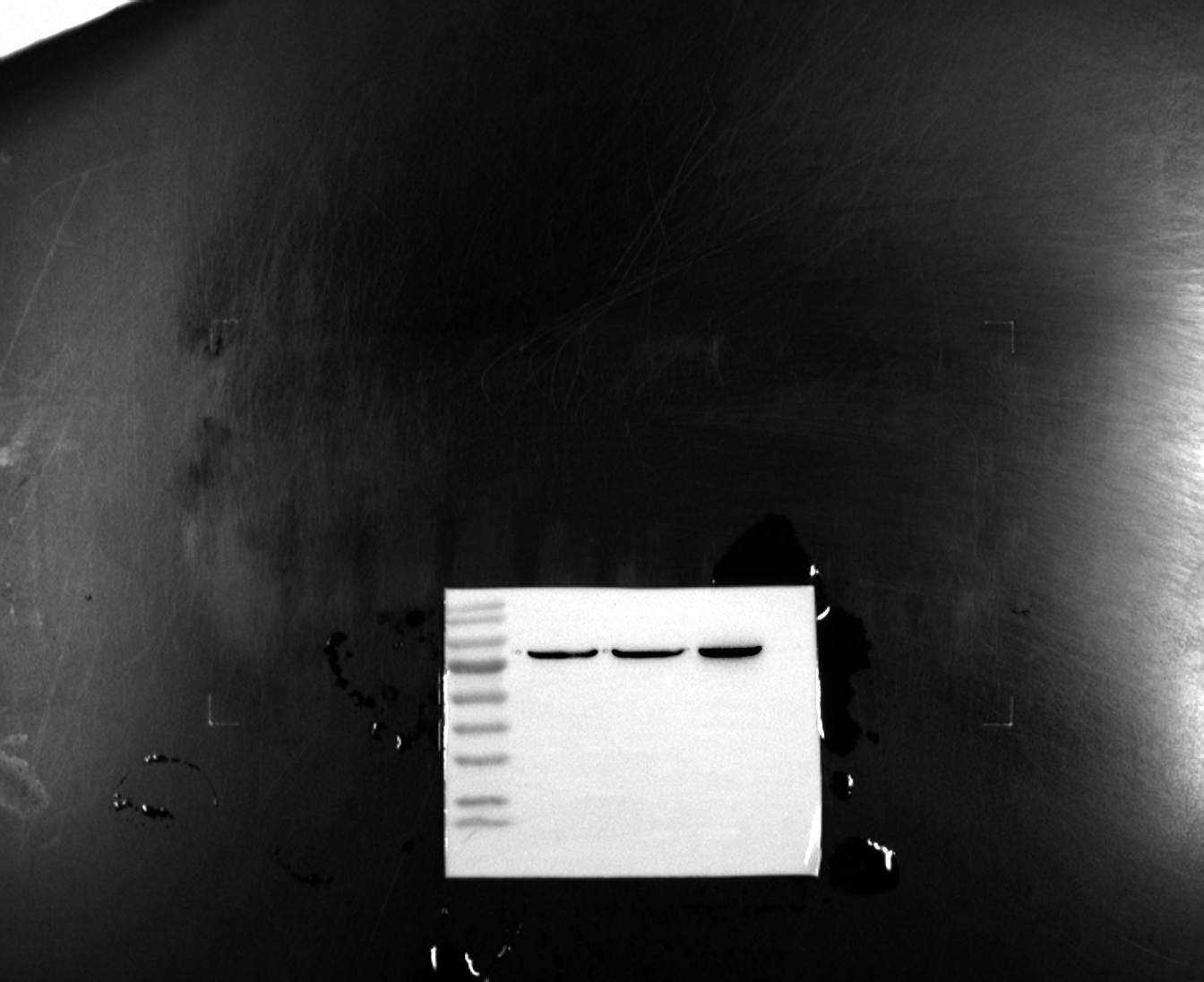

Supplement: S4 File — (ZIP) [file pone.0325936.s004.zip › Raw data 1/Figure 2/images/2A/PLZF.tif]

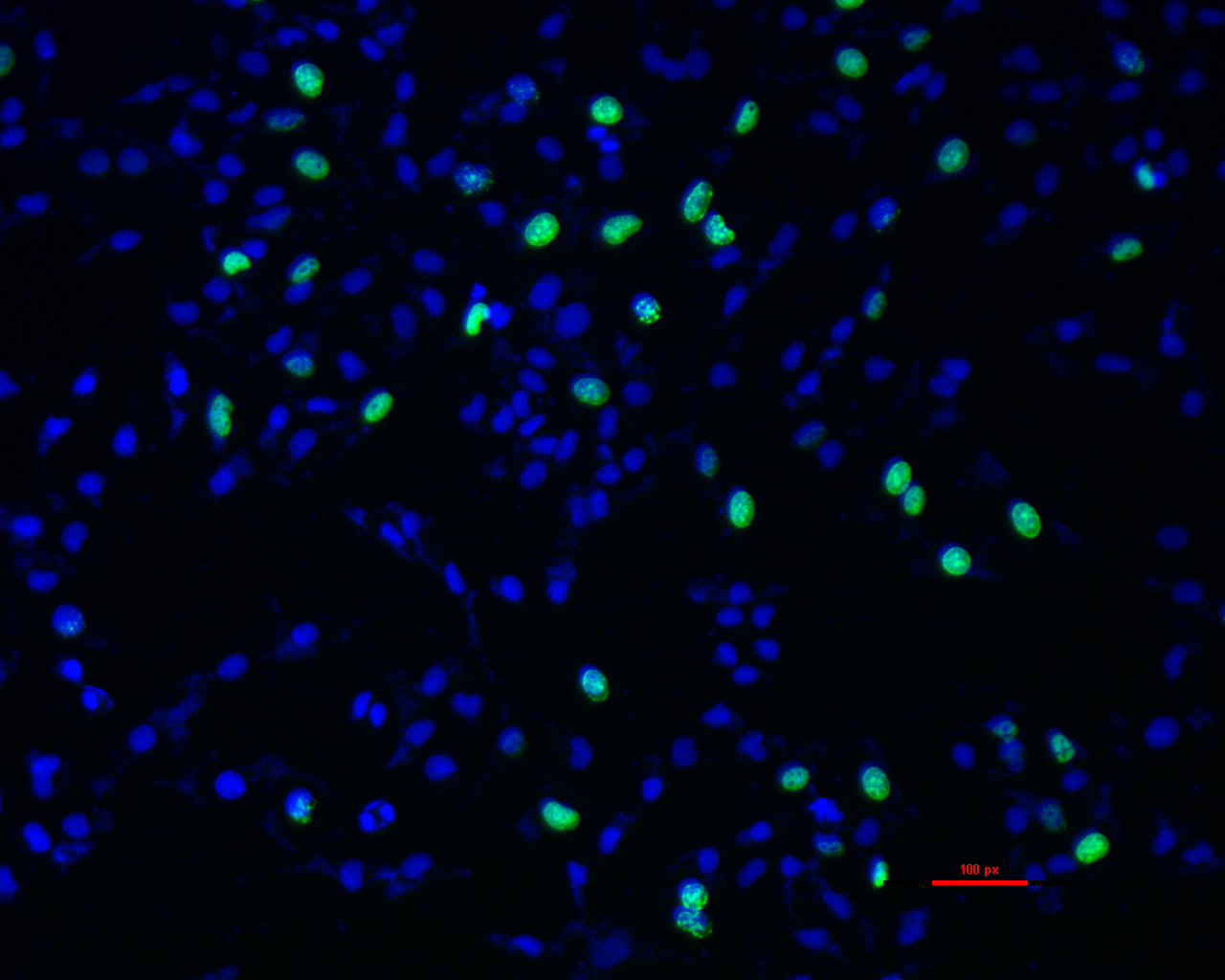

Supplement: S4 File — (ZIP) [file pone.0325936.s004.zip › Raw data 1/Figure 2/images/2C/OE-NC.tif]

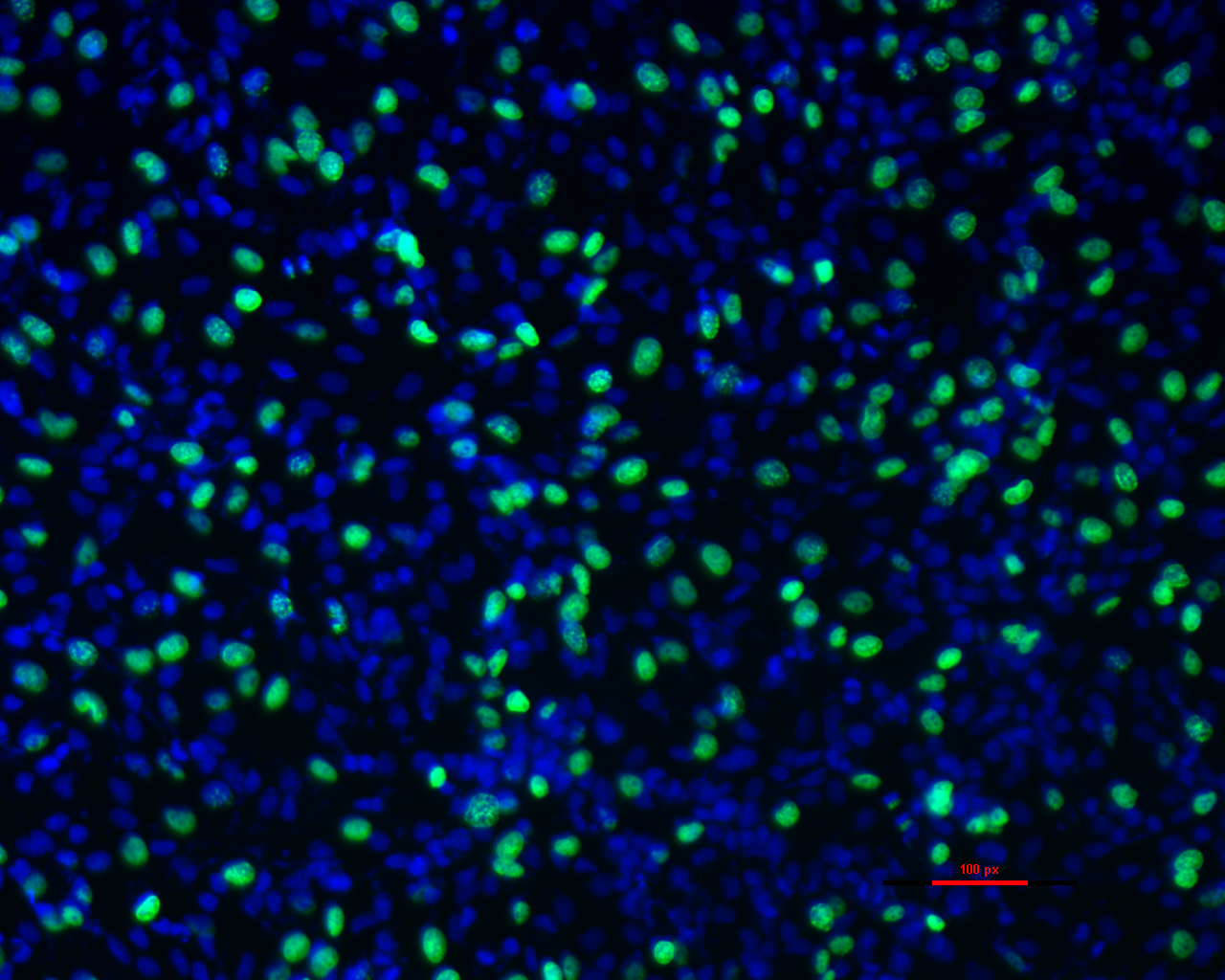

Supplement: S4 File — (ZIP) [file pone.0325936.s004.zip › Raw data 1/Figure 2/images/2C/OE-PLZF.tif]

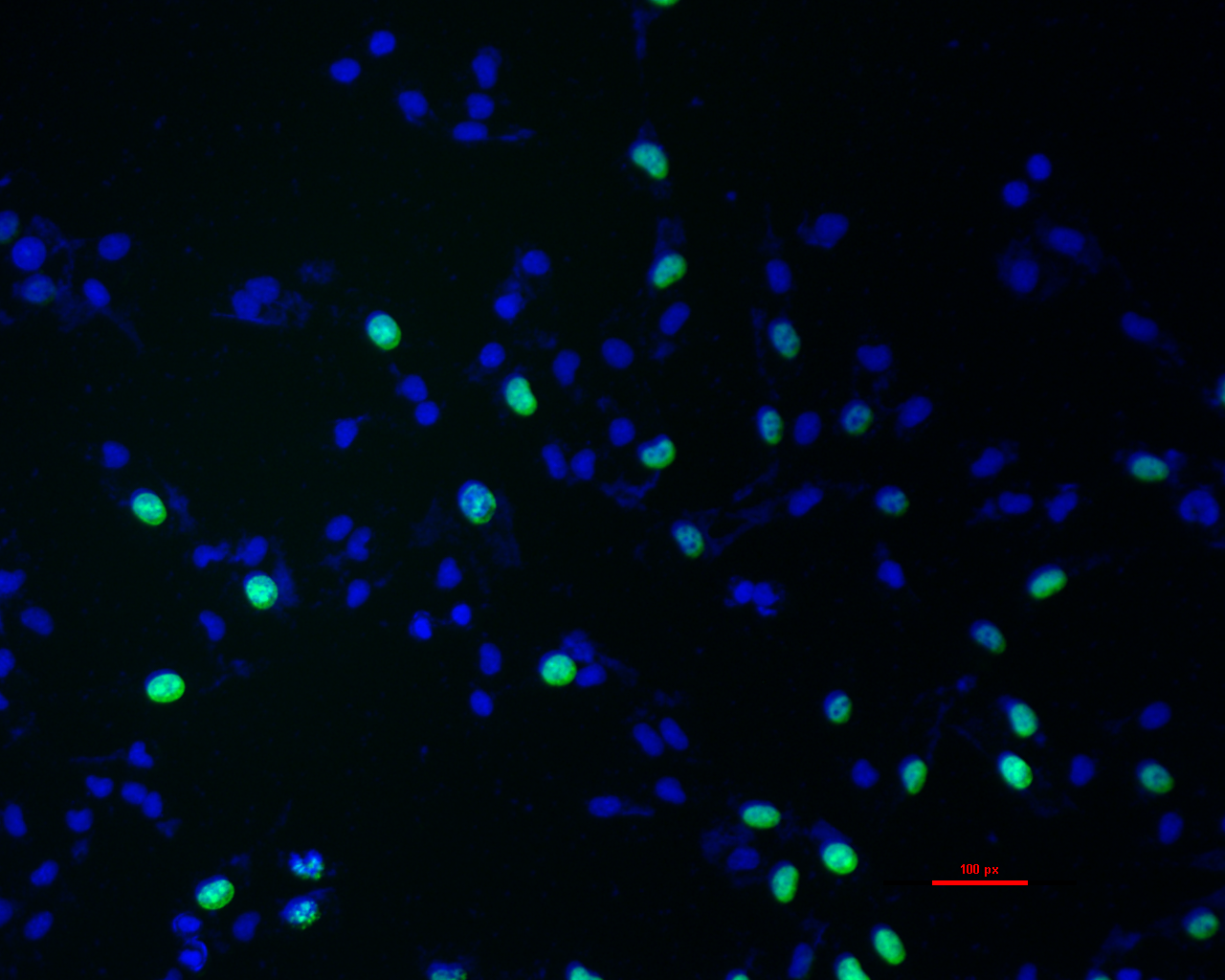

Supplement: S4 File — (ZIP) [file pone.0325936.s004.zip › Raw data 1/Figure 2/images/2C/control.tif]

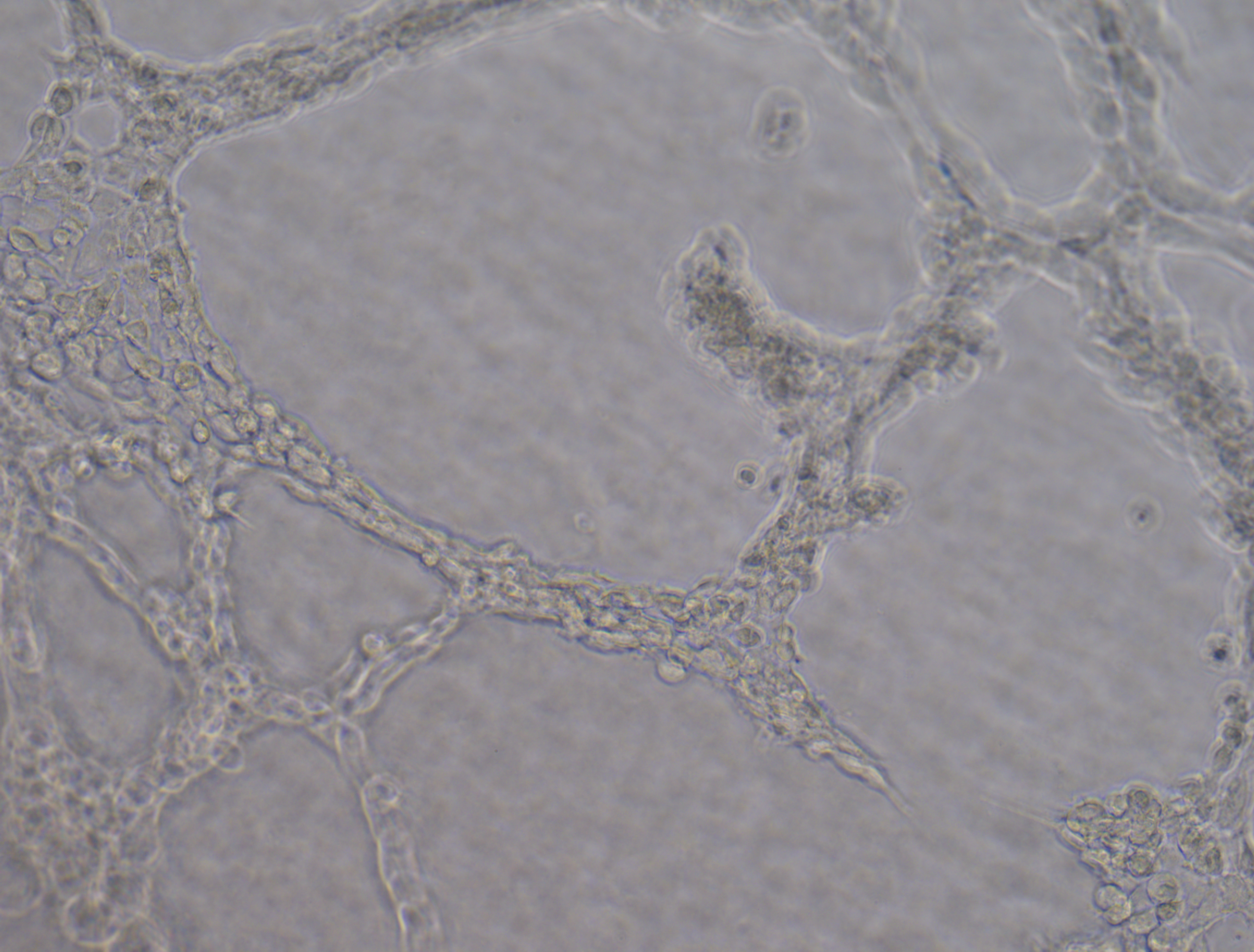

Supplement: S4 File — (ZIP) [file pone.0325936.s004.zip › Raw data 1/Figure 2/images/2D/Control.tif]

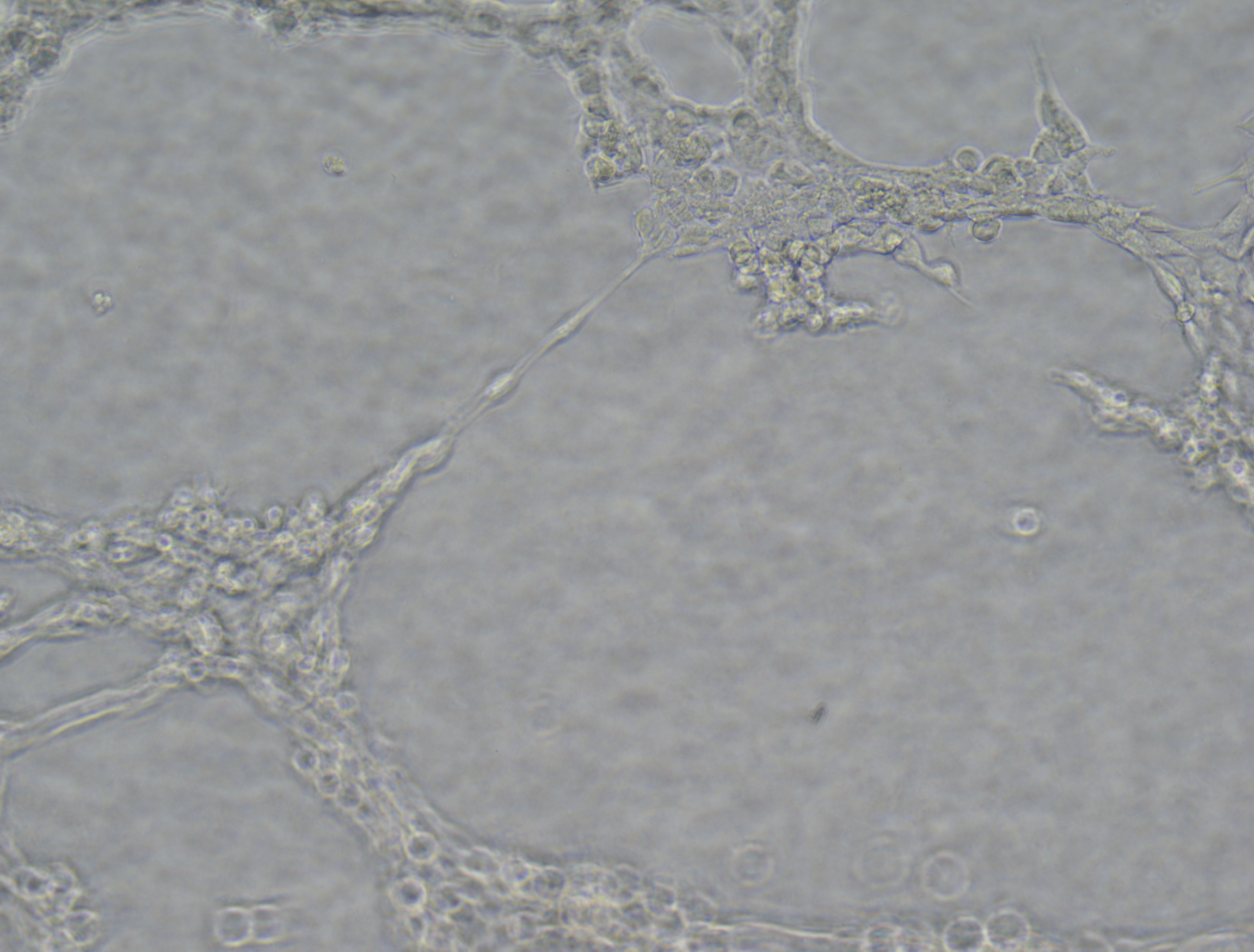

Supplement: S4 File — (ZIP) [file pone.0325936.s004.zip › Raw data 1/Figure 2/images/2D/OE-NC.tif]

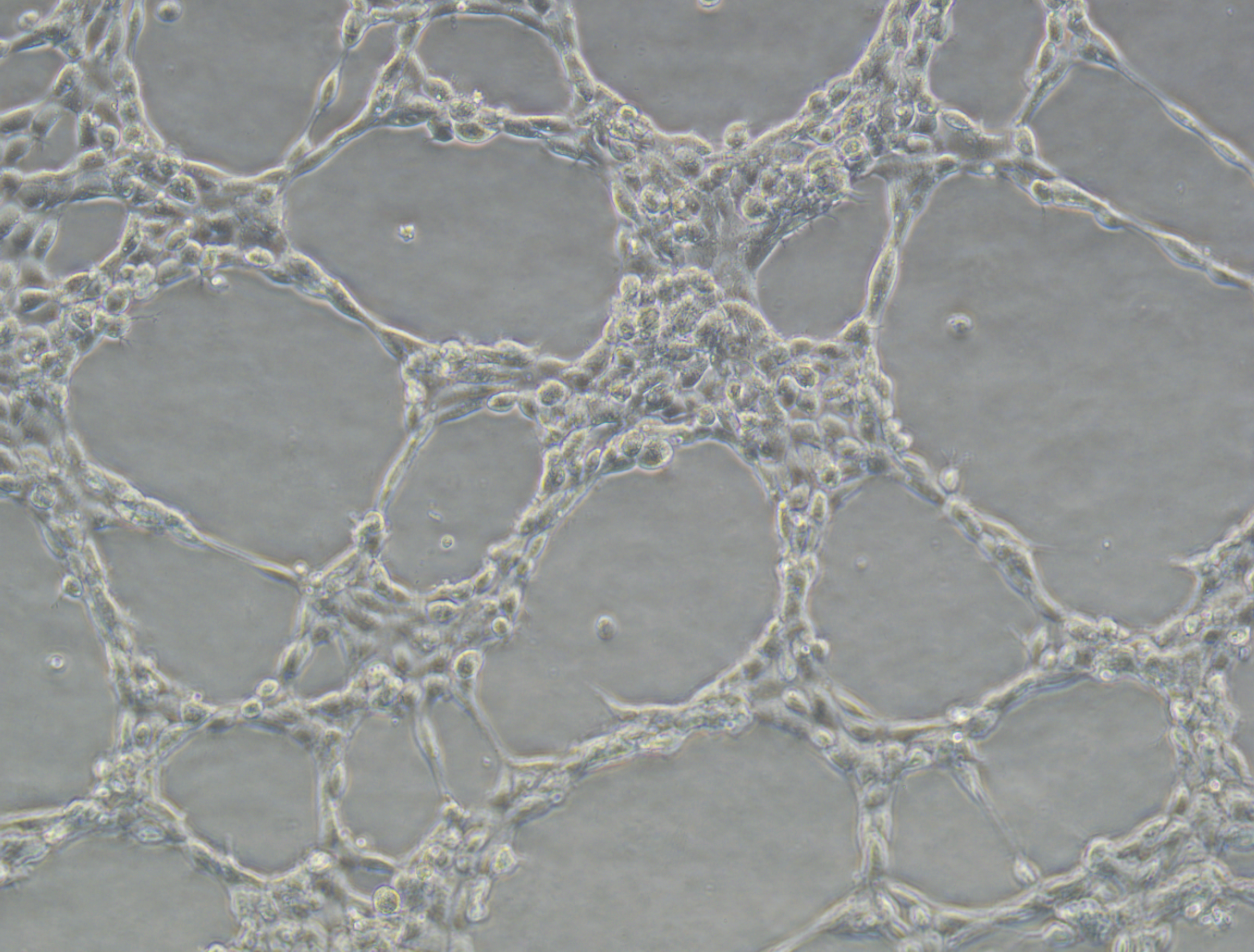

Supplement: S4 File — (ZIP) [file pone.0325936.s004.zip › Raw data 1/Figure 2/images/2D/OE-PLZF.tif]

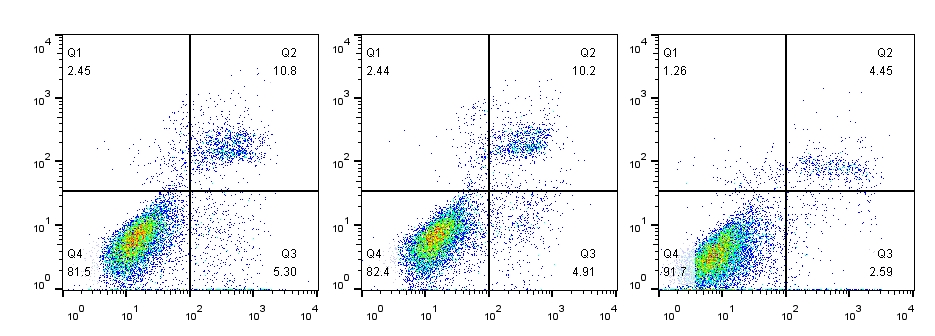

Supplement: S4 File — (ZIP) [file pone.0325936.s004.zip › Raw data 1/Figure 2/images/2E/2E.jpg]

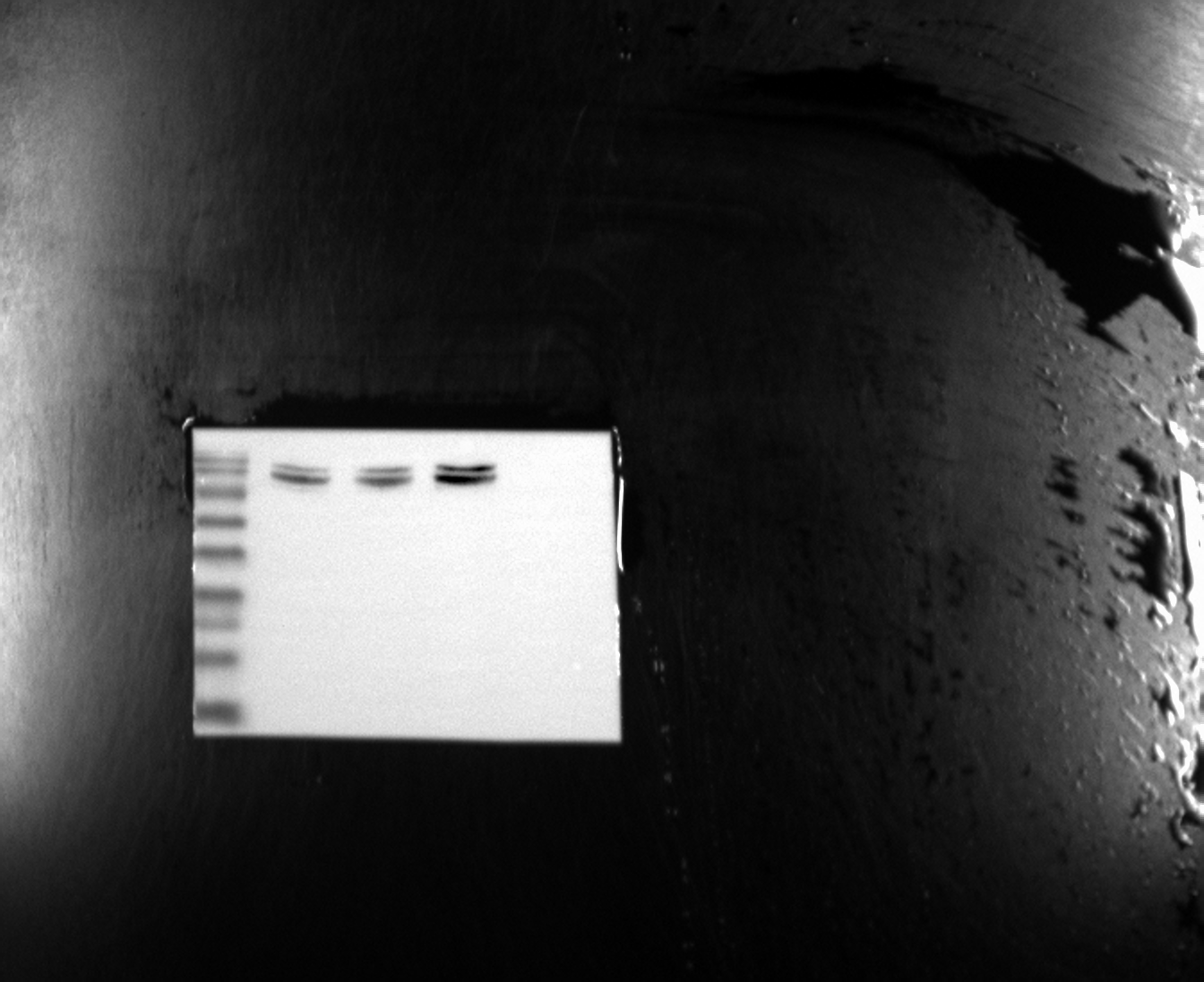

Supplement: S4 File — (ZIP) [file pone.0325936.s004.zip › Raw data 1/Figure 2/images/2F/CD31.tif]

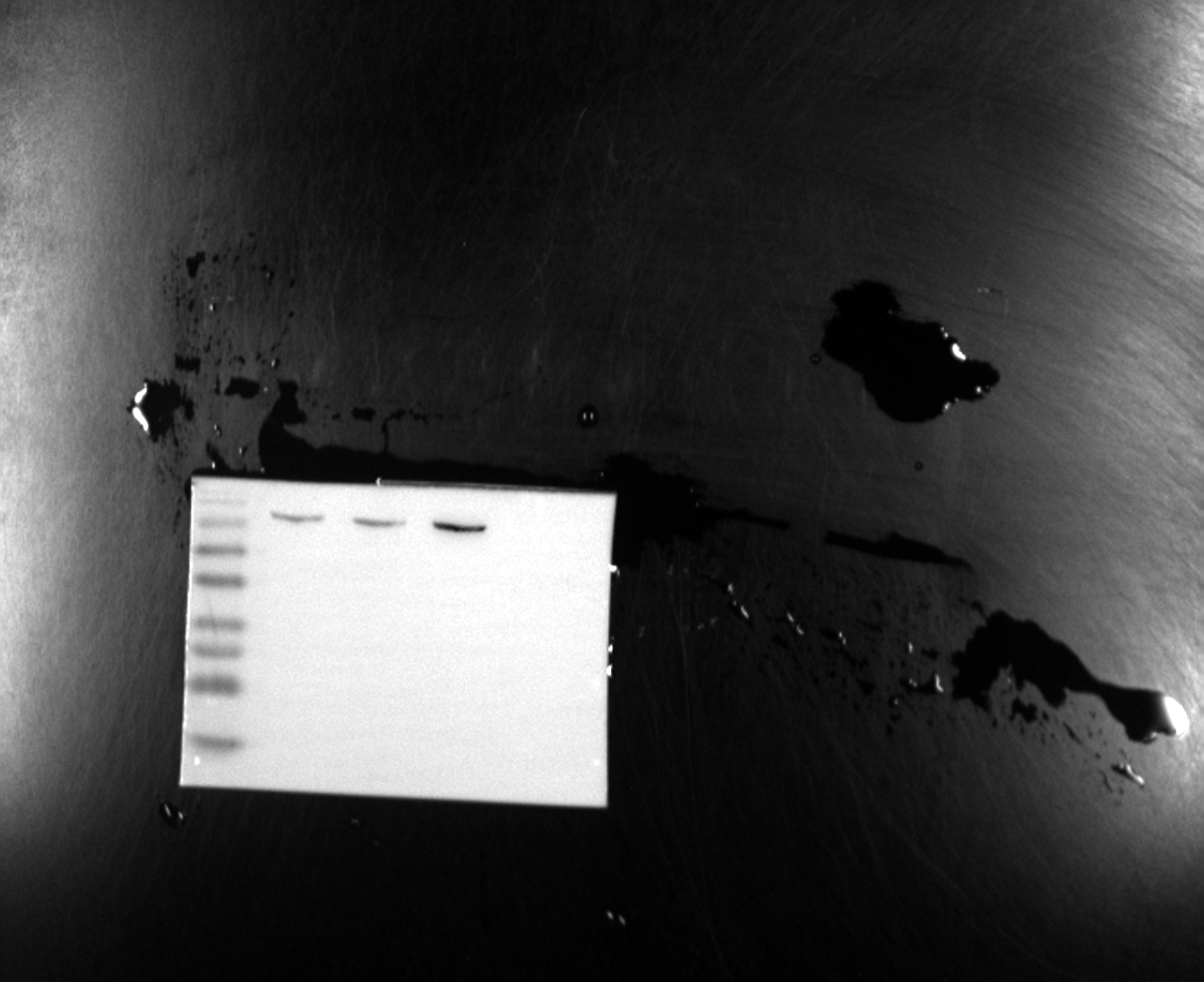

Supplement: S4 File — (ZIP) [file pone.0325936.s004.zip › Raw data 1/Figure 2/images/2F/CD34.tif]

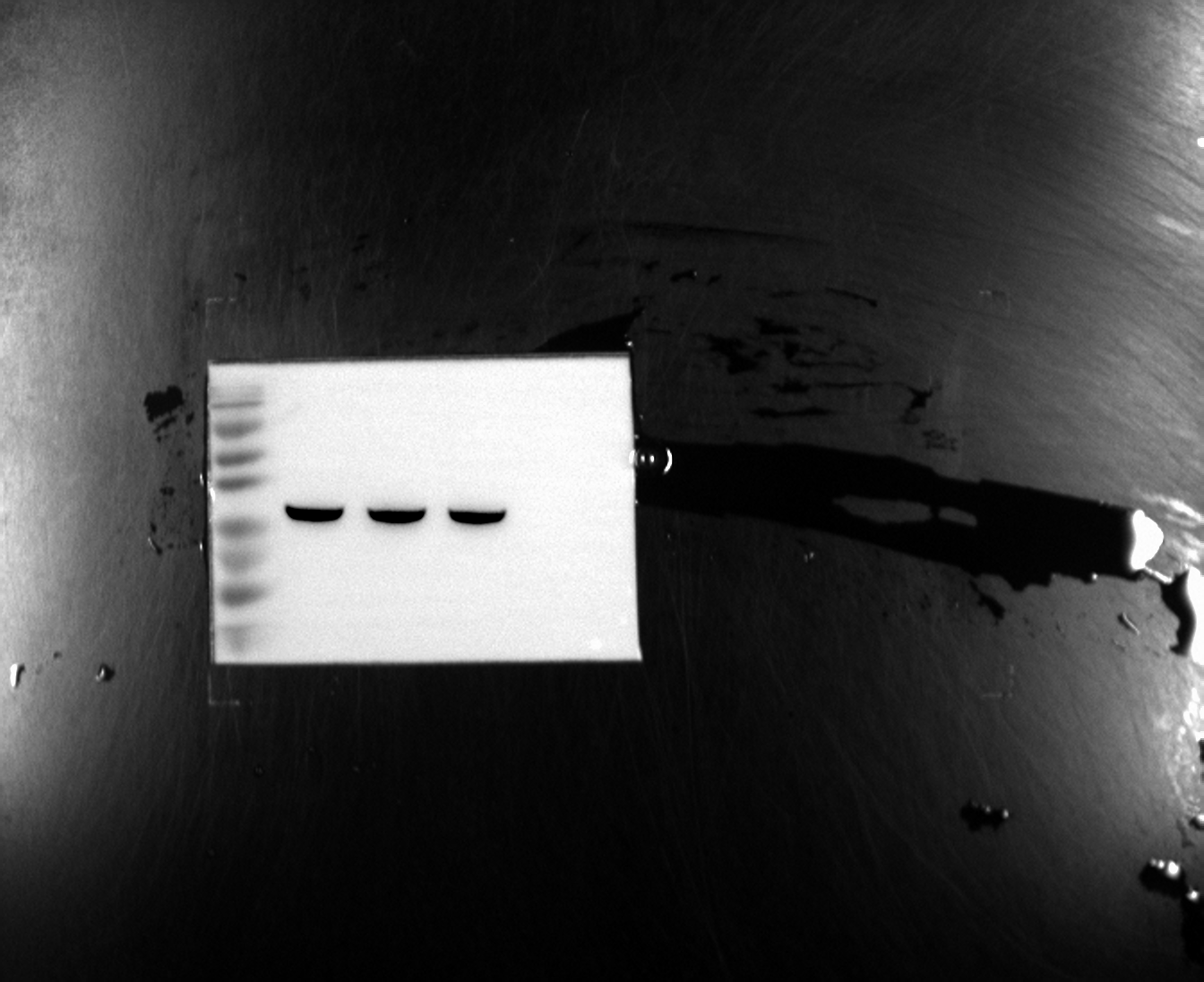

Supplement: S4 File — (ZIP) [file pone.0325936.s004.zip › Raw data 1/Figure 2/images/2F/GAPDH.tif]

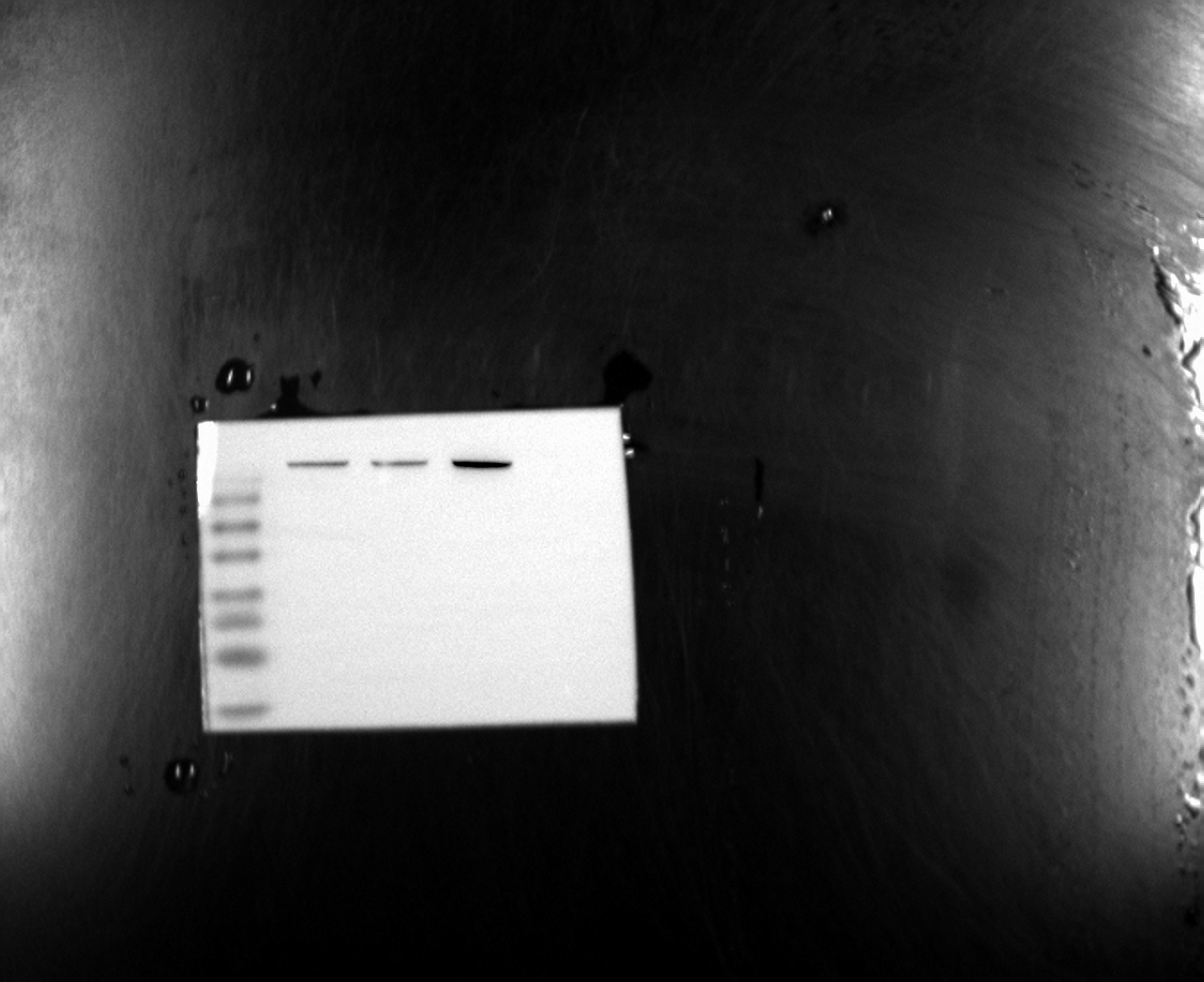

Supplement: S4 File — (ZIP) [file pone.0325936.s004.zip › Raw data 1/Figure 2/images/2F/VEGF.tif]

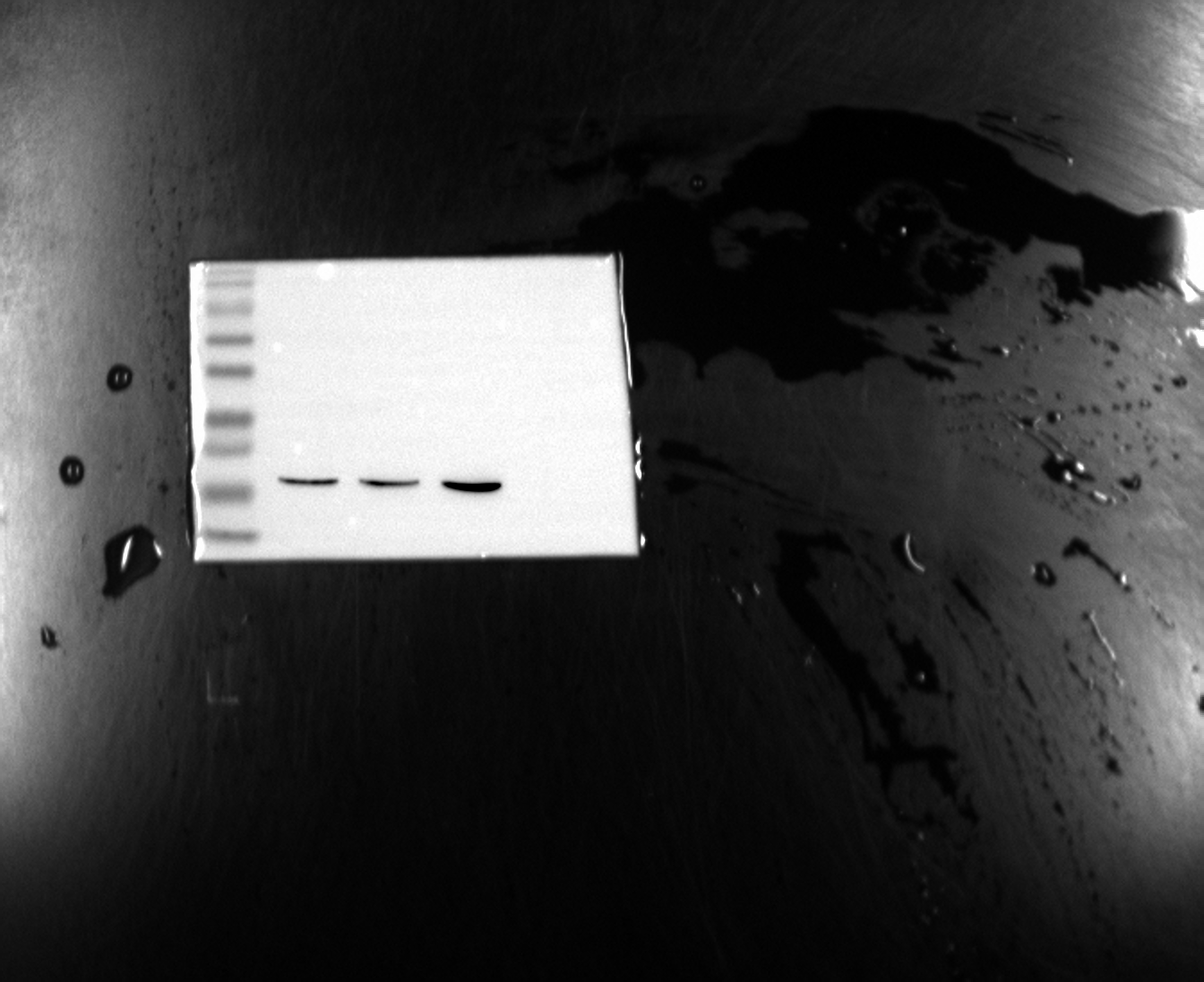

Supplement: S4 File — (ZIP) [file pone.0325936.s004.zip › Raw data 1/Figure 2/images/2F/bFGF.tif]

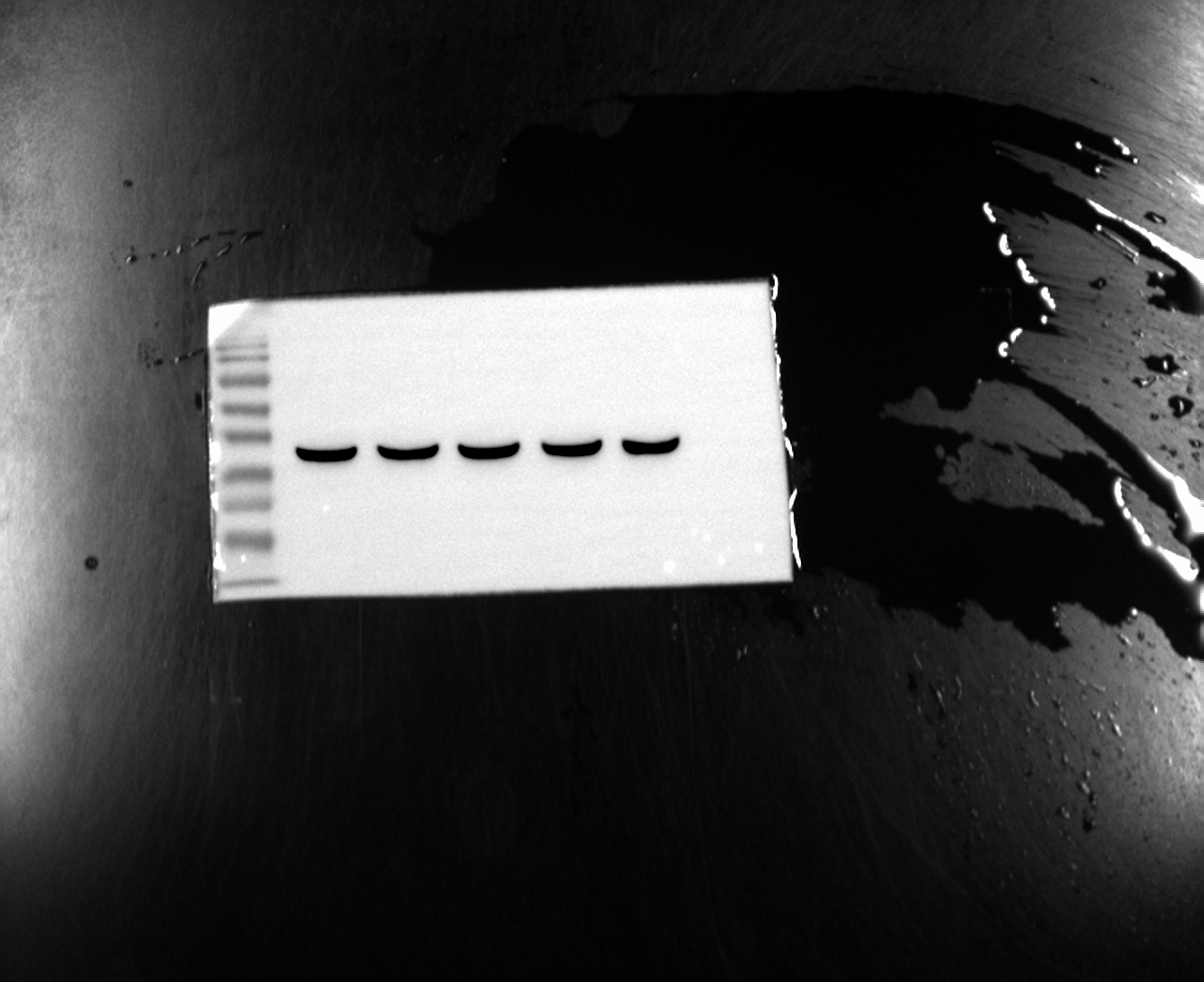

Supplement: S7 File — (ZIP) [file pone.0325936.s007.zip › Raw data 4/Figure 5/images/5A/GAPDH.tif]

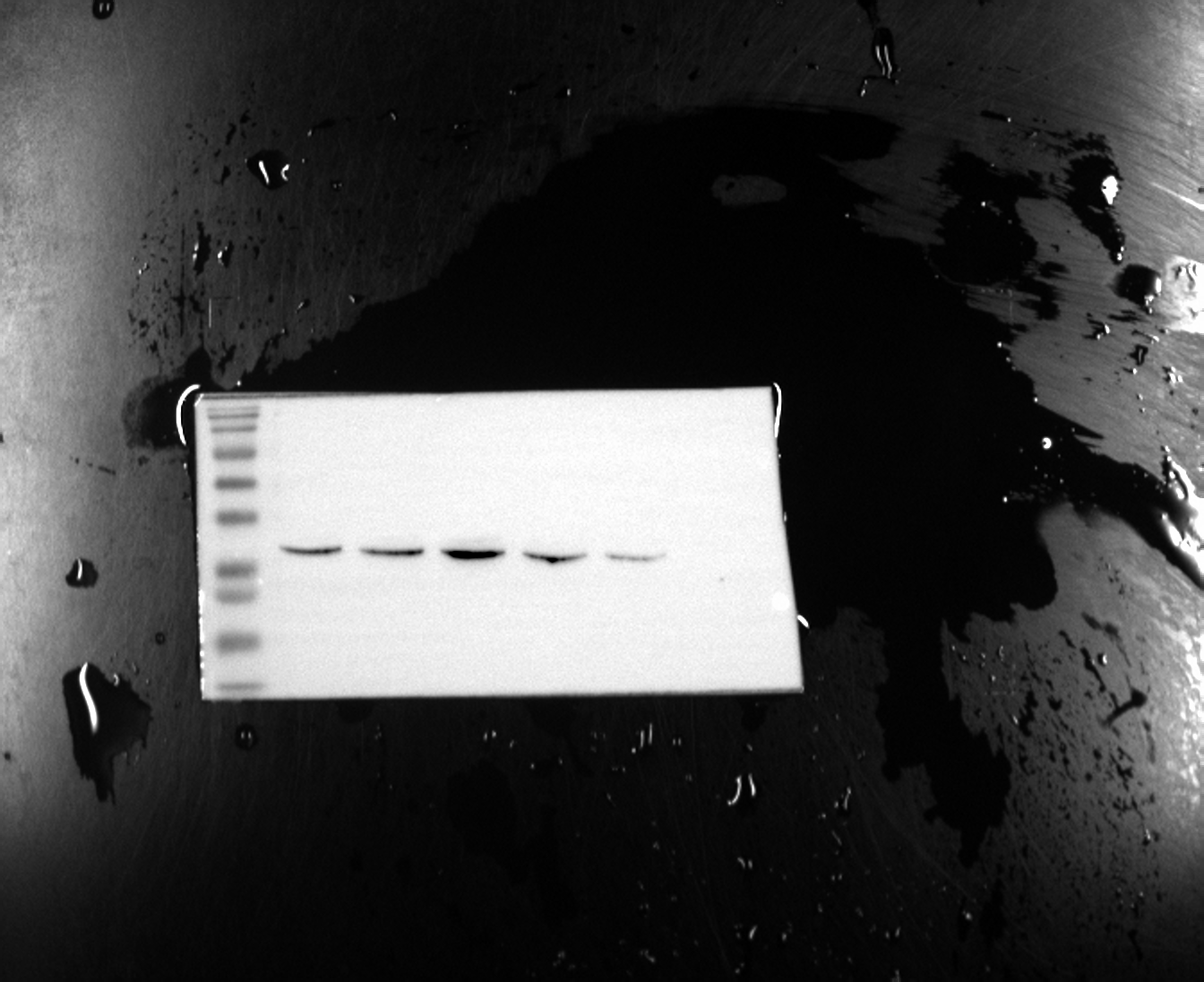

Supplement: S7 File — (ZIP) [file pone.0325936.s007.zip › Raw data 4/Figure 5/images/5A/HO-1.tif]

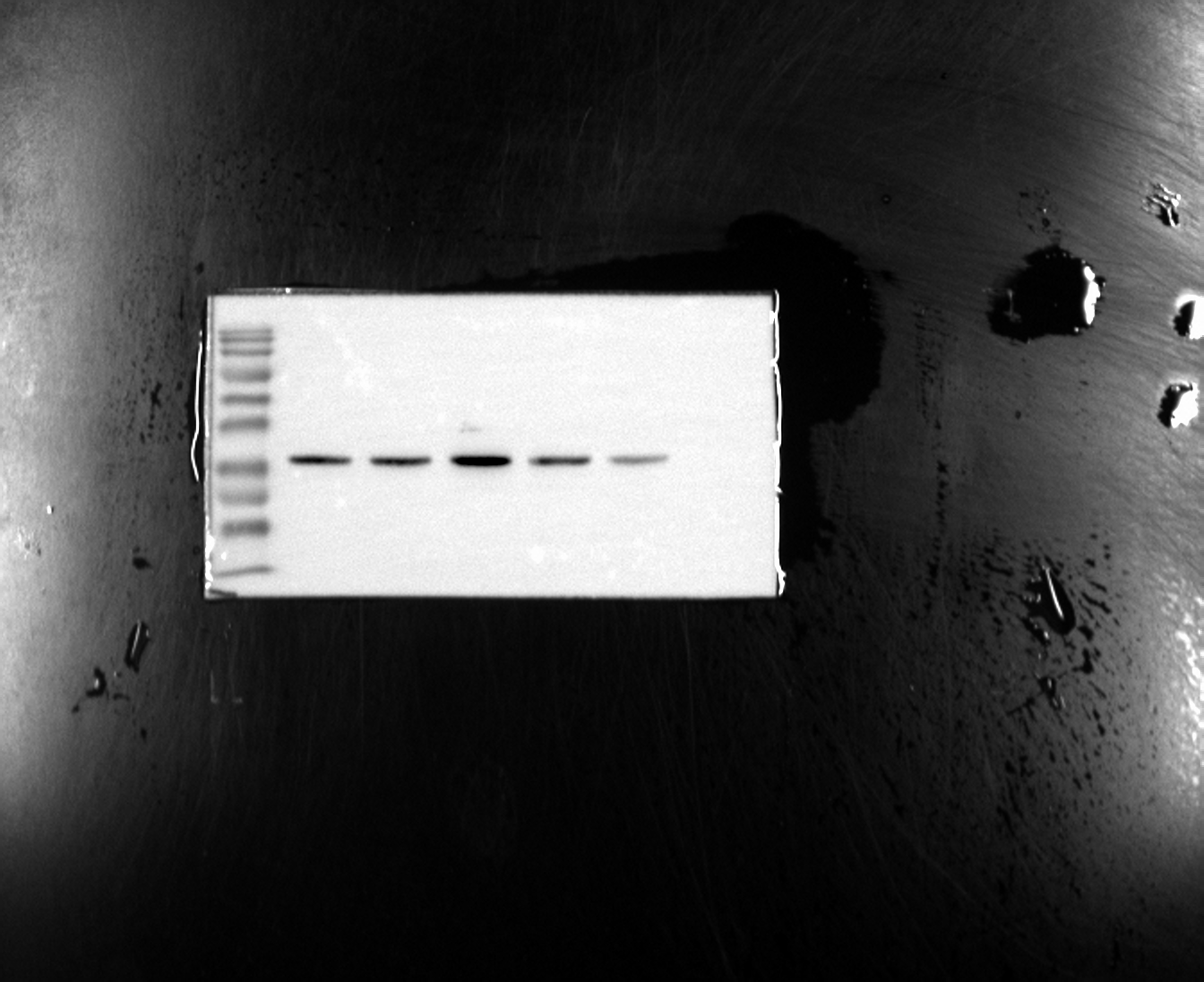

Supplement: S7 File — (ZIP) [file pone.0325936.s007.zip › Raw data 4/Figure 5/images/5A/NQO1.tif]

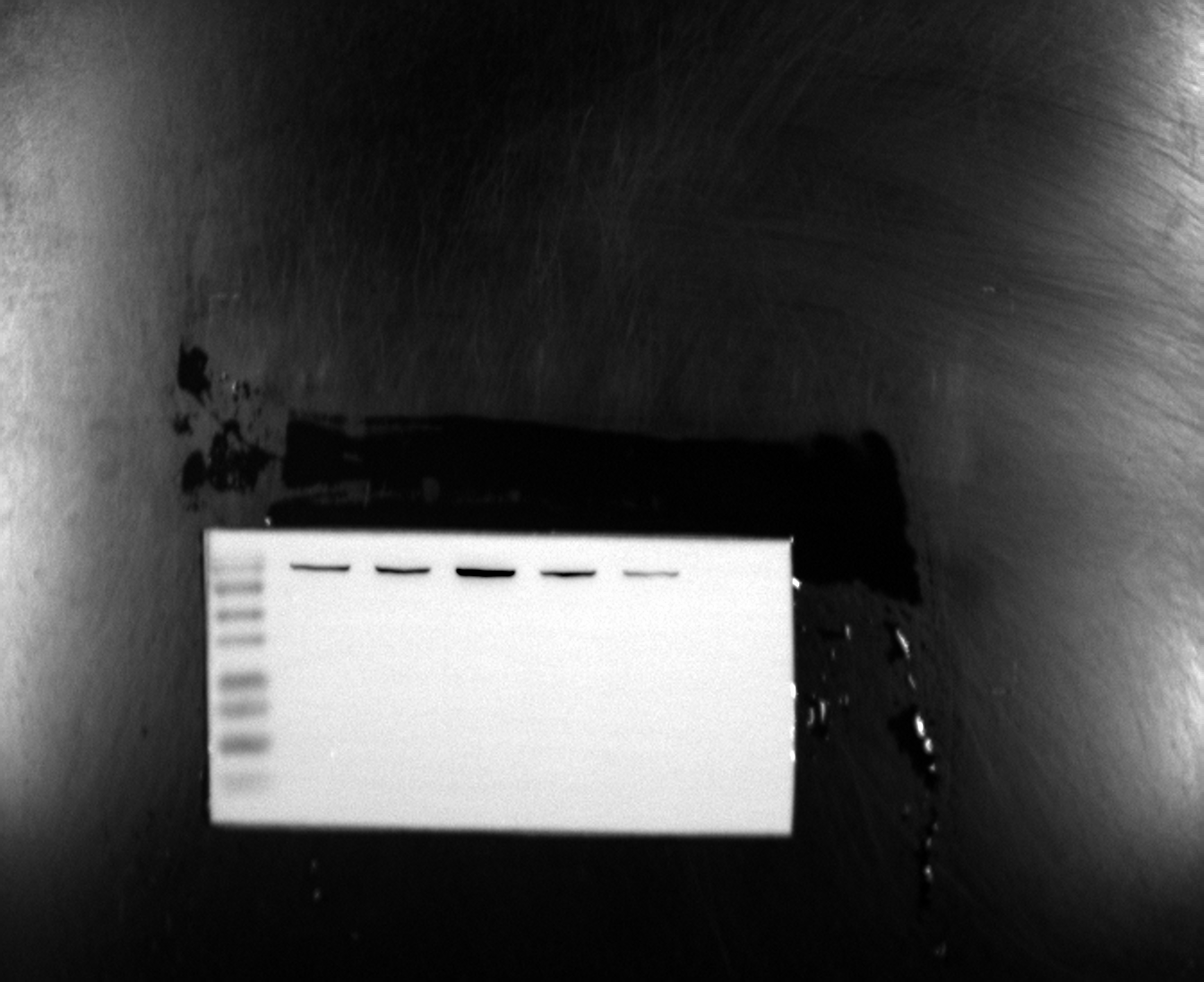

Supplement: S7 File — (ZIP) [file pone.0325936.s007.zip › Raw data 4/Figure 5/images/5A/Nrf2.tif]

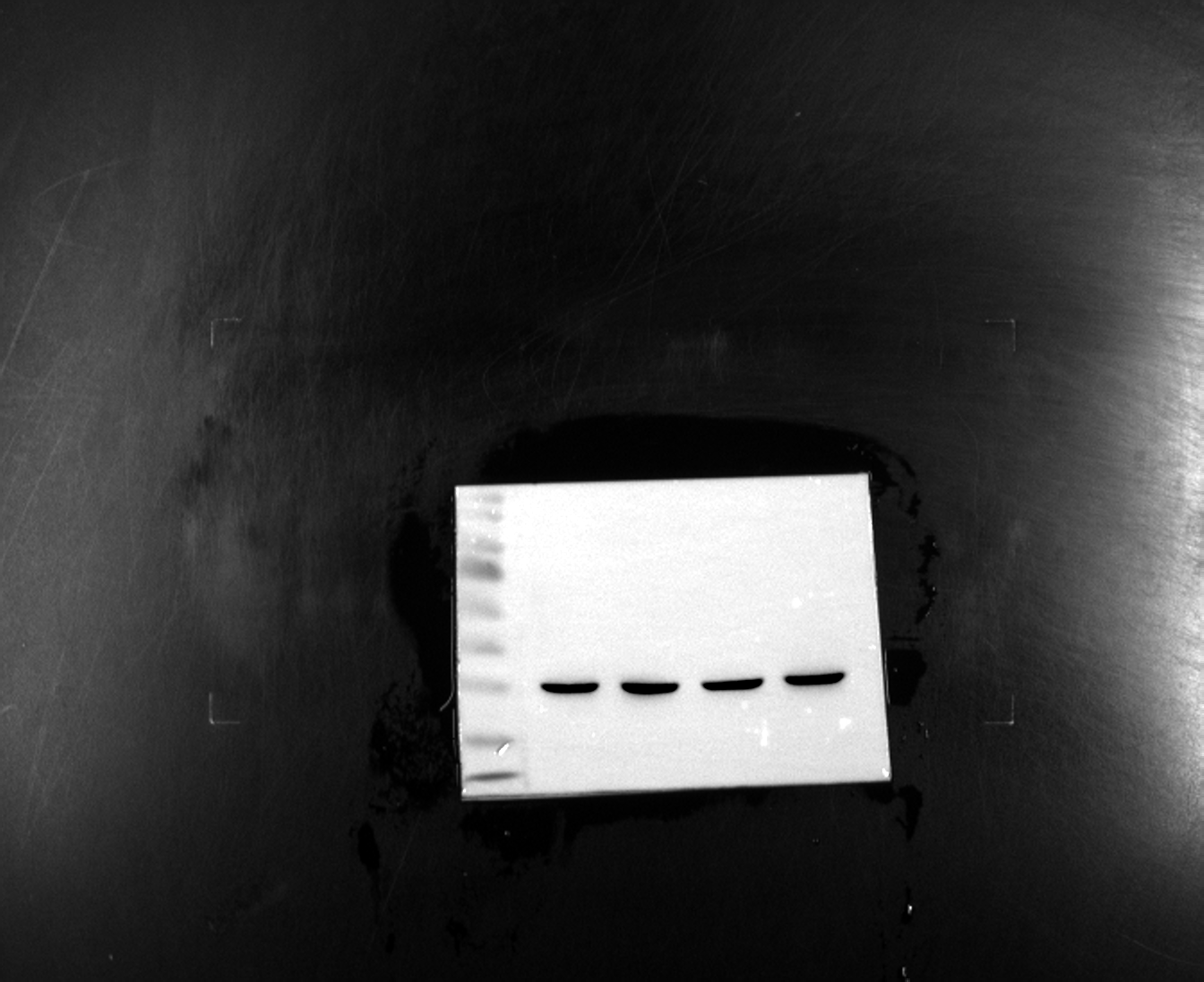

Supplement: S7 File — (ZIP) [file pone.0325936.s007.zip › Raw data 4/Figure 5/images/5C/GAPDH.tif]

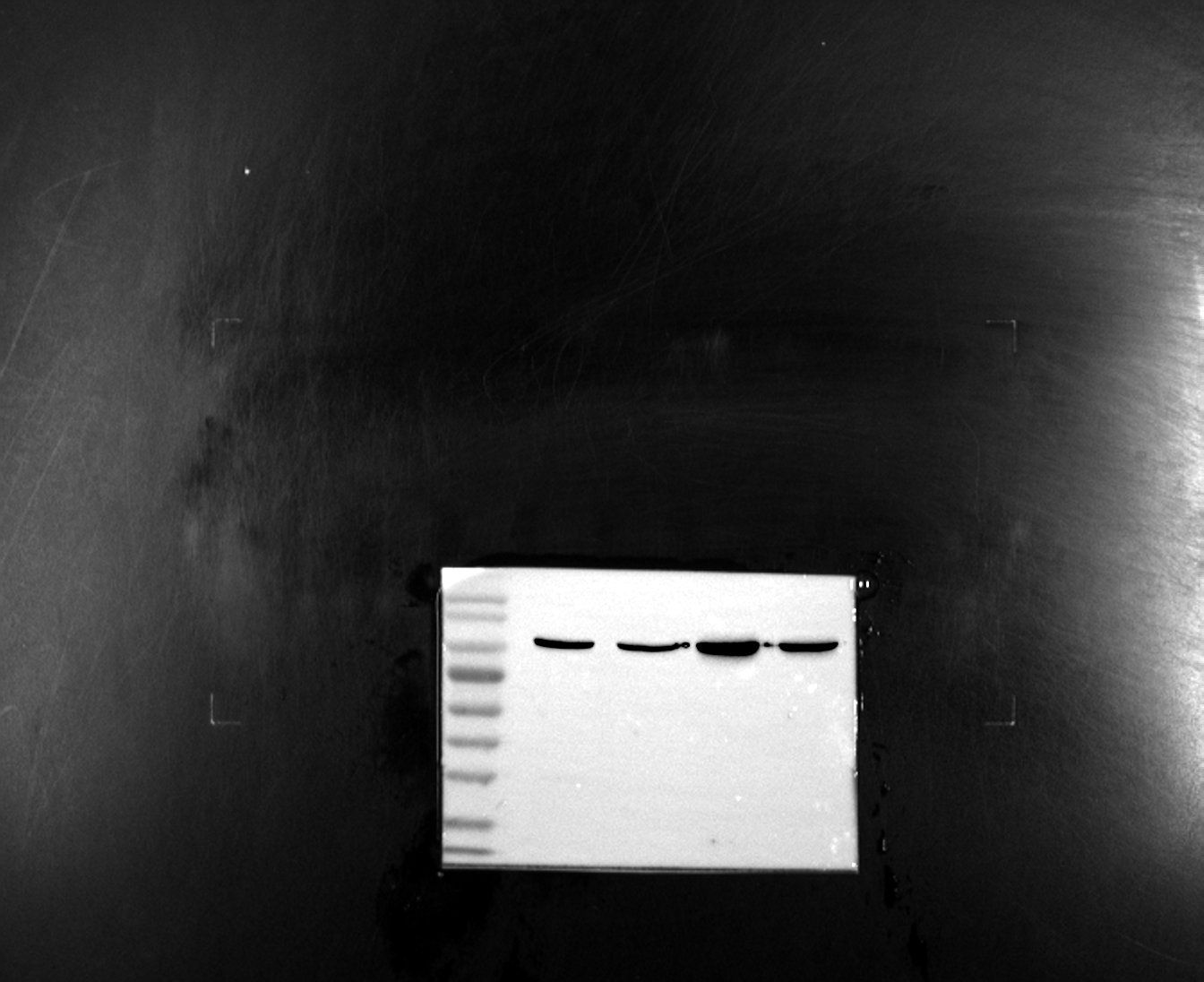

Supplement: S7 File — (ZIP) [file pone.0325936.s007.zip › Raw data 4/Figure 5/images/5C/Nrf2.tif]

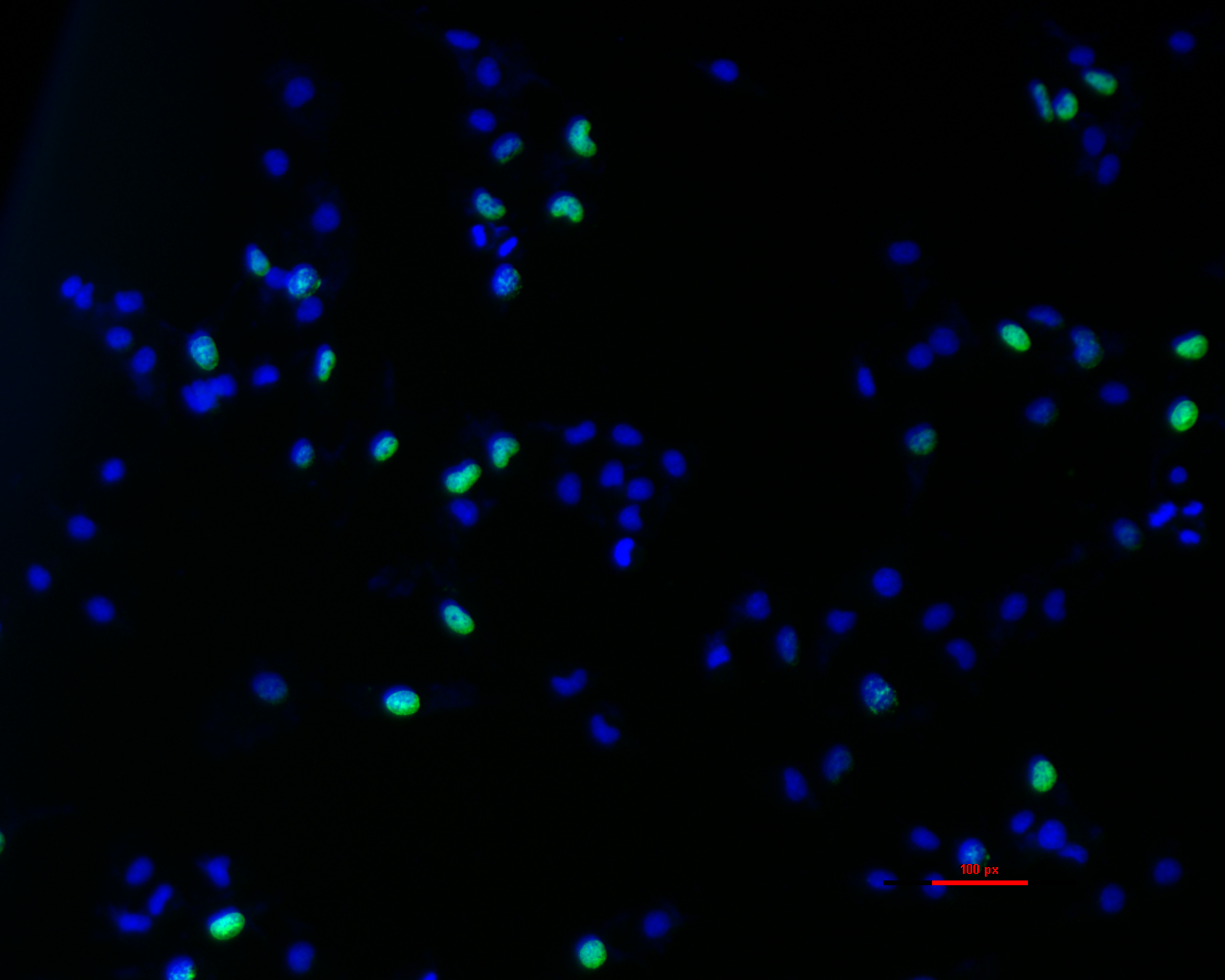

Supplement: S7 File — (ZIP) [file pone.0325936.s007.zip › Raw data 4/Figure 5/images/5E/Control.tif]

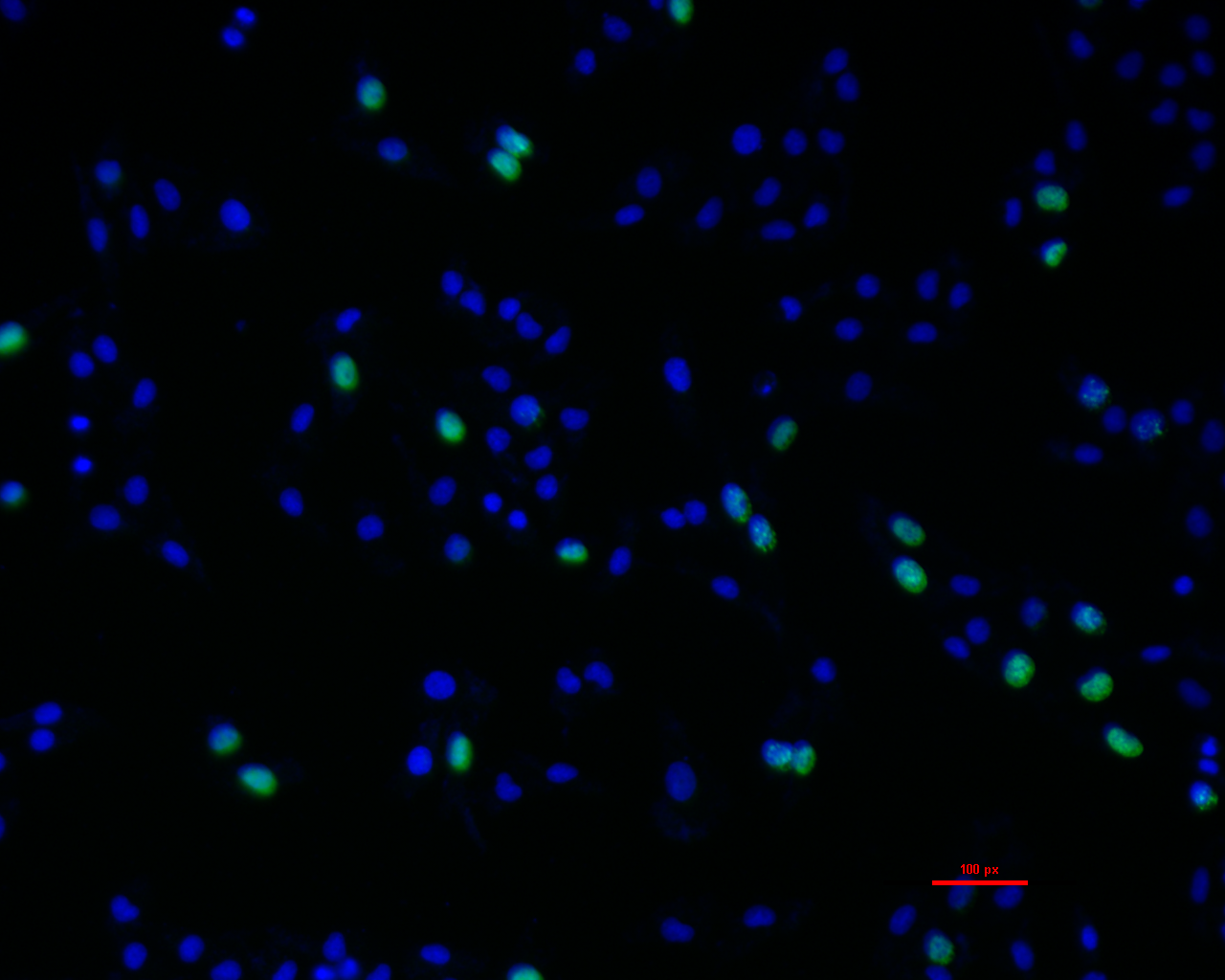

Supplement: S7 File — (ZIP) [file pone.0325936.s007.zip › Raw data 4/Figure 5/images/5E/OE-NC.tif]

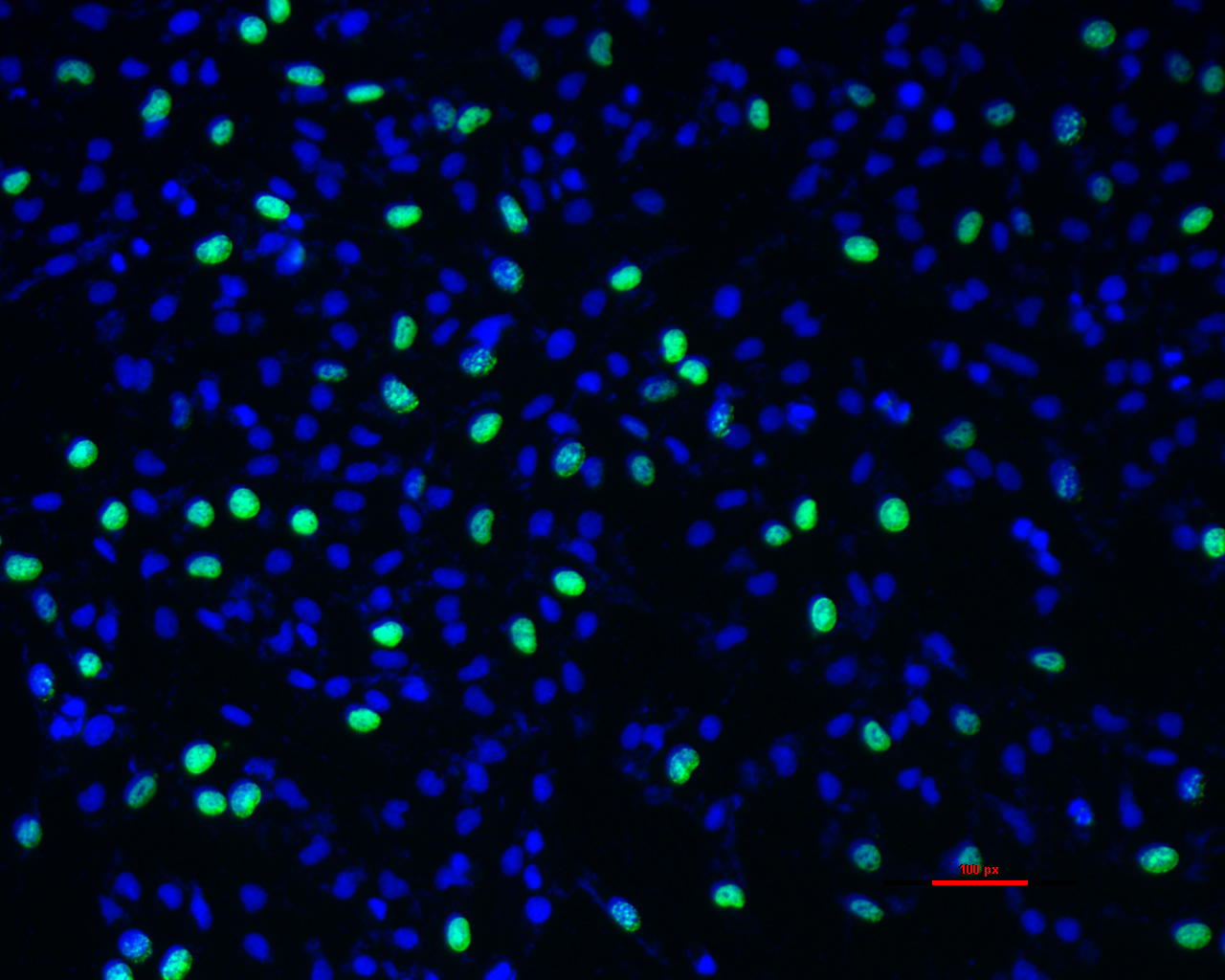

Supplement: S7 File — (ZIP) [file pone.0325936.s007.zip › Raw data 4/Figure 5/images/5E/OE-PLZF+si-Nrf2.tif]

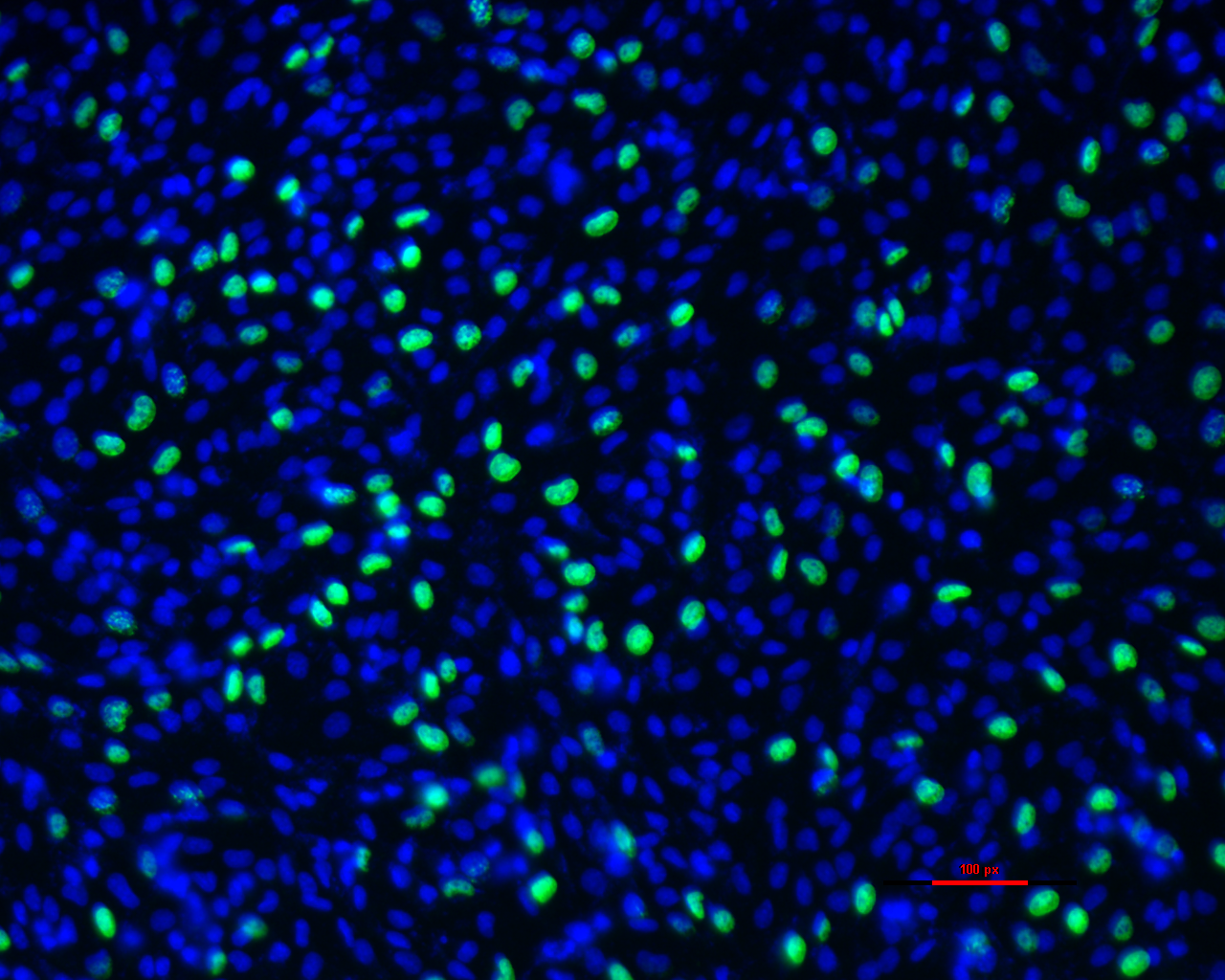

Supplement: S7 File — (ZIP) [file pone.0325936.s007.zip › Raw data 4/Figure 5/images/5E/OE-PLZF.tif]

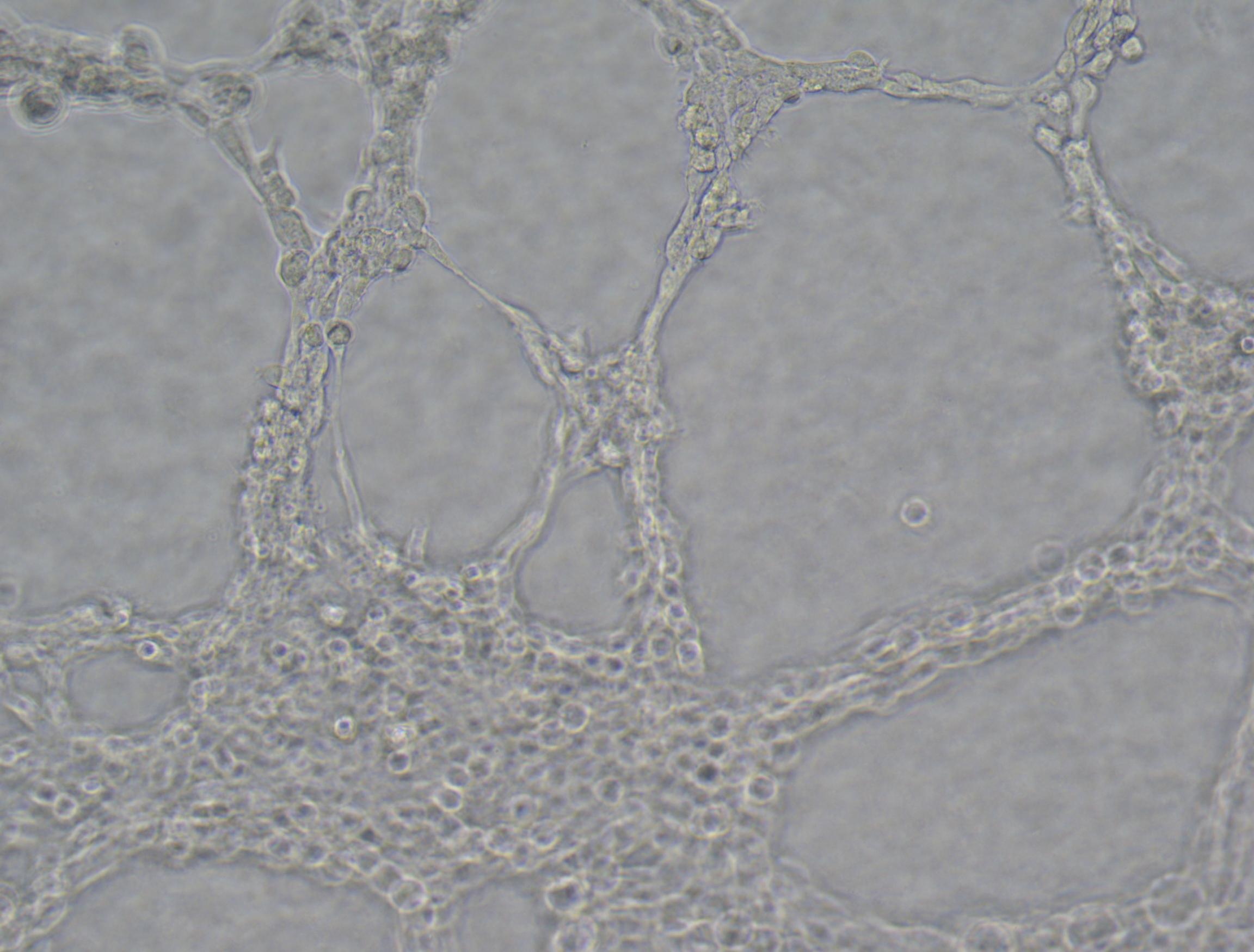

Supplement: S7 File — (ZIP) [file pone.0325936.s007.zip › Raw data 4/Figure 5/images/5F/Control.tif]

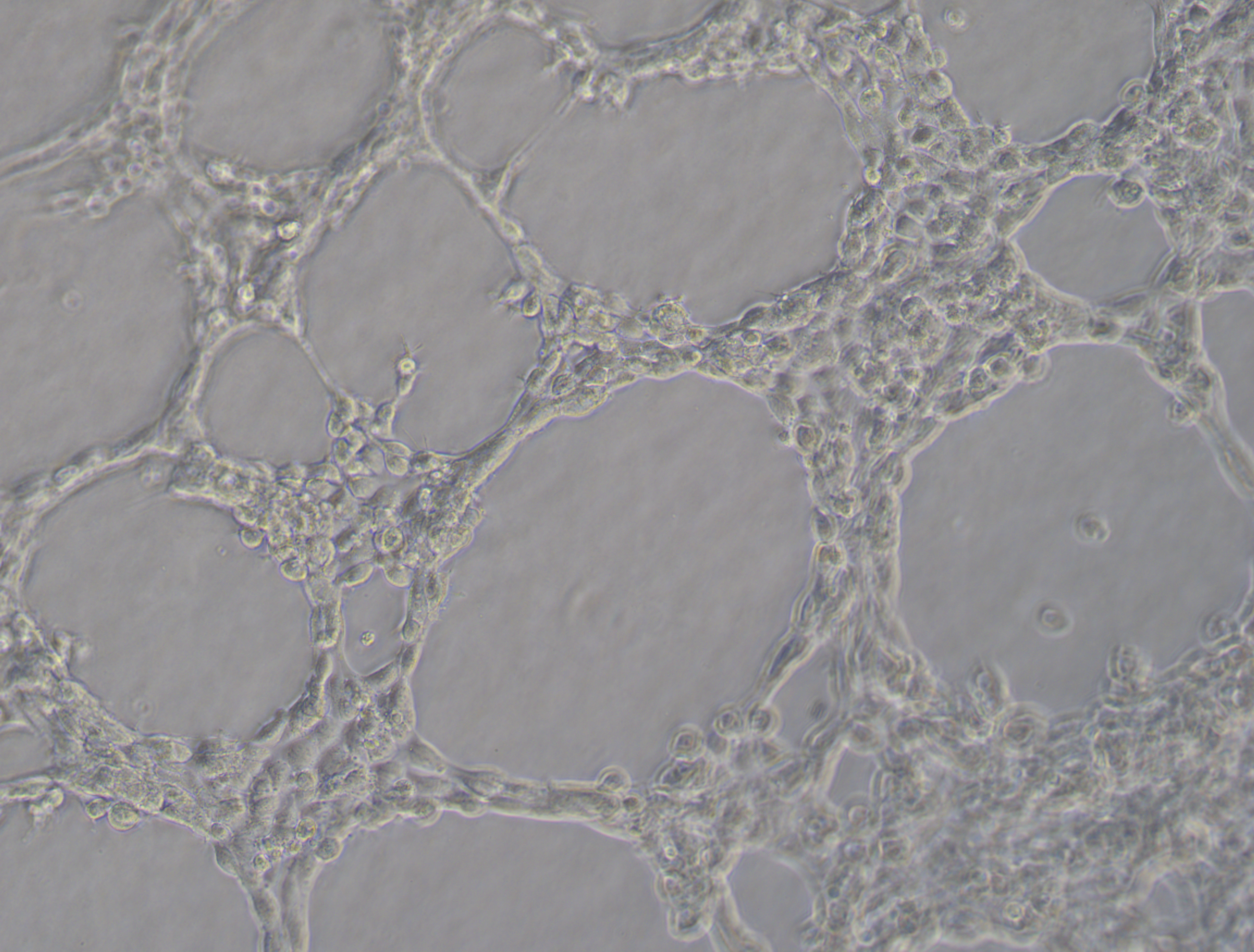

Supplement: S7 File — (ZIP) [file pone.0325936.s007.zip › Raw data 4/Figure 5/images/5F/OE-NC.tif]

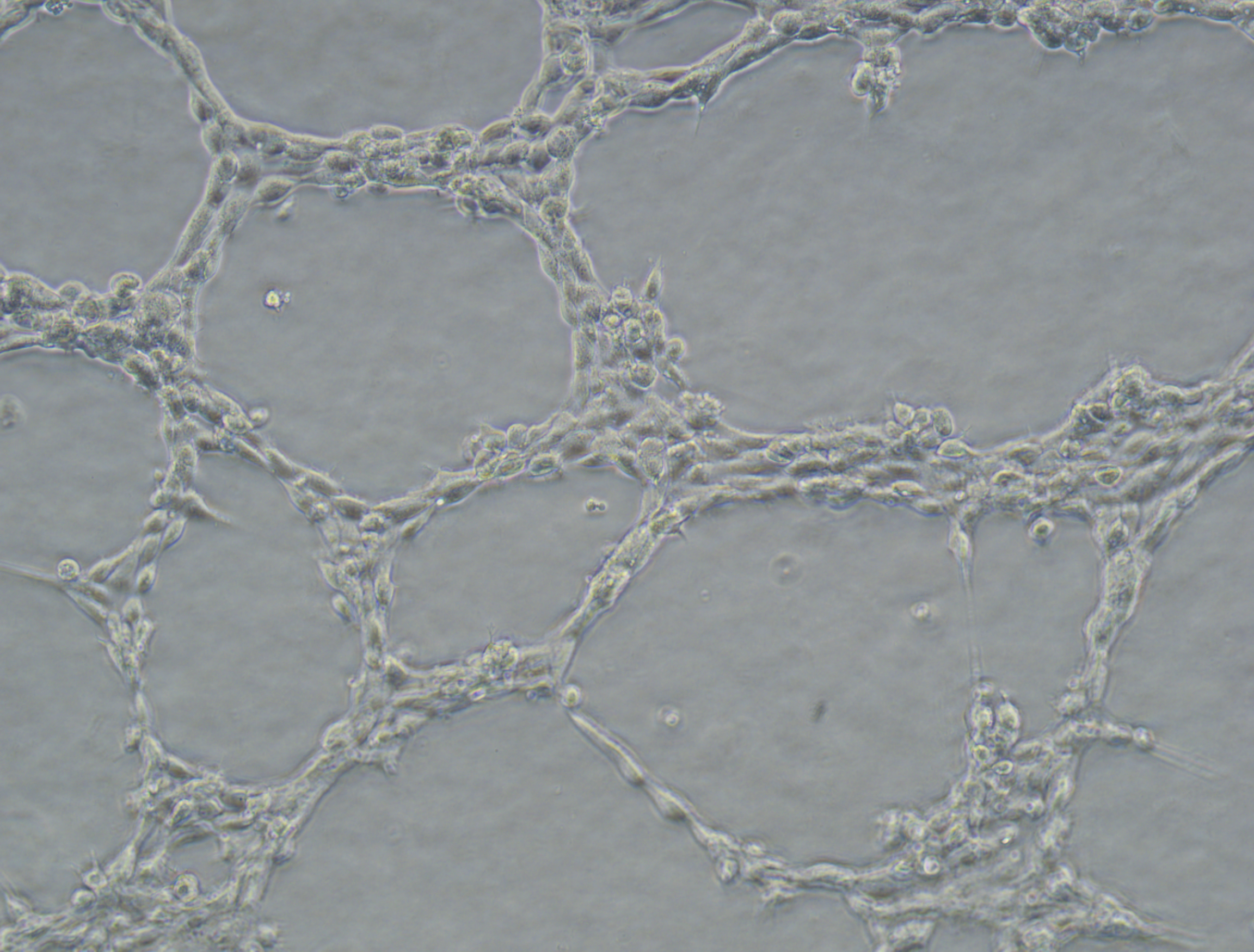

Supplement: S7 File — (ZIP) [file pone.0325936.s007.zip › Raw data 4/Figure 5/images/5F/OE-PLZF+si-Nrf2.tif]

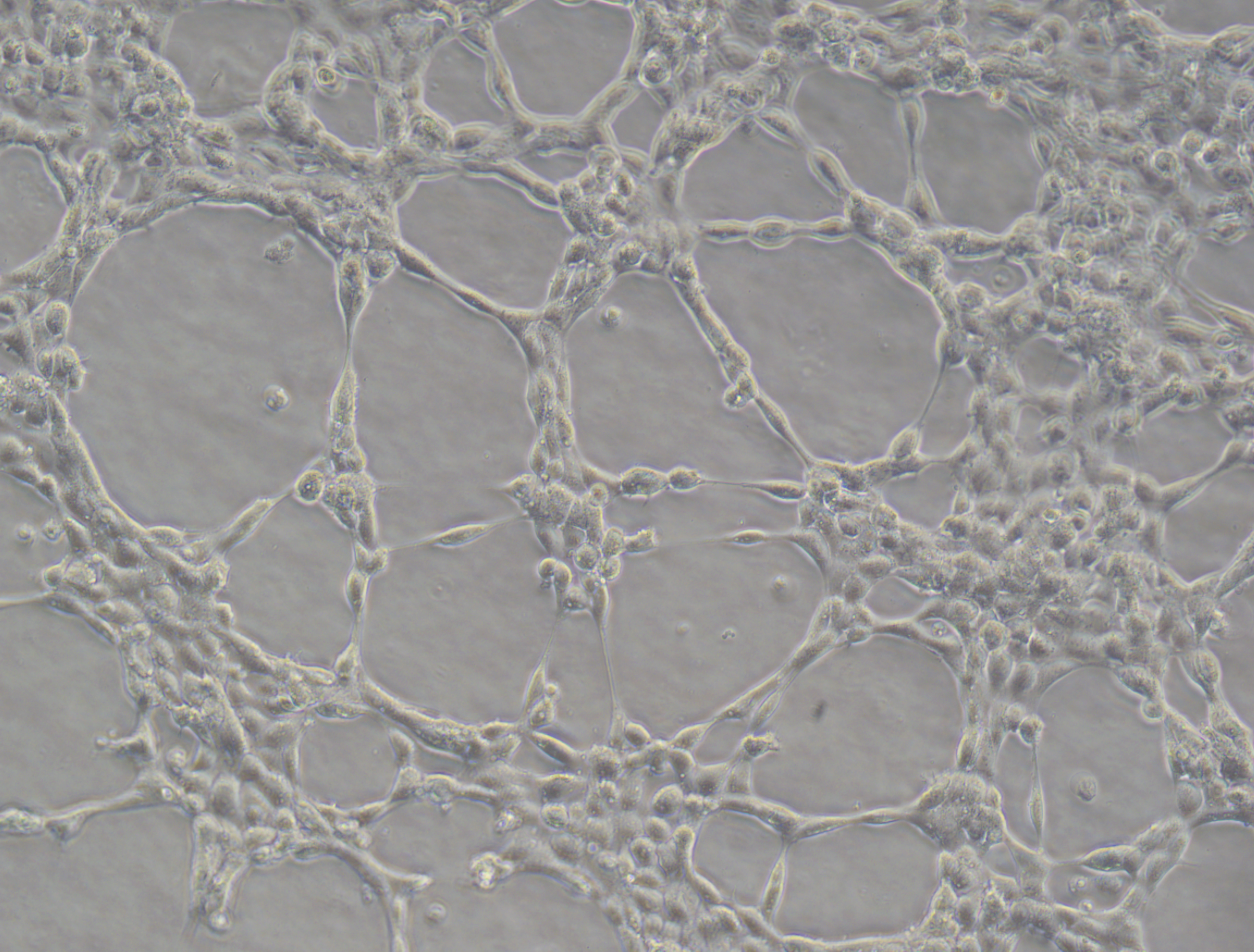

Supplement: S7 File — (ZIP) [file pone.0325936.s007.zip › Raw data 4/Figure 5/images/5F/OE-PLZF.tif]

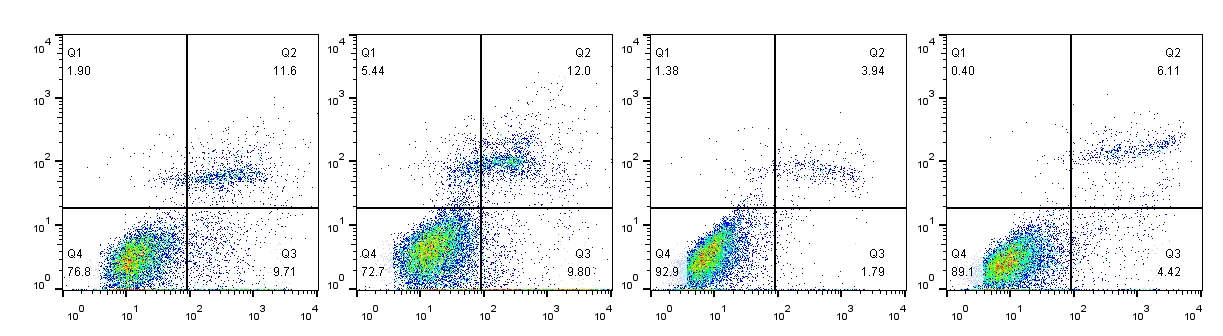

Supplement: S7 File — (ZIP) [file pone.0325936.s007.zip › Raw data 4/Figure 5/images/5G/5G.jpg]

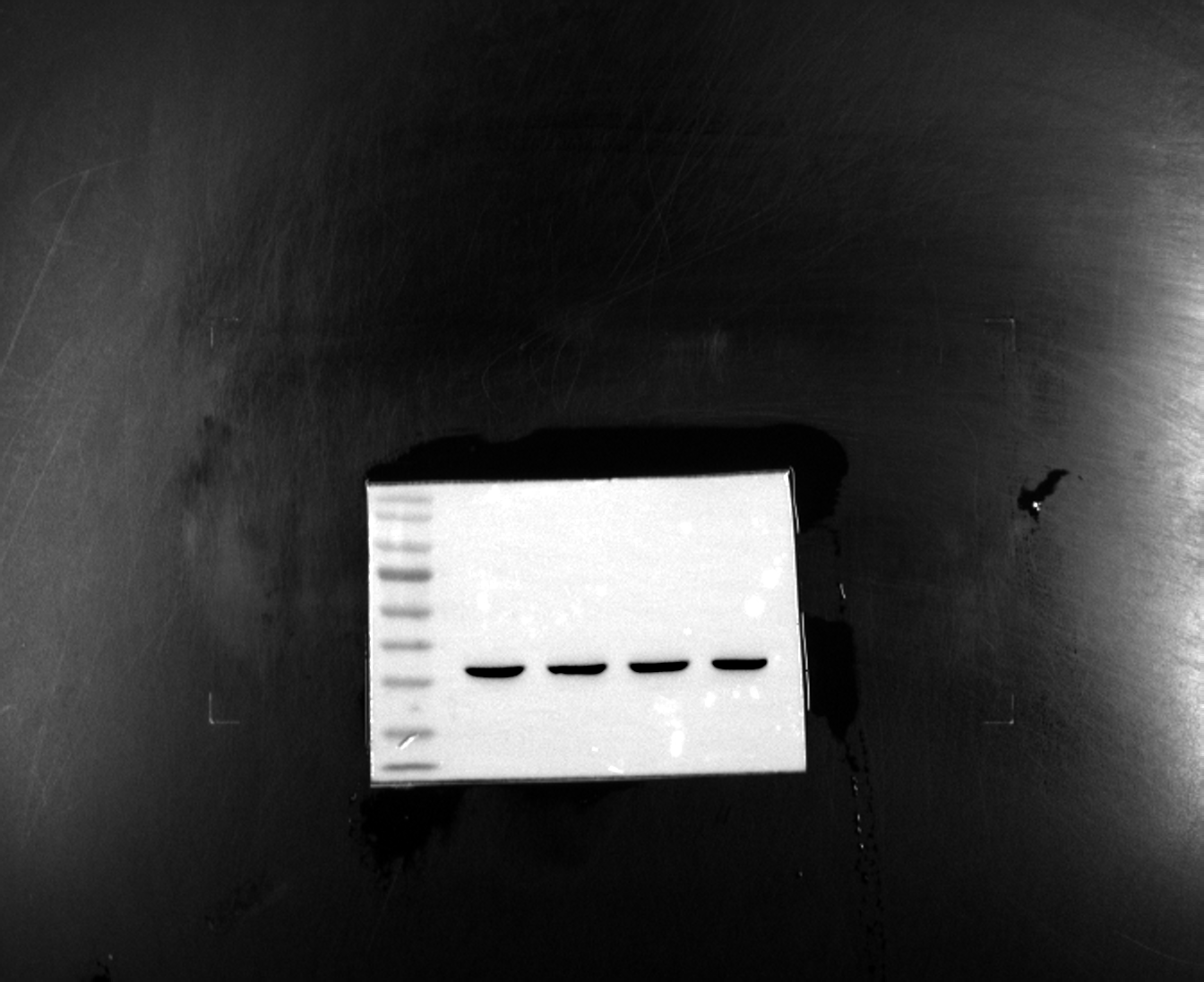

Supplement: S7 File — (ZIP) [file pone.0325936.s007.zip › Raw data 4/Figure 5/images/5H/GAPDH.tif]

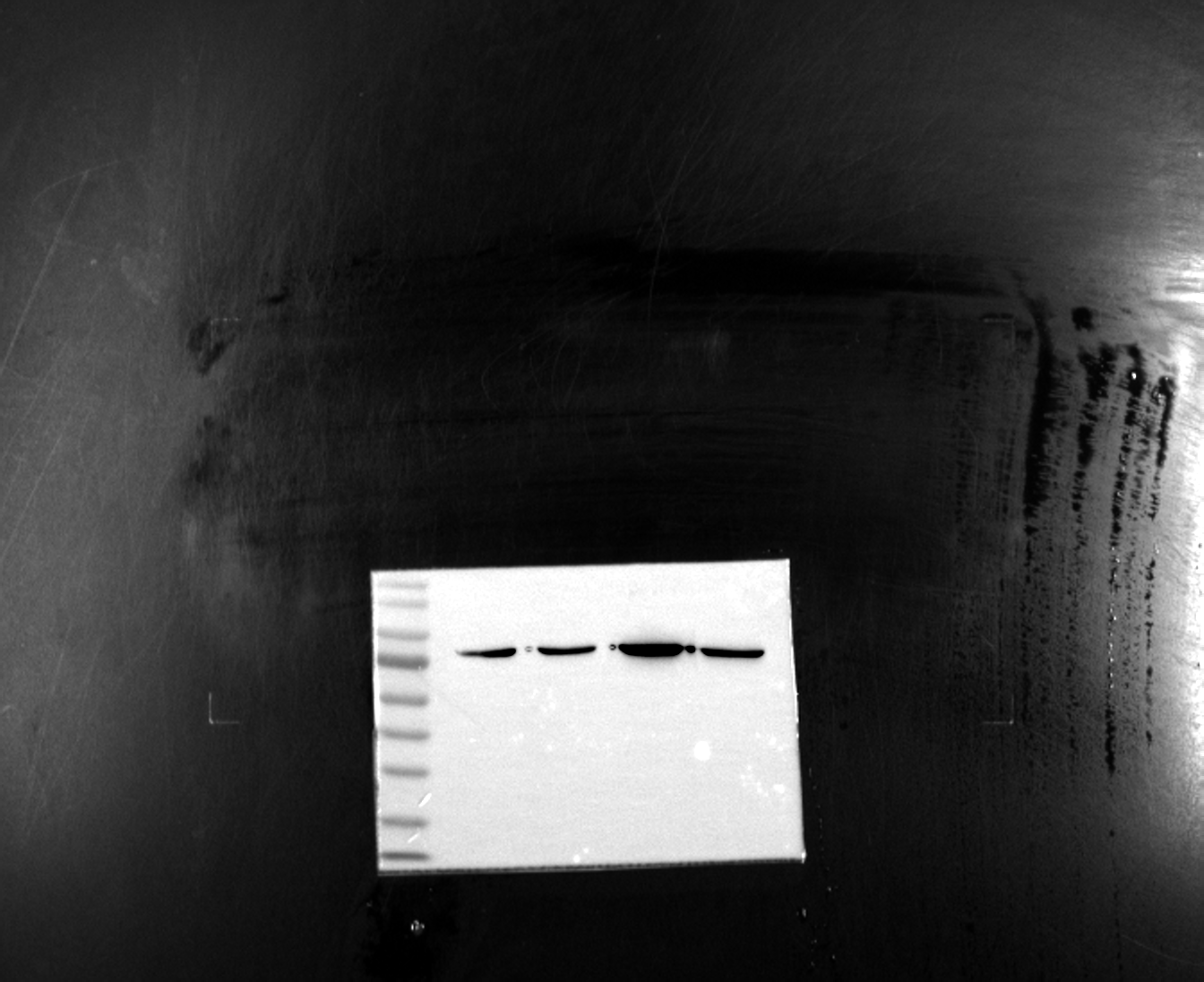

Supplement: S7 File — (ZIP) [file pone.0325936.s007.zip › Raw data 4/Figure 5/images/5H/HIF-1α.tif]

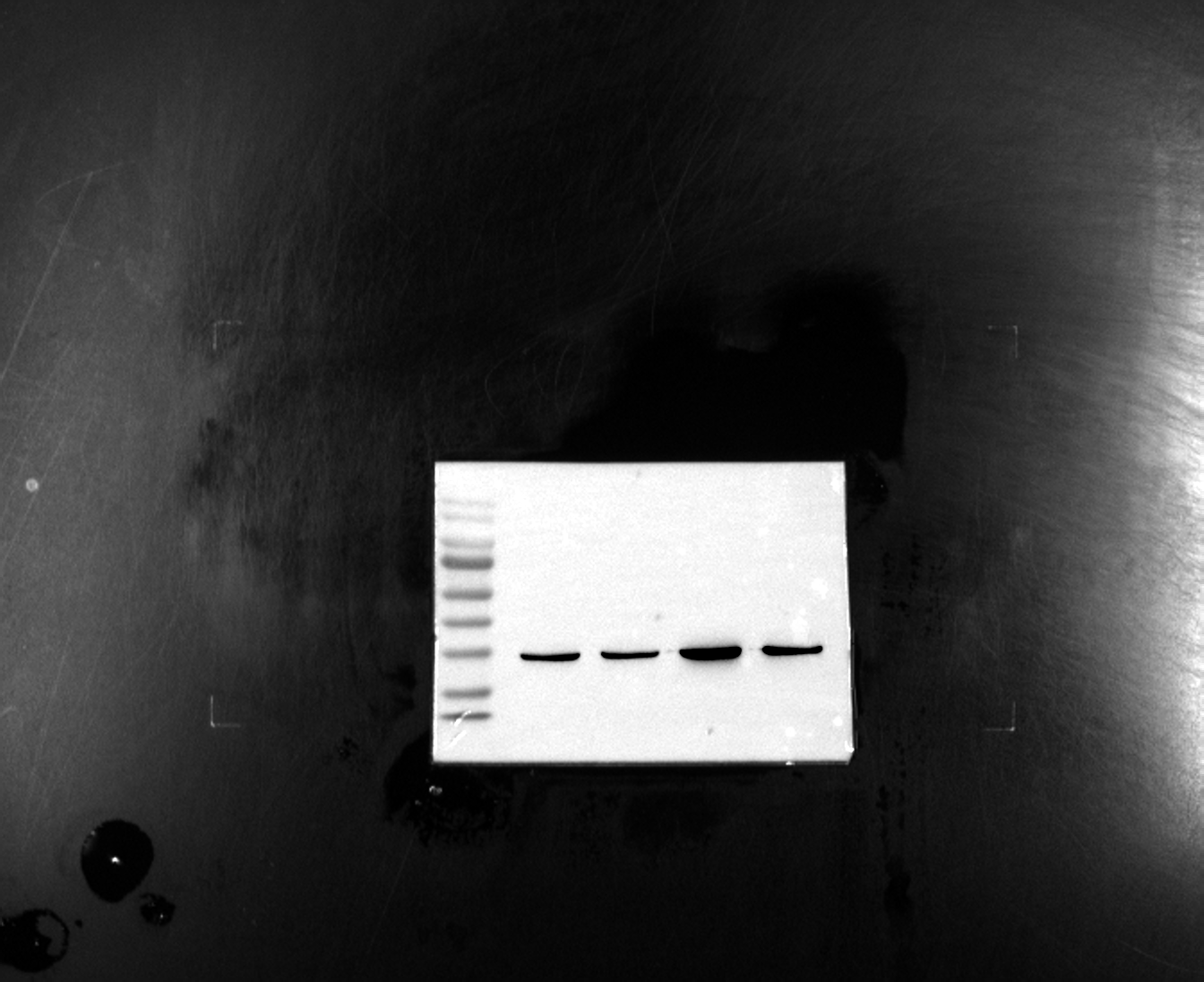

Supplement: S7 File — (ZIP) [file pone.0325936.s007.zip › Raw data 4/Figure 5/images/5H/HO-1.tif]

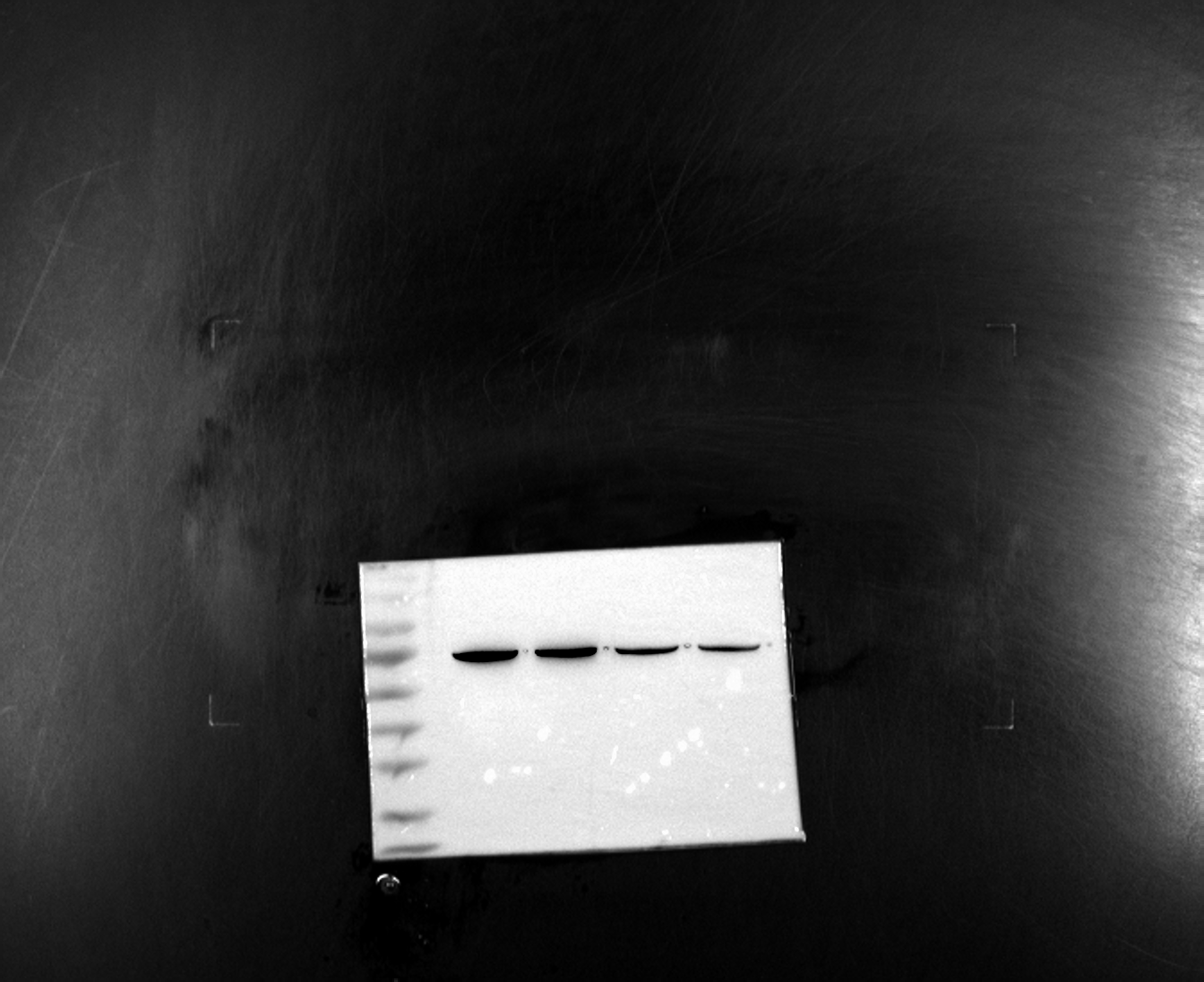

Supplement: S7 File — (ZIP) [file pone.0325936.s007.zip › Raw data 4/Figure 5/images/5H/Keap1.tif]

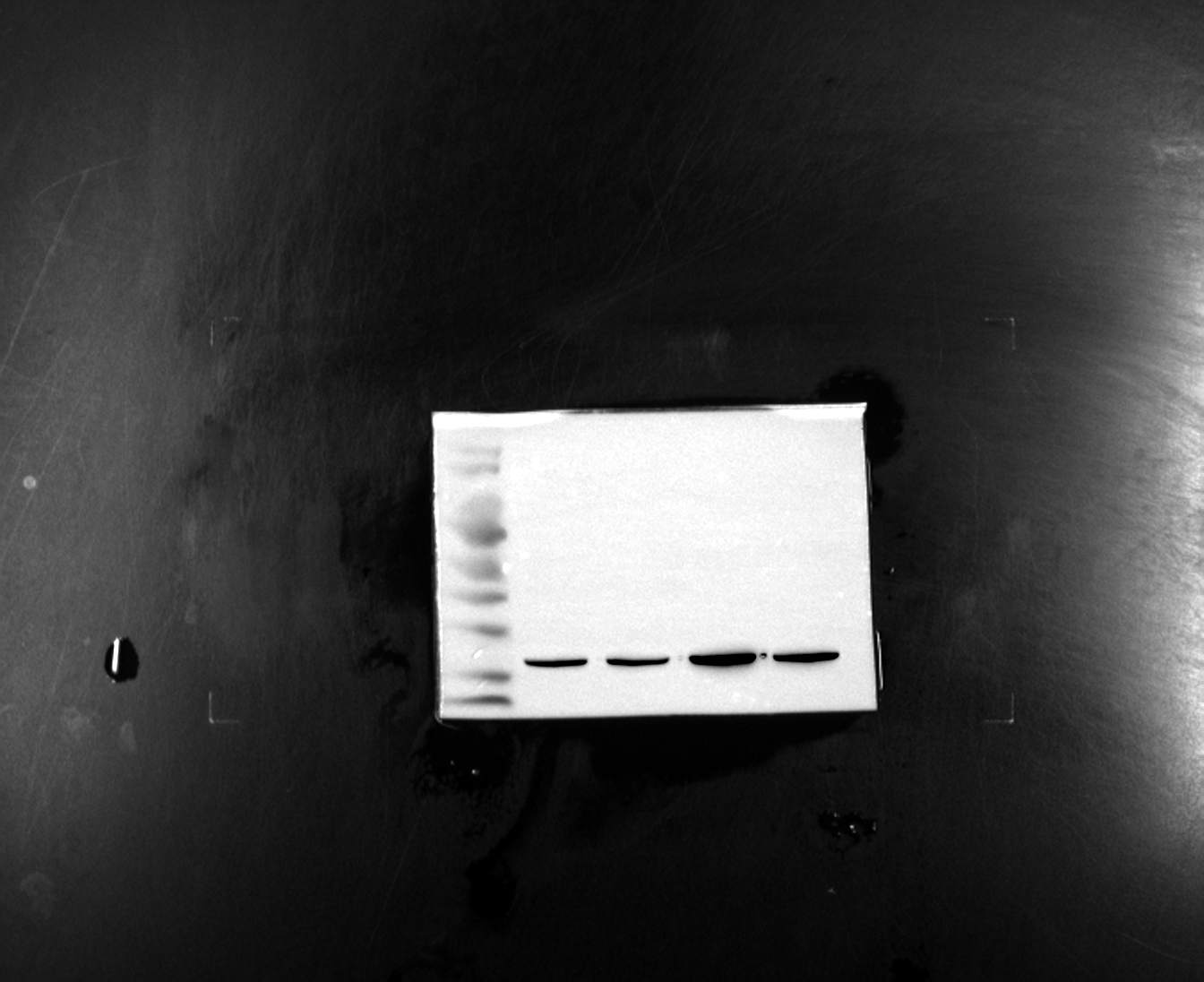

Supplement: S7 File — (ZIP) [file pone.0325936.s007.zip › Raw data 4/Figure 5/images/5H/NQO1.tif]

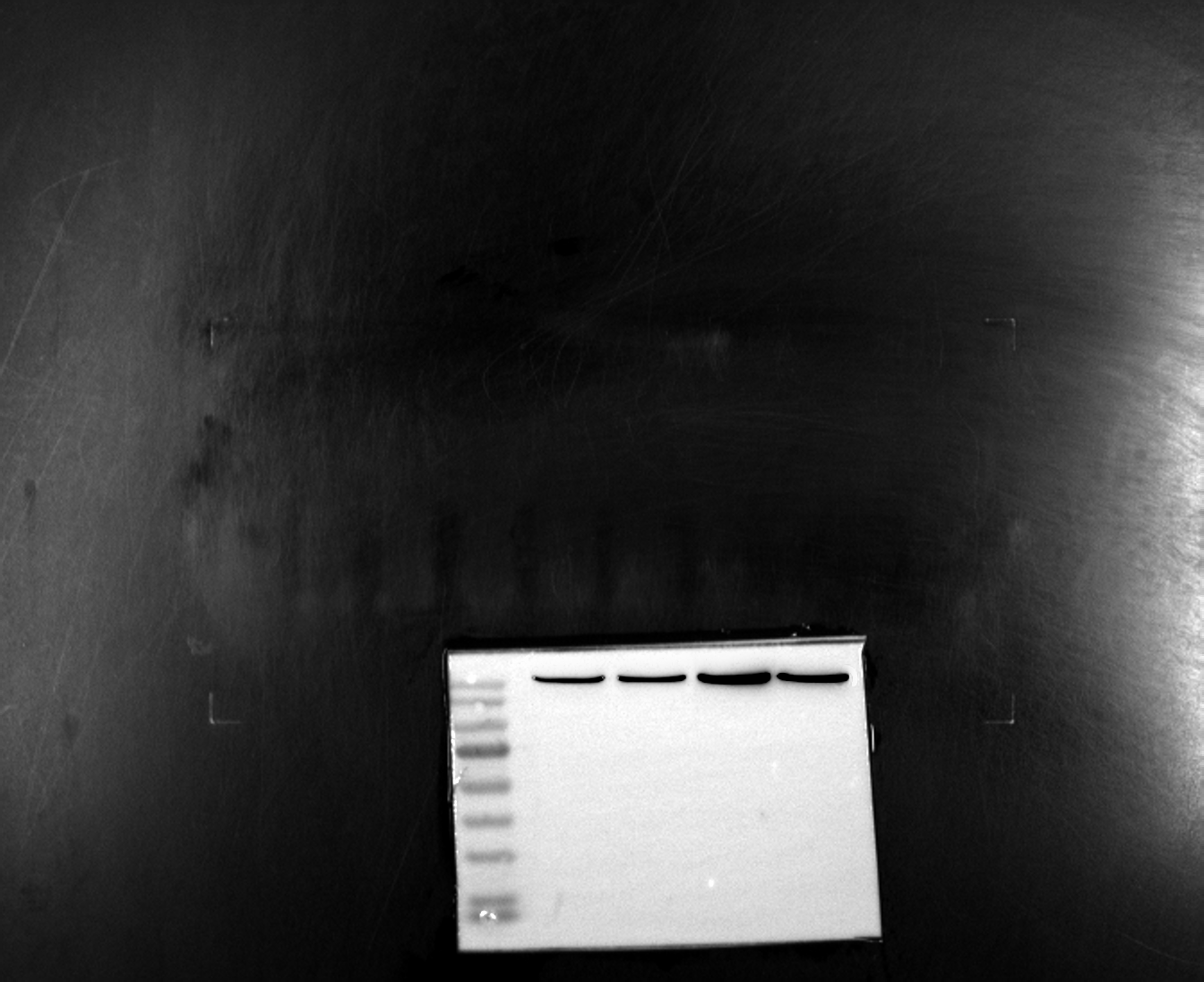

Supplement: S7 File — (ZIP) [file pone.0325936.s007.zip › Raw data 4/Figure 5/images/5H/VEGF.tif]

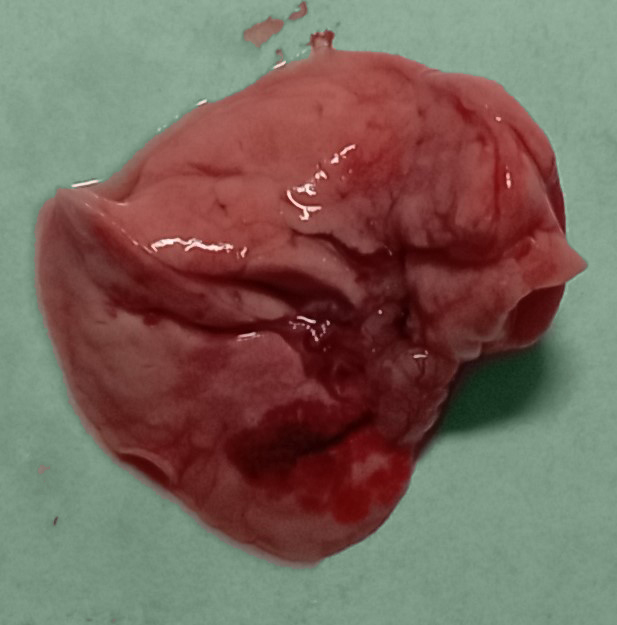

Supplement: S8 File — (ZIP) [file pone.0325936.s008.zip › Raw data 5/Figure 6/images/6A/PNX+OE-PLZF.png]

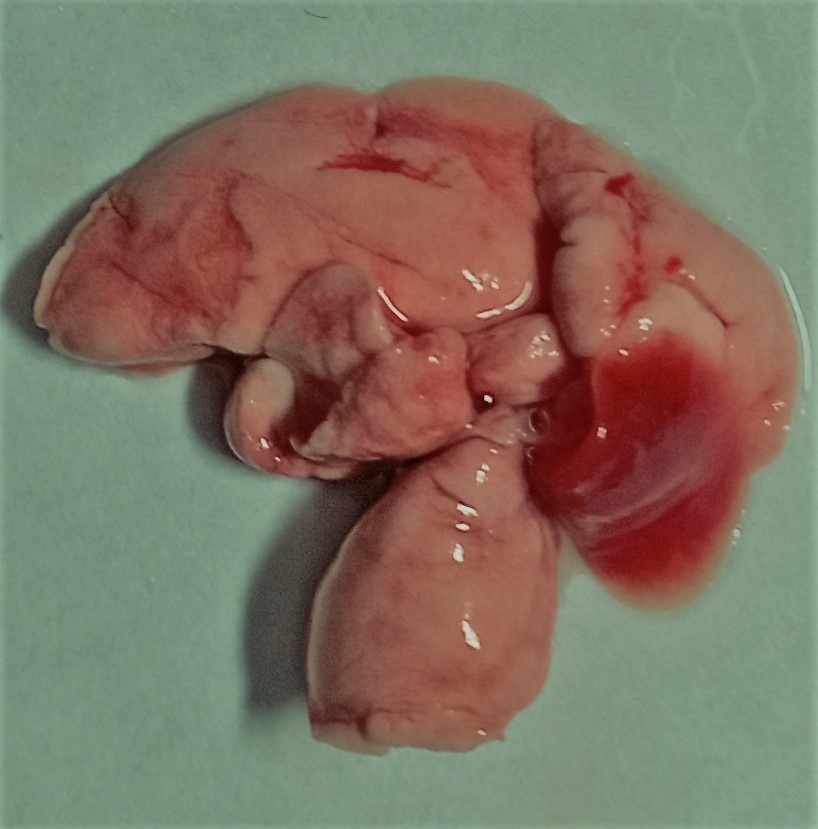

Supplement: S8 File — (ZIP) [file pone.0325936.s008.zip › Raw data 5/Figure 6/images/6A/PNX+pcDNA3.1 vector.jpg]

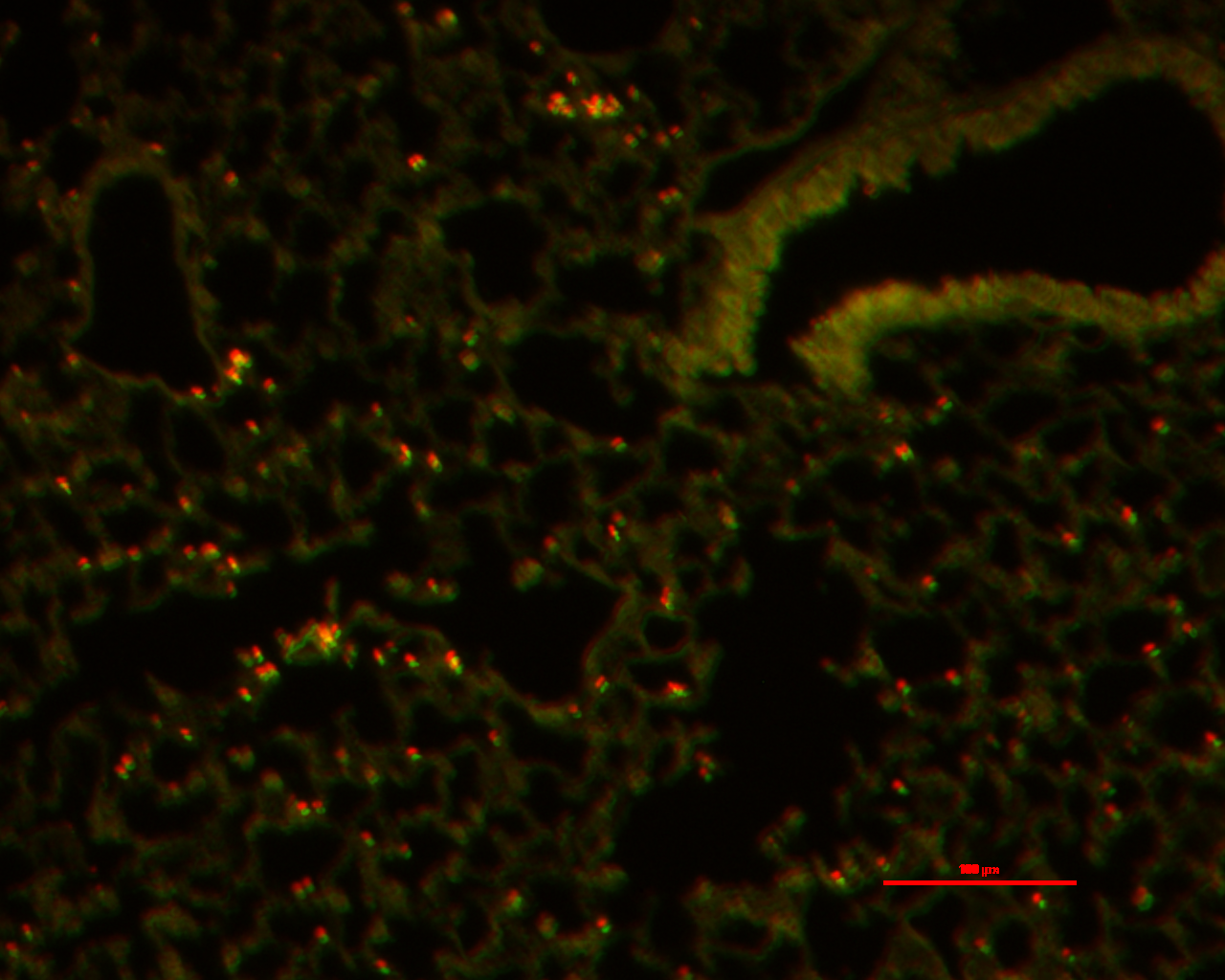

Supplement: S8 File — (ZIP) [file pone.0325936.s008.zip › Raw data 5/Figure 6/images/6C/PNX+OE-PLZF.tif]

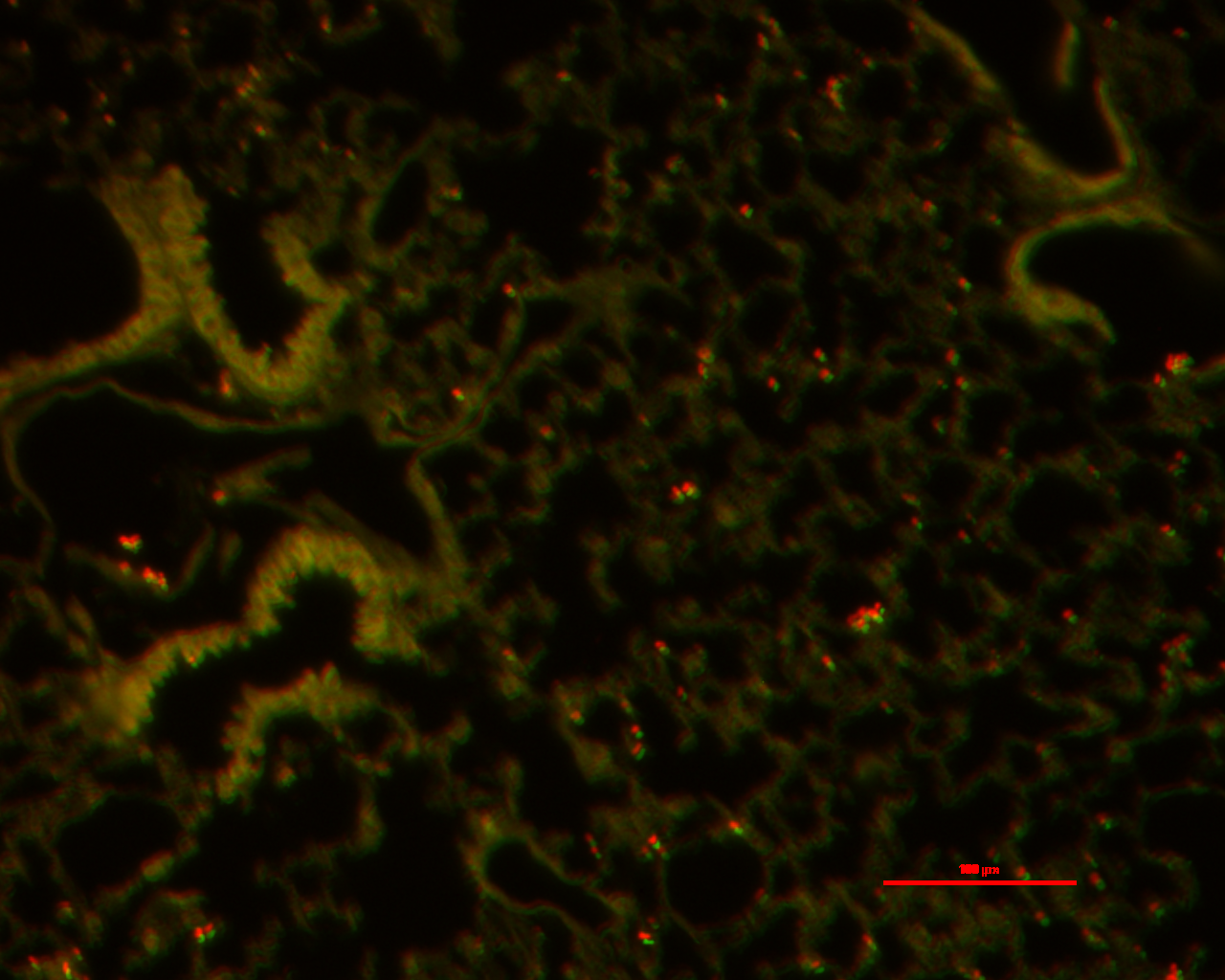

Supplement: S8 File — (ZIP) [file pone.0325936.s008.zip › Raw data 5/Figure 6/images/6C/PNX+pcDNA3.1 vector.tif]

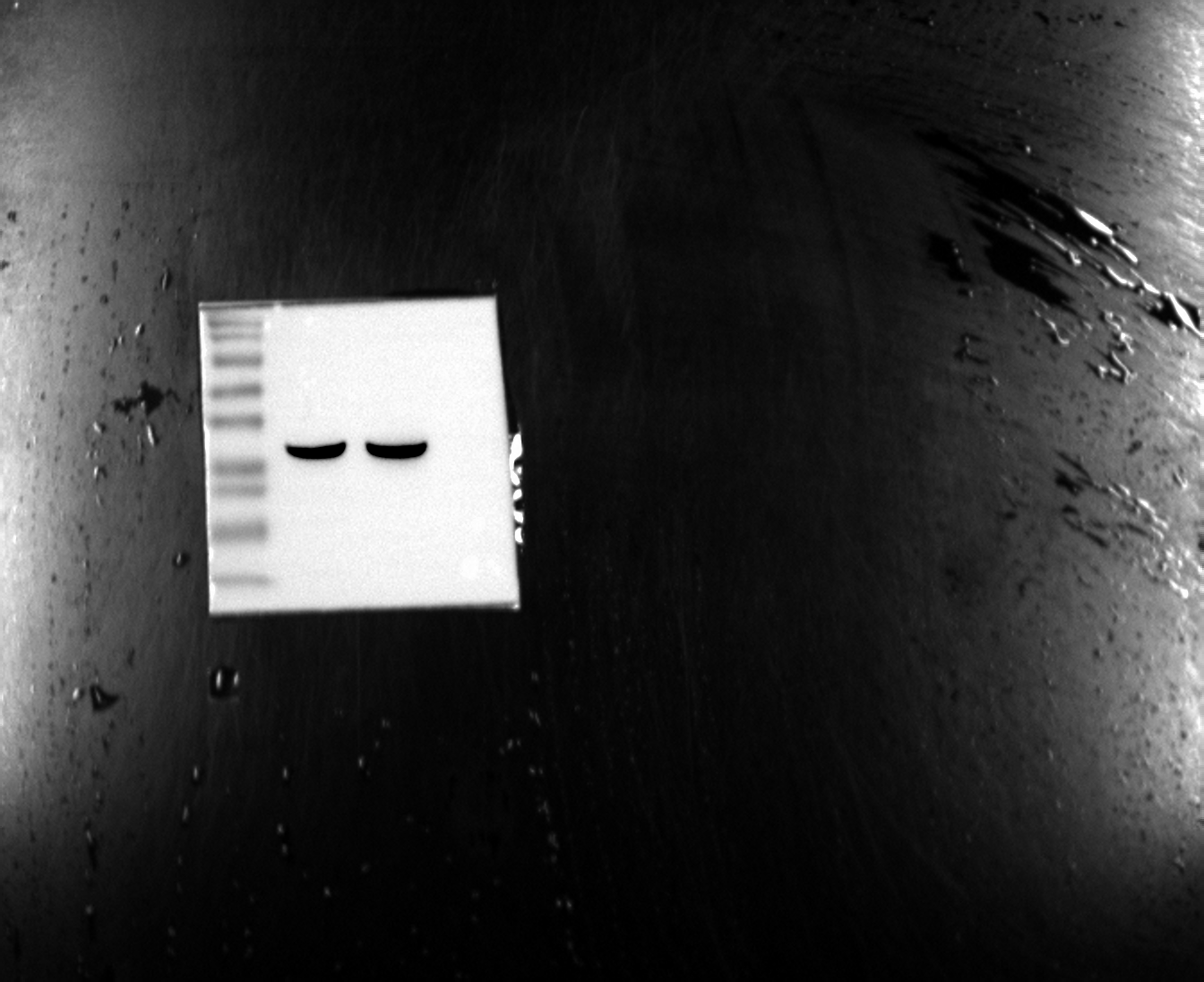

Supplement: S8 File — (ZIP) [file pone.0325936.s008.zip › Raw data 5/Figure 6/images/6D/GAPDH.tif]

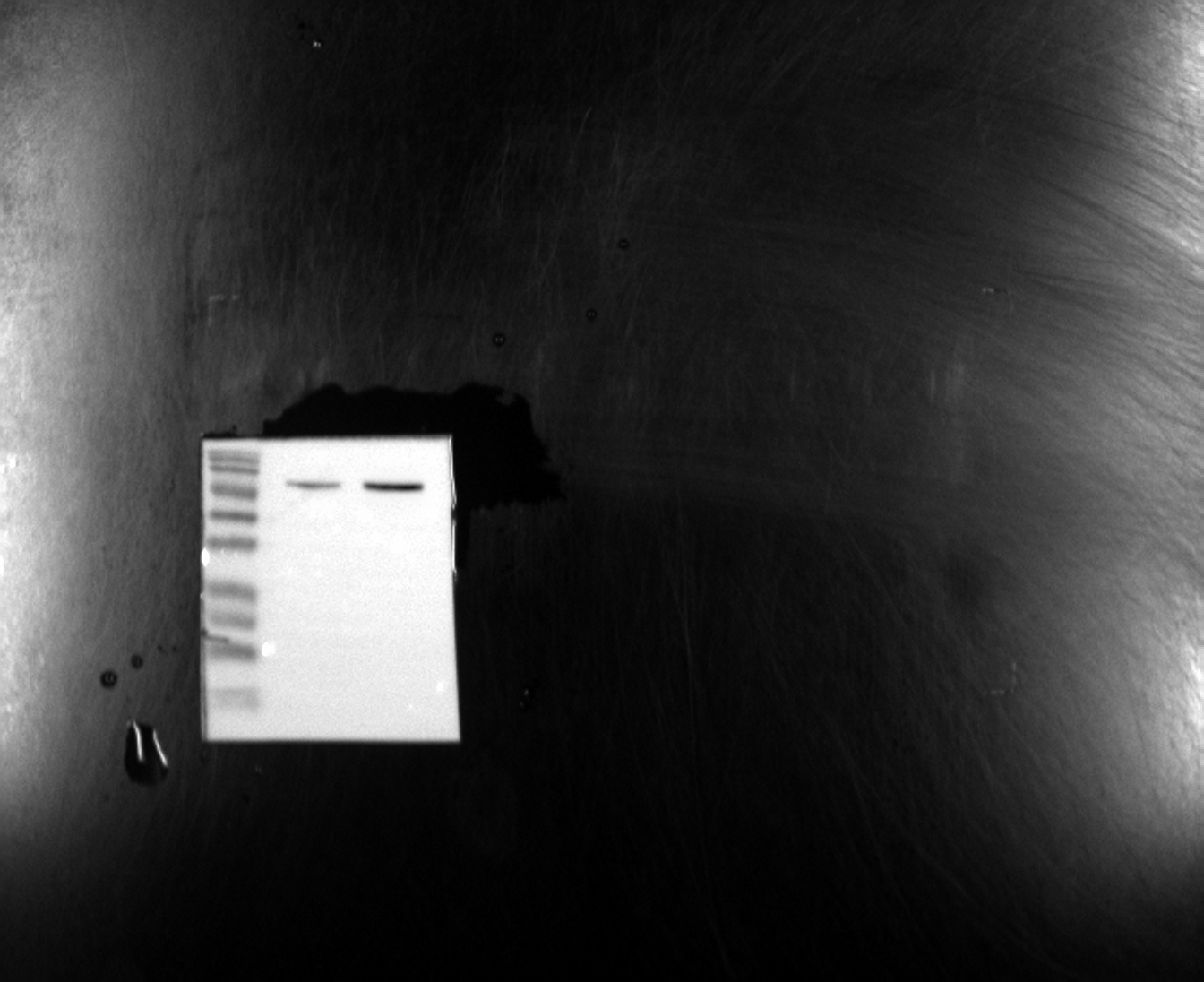

Supplement: S8 File — (ZIP) [file pone.0325936.s008.zip › Raw data 5/Figure 6/images/6D/HIF-1α.tif]

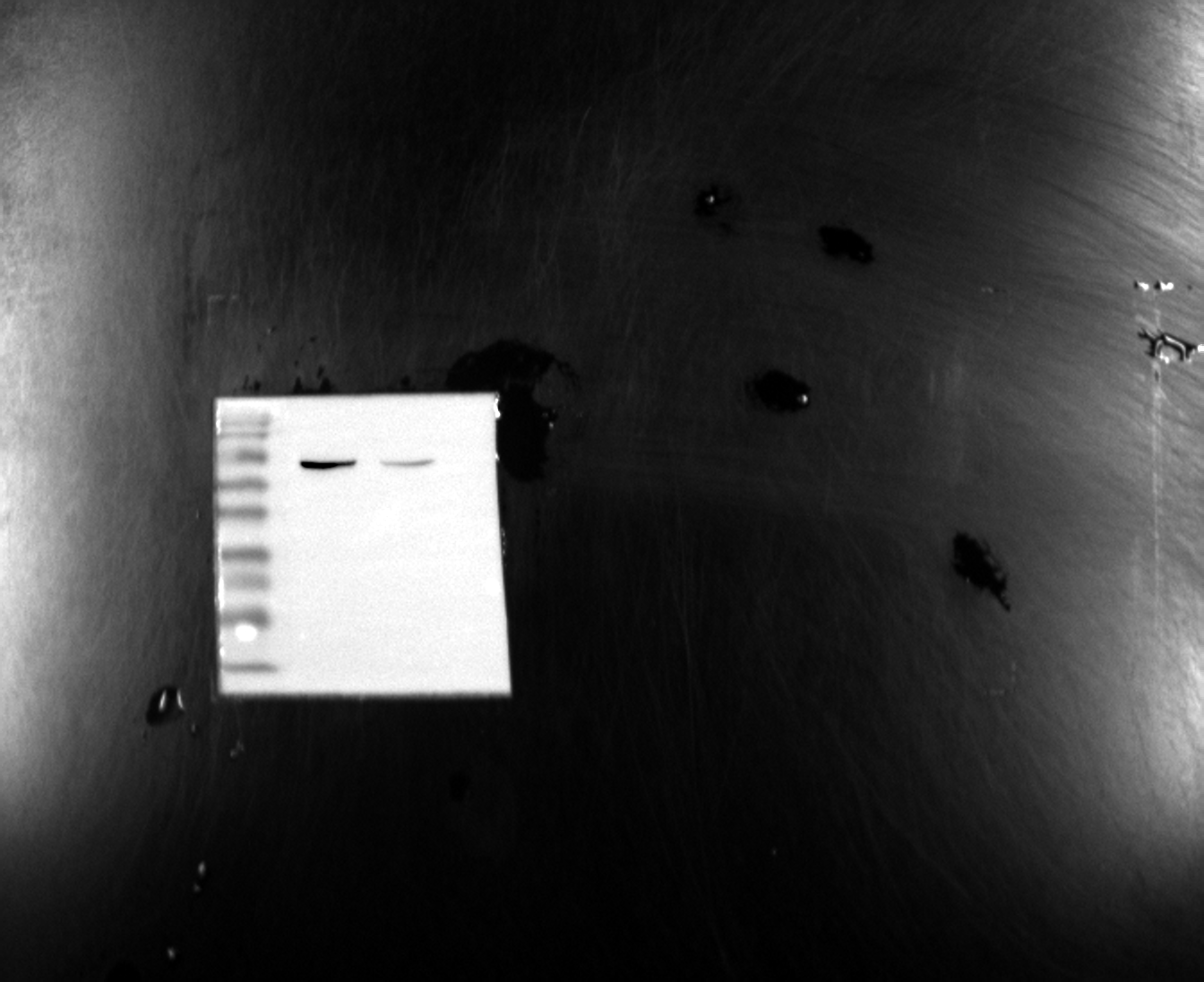

Supplement: S8 File — (ZIP) [file pone.0325936.s008.zip › Raw data 5/Figure 6/images/6D/Keap1.tif]

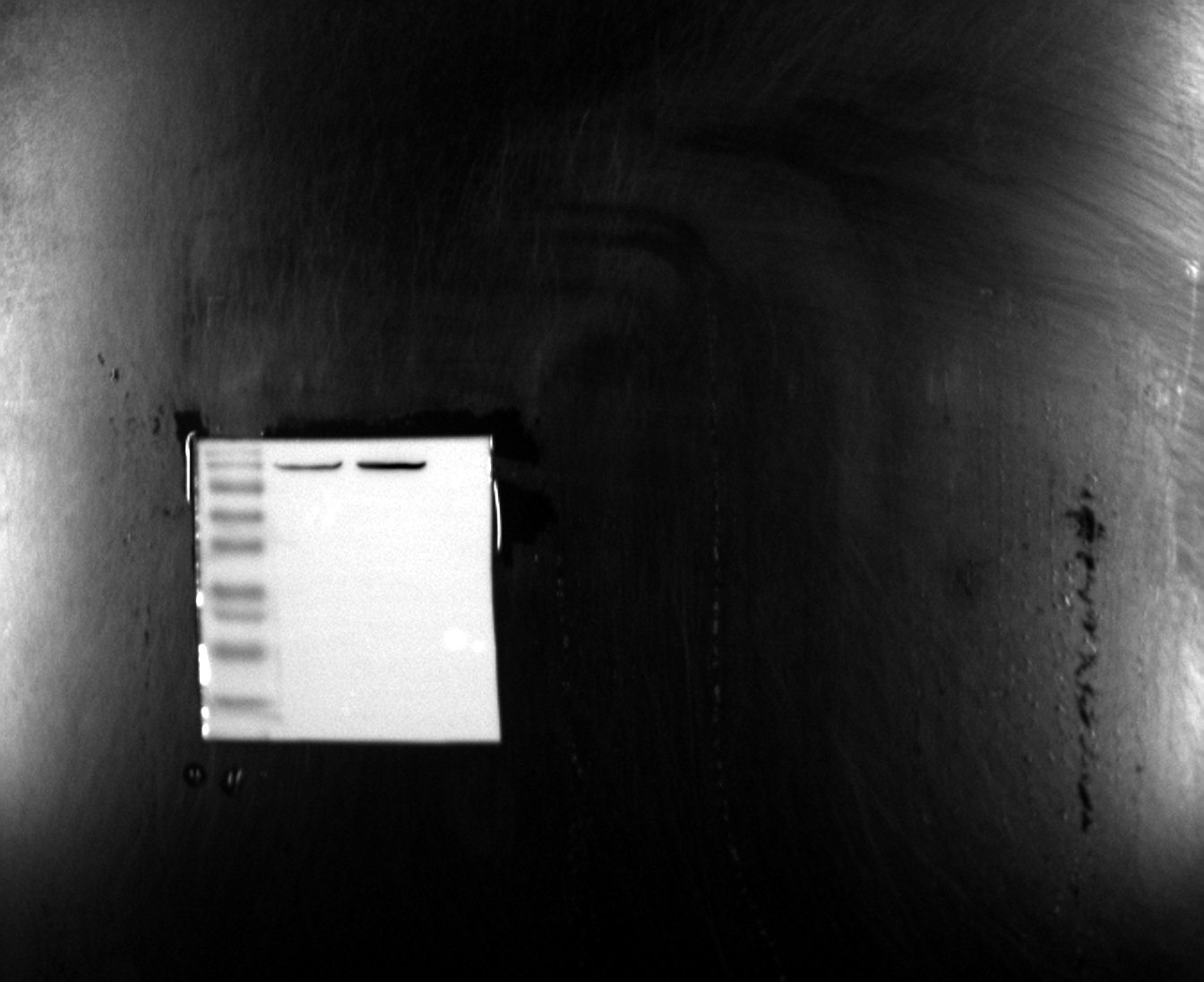

Supplement: S8 File — (ZIP) [file pone.0325936.s008.zip › Raw data 5/Figure 6/images/6D/Nrf2.tif]

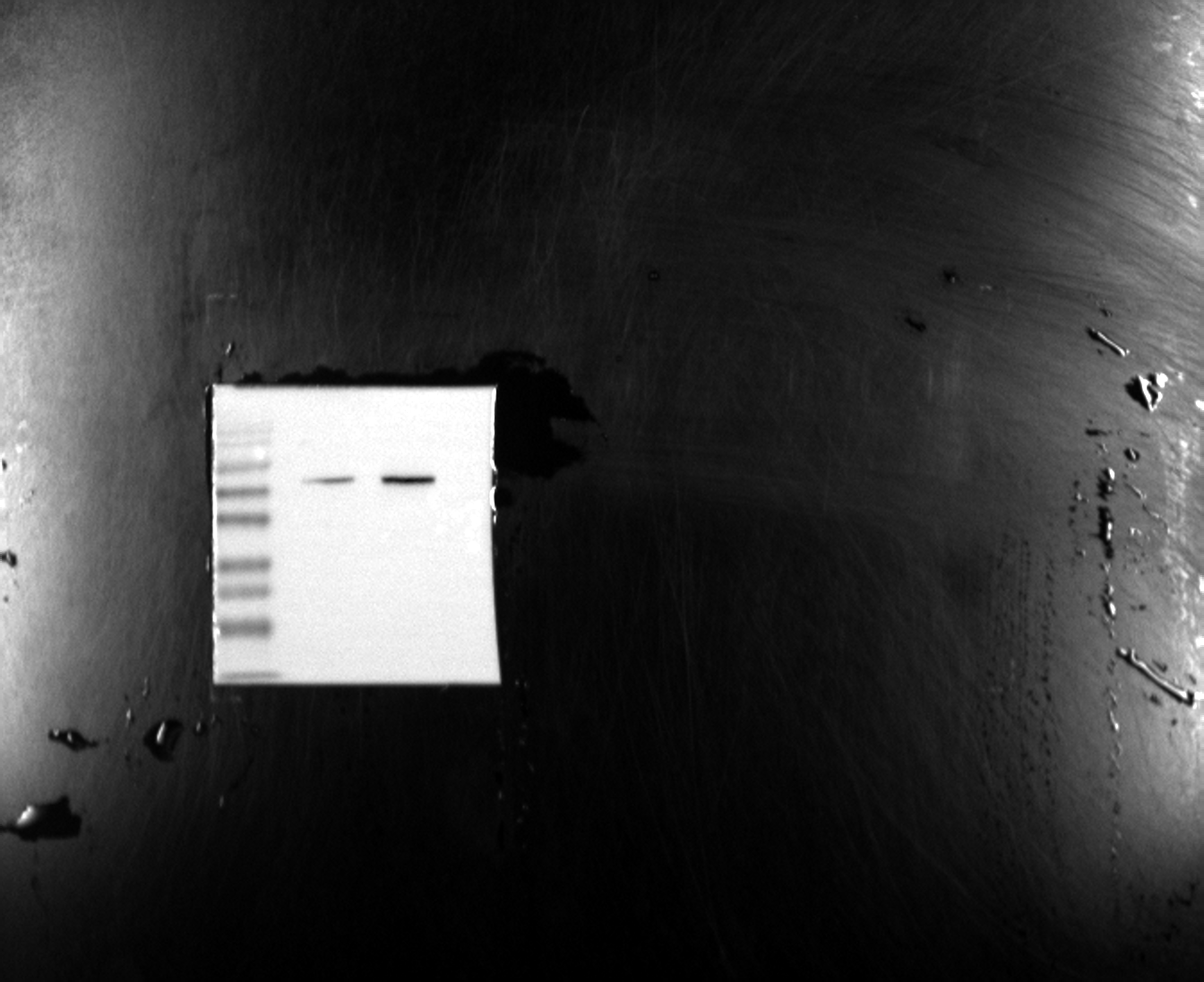

Supplement: S8 File — (ZIP) [file pone.0325936.s008.zip › Raw data 5/Figure 6/images/6D/PLZF.tif]

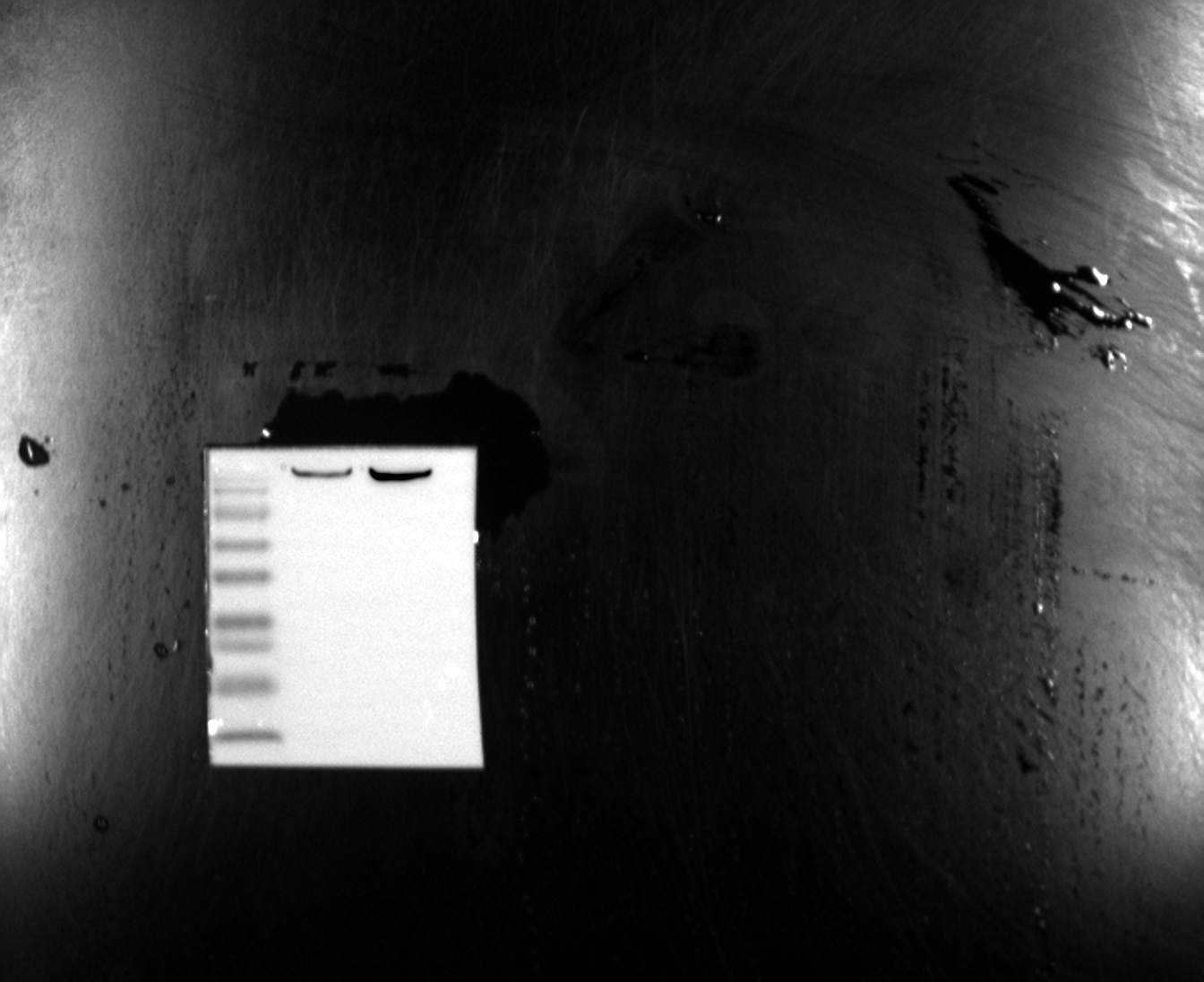

Supplement: S8 File — (ZIP) [file pone.0325936.s008.zip › Raw data 5/Figure 6/images/6D/VEGF.tif]
